# Molecular and Cellular Effects of Microplastics and Nanoplastics in the Pathogenesis of Cardiovascular, Nervous, Urinary, Digestive, and Reproductive System Diseases: A Global Systematic Review

**DOI:** 10.3390/ijms262211194

**Published:** 2025-11-19

**Authors:** Vasilii Chulkov, Mitkhat Gasanov, Vladimir Isakov, Anastasia Denisenko, Chizaram Nwosu, Stanislav Rodkin

**Affiliations:** 1 Department of Hospital Therapy, Yaroslav-the-Wise Novgorod State University, Derzhavina St. 6, Veliky Novgorod 173020, Russia; 2 Microplastics Research Center, Chemical Engineering Institute, Yaroslav-the-Wise Novgorod State University, Veliky Novgorod 173003, Russia; 3 Research Laboratory “Medical Digital Images Based on the Basic Model”, Department of Bioengineering, Institute of Living Systems, Don State Technical University, Rostov-on-Don 344000, Russia

**Keywords:** microplastics, nanoplastics, oxidative stress, inflammation, mitochondrial dysfunction, apoptosis, autophagy, ferroptosis, barrier dysfunction, cardiovascular system, nervous system, reproductive system, urinary system, digestive system, dysbiosis, toxicity, environmental pollution, bioaccumulation

## Abstract

Microplastics (MPs) and nanoplastics (NPs), formed as a result of plastic product degradation, pose a global environmental threat by penetrating biological systems and inducing systemic pathological changes. This systematic review, conducted in accordance with the Preferred Reporting Items for Systematic Reviews and Meta-Analyses extension for Scoping Reviews guidelines, aims to analyze the molecular and cellular mechanisms of the toxic effects of MPs and NPs on the human cardiovascular, nervous, reproductive, urinary, and digestive systems. The primary mechanisms include oxidative stress, inflammation, mitochondrial dysfunction, apoptosis, autophagy, ferroptosis, and impaired barrier functions. In the cardiovascular system, MPs and NPs contribute to endothelial dysfunction, disorders of lipid metabolism, and fibrosis; in the nervous system, they promote neuroinflammation, pathological protein aggregation, and psychiatric disorders; in the reproductive system, they lead to hormonal imbalance and reduced fertility; in the kidneys, they cause inflammation, and fibrosis and lead to deterioration of kidney function; and in the gastrointestinal tract, they contribute to dysbiosis and metabolic disorders. The literature search was conducted in the PubMed, Web of Science, and Scopus databases without limitations on date, language, or access. Studies were selected based on criteria of transparency, statistical validity, sample representativeness, and correctness of data interpretation. The review emphasizes the necessity of an interdisciplinary approach to developing prevention and treatment strategies, including reduction in exposure, antioxidant and immunomodulatory therapy, and restoration of barrier functions and microbiota. The data obtained reveal research gaps and identify directions for further study.

## 1. Introduction

Microplastics (MPs) and nanoplastics (NPs) are products of the degradation of plastic products resulting from industrial and household activities that, by penetrating all levels of the biosphere, are taking on the character of an environmental catastrophe [1,2]. These particles micro- and nanoplastics (MNPs) gradually accumulate in biological tissues, entering the body through various routes, ranging from airborne inhalation to transdermal penetration. Due to their small sizes, they can easily overcome biological barriers, accumulating in the blood [3] and cerebrospinal fluid [4], as well as in various organs [5,6,7,8], accumulating and leading to systemic cytotoxic processes.

A comprehensive classification of these particles exists today, reflecting their diverse properties and biological effects. The main biological cytotoxic agents are MPs, with sizes ranging from 1 μm to 5 mm, and NPs, which are subdivided into submicron particles of 100–1000 nm and true NPs of 1–100 nm. NPs are particularly hazardous because their small sizes allow them to easily penetrate cells, triggering cascades of pathological changes. Primary MPs, intentionally manufactured in small quantities, are also distinguished from secondary MPs, which form during degradation under the influence of various factors. Furthermore, this classification of particles is also based on their shape, for example, fibers, fragments, films, granules, and spheres, as well as the polymer itself, such as polyethylene (PE), polypropylene (PP), polystyrene (PS), polyvinyl chloride (PVC), and so on. Consequently, the toxicity of MPs and NPs can be determined not only by size but also by shape and chemical composition [9,10,11,12].

At the molecular level, MPs and NPs exert their toxic effects through several critically important mechanisms that disrupt the normal functioning of cells and lead to a whole range of diseases. Thus, one of the main cytotoxic effects of these particles is the induction of oxidative stress [6,13,14], leading to pathological modification and destruction of DNA, proteins, and lipids [15], as well as damage to various intracellular organelles [16,17,18,19,20]. In addition, MPs and NPs can activate inflammatory pathways, stimulating the release of pro-inflammatory cytokines through signaling cascades, such as nuclear factor-kappa B (NF-κB) [6,21] and the Nod-like receptor pyrin domain–containing 3 inflammasome (NLRP3-inflammasome) [6,22,23], as well as disrupt autophagy [24,25], apoptosis [26,27,28,29,30], necroptosis [31,32], etc. It is worth noting the DNA damage associated with MPs and NPs, which leads to mutations and potential carcinogenicity. Ultimately, these mechanisms underlie the pathogenesis of a multitude of diseases, from cardiovascular and renal disorders to neurodegenerative and psychiatric conditions, creating a vicious cycle of pathological states that lead to systemic disturbances.

For example, in the cardiovascular system, MPs and NPs cause oxidative stress and inflammation, leading, for instance, to endothelial dysfunction, impaired lipid metabolism, and activation of hemostasis [14,33]. These processes contribute to cardiomyocyte damage [6,15,34], the development of hypertrophy [34,35] and fibrosis [26,36], as well as an increased risk of atherosclerosis and thrombosis [37,38,39]. In the nervous system, MPs and NPs overcome the blood–brain barrier [5,40], causing neuroinflammation [4,41,42], aggregation of pathological proteins [16,43,44], and gut dysbiosis [5,42], which influences psychiatric disorders, such as depression [45,46,47,48], anxiety [47,48,49], bipolar disorder [50], etc., via the gut–brain axis. In the reproductive system, they disrupt hormonal balance and fertility, causing, for instance, ovarian cell apoptosis [51,52]. In the kidneys, MPs and NPs provoke inflammation and fibrosis [25,53,54,55], while in the gastrointestinal tract, they contribute to dysbiosis [56,57,58,59], impaired intestinal barrier function [53,54], and the development of metabolic disorders such as non-alcoholic fatty liver disease (NAFLD) [56,60,61].

Our research group has extensive experience in studying the molecular and cellular mechanisms underlying cardiovascular diseases [62,63,64], neurotrauma [65,66,67,68,69,70,71], renal pathologies [72,73], and other conditions. Additionally, our previous studies demonstrated that polymer coatings of slow-release fertilizer granules effectively adsorb heavy metal ions but contribute to microplastic accumulation in soil [74]. Furthermore, it was established that the sorption capacity of polystyrene particles for rhodamine B is determined by the structure of their surface layer, which can be modified, for example, by N,N′-dimethylformamide, rather than by particle shape [75].

Thus, the aim of this review is a comprehensive analysis of the mechanisms of toxic effects of MPs and NPs on various human body systems, including the cardiovascular, nervous, reproductive, urinary, and digestive systems. The review aims to systematize modern scientific data on the molecular and cellular mechanisms underlying the pathological changes caused by MPs and NPs, such as oxidative stress, inflammation, mitochondrial dysfunction, apoptosis, autophagy, and other processes. Particular attention is paid to organ-specific effects, including disorders of lipid metabolism and hemostasis in the cardiovascular system, neurodegenerative and psychiatric disorders, and reproductive toxicity, as well as kidney and gastrointestinal tract damage. The review examines the pathways of penetration, accumulation, and transport of MPs and NPs, as well as their influence on the enteric nervous system and the gut–brain axis. In addition, the review evaluates the prospects for drug therapy aimed at mitigating the consequences of MPs and NPs exposure, including antioxidant and immunomodulatory approaches. The work also aims to identify gaps in existing research and determine priority areas for further study.

## 2. Methods

This extensive scoping review analysis was conducted in accordance with the recommendations of the Preferred Reporting Items for Systematic reviews and Meta-Analyses extension for Scoping Reviews (PRISMA-ScR) [76]. The Technical Expert Group (TEG) included 6 highly qualified specialists, among whom were 2 molecular biologists (CN, SR), chemist (VI), and 3 clinicians (VC, MG, AD). The task of the TEG was a comprehensive study of the molecular and cellular mechanisms of the toxic effects of MPs and NPs on key human body systems, including the cardiovascular, nervous, reproductive, urinary, and digestive systems. The group was engaged in searching for scientific publications and assessing their reliability, as well as conceptualizing and integrating the collected data.

### 2.1. Source Search

The literature search was performed by the TEG in the PubMed, Web of Science, and Scopus databases. To ensure the greatest coverage of relevant materials, the search strategy did not include restrictions on publication date, language, or access to full texts. To optimize the process, keywords, their synonyms, and associated concepts were used.

Boolean operators (“AND”, “OR”, “NOT”) were used to form precise queries that allowed the selection of the most suitable sources (Table 1, Supplementary Materials Table S1). More detailed information on the search methodology is available in the additional materials.

**Figure 1:**
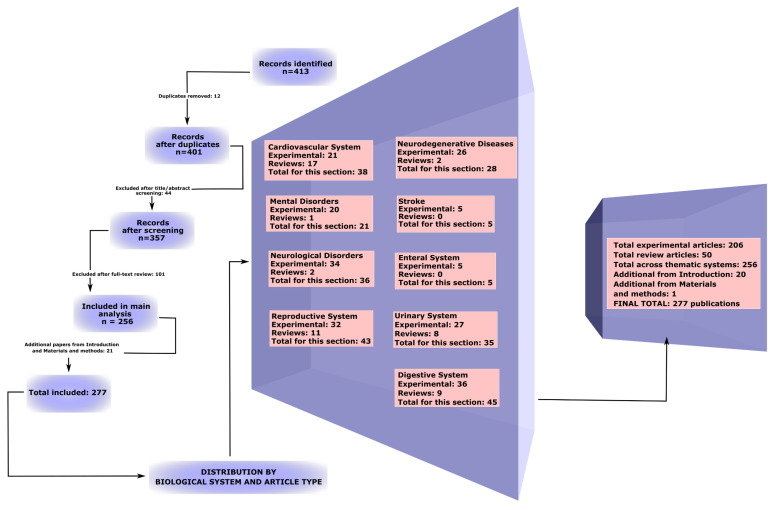
Expanded PRISMA-ScR flowchart illustrating the process of study identification, screening, eligibility assessment, and inclusion in the review, with details by publication type in the relevant sections.

During the literature search in the PubMed, Web of Science, and Scopus databases, 413 publications were identified. After removing 12 duplicates, 401 records remained. At the screening stage based on titles and abstracts, 44 studies were excluded, and following full-text evaluation, 101 publications were removed. A total of 256 scientific papers were included in the main qualitative analysis. Additionally, 20 studies were incorporated into the introduction and 1 study was from Section 2, but neither was used in the subsequent main sections. Although these studies fully complied with the PRISMA criteria, they were not directly related to the central theme of the work and were included in the introduction to clarify terminology, describe methodology, provide general scientific context, and reinforce the competence of the author team. The detailed selection process is presented in the extended PRISMA-ScR flow diagram (Figure 1, Supplementary Materials Table S2).

### 2.2. Study Quality Assessment

At the selection stage, all authors independently reviewed the titles and abstracts of the sources from the databases. If at least one of them considered the publication suitable for inclusion, the others conducted an in-depth analysis of it. The process was supervised by the last author (SR), who resolved any disagreements among co-authors. In case of disagreements, the final decision on the inclusion or exclusion of the material was made by the first (VC), second (MG), or last author (SR).

For inclusion in the review, studies were required to meet the following requirements:

1. Full transparency of the presented results.
2. Correct statistical analysis of the collected data.
3. Sufficient sample representativeness and justified selection of research materials and methods.
4. Adequate interpretation of the obtained data.

The review included both original and review articles, encompassing studies conducted on humans, animals, and cell lines. The primary focus was on original peer-reviewed publications in journals (Figure 1, Supplementary Materials Table S2).

## 3. Results

### 3.1. Cardiovascular Diseases

Available data indicate that the main mechanisms underlying cardiovascular toxicity caused by solid plastic particles include oxidative stress, inflammation, apoptosis, pyroptosis, and the interaction between solid plastic particles and various cellular organoids, such as mitochondria [77].

#### 3.1.1. Lipid Metabolism Disorders

**Figure 2:**
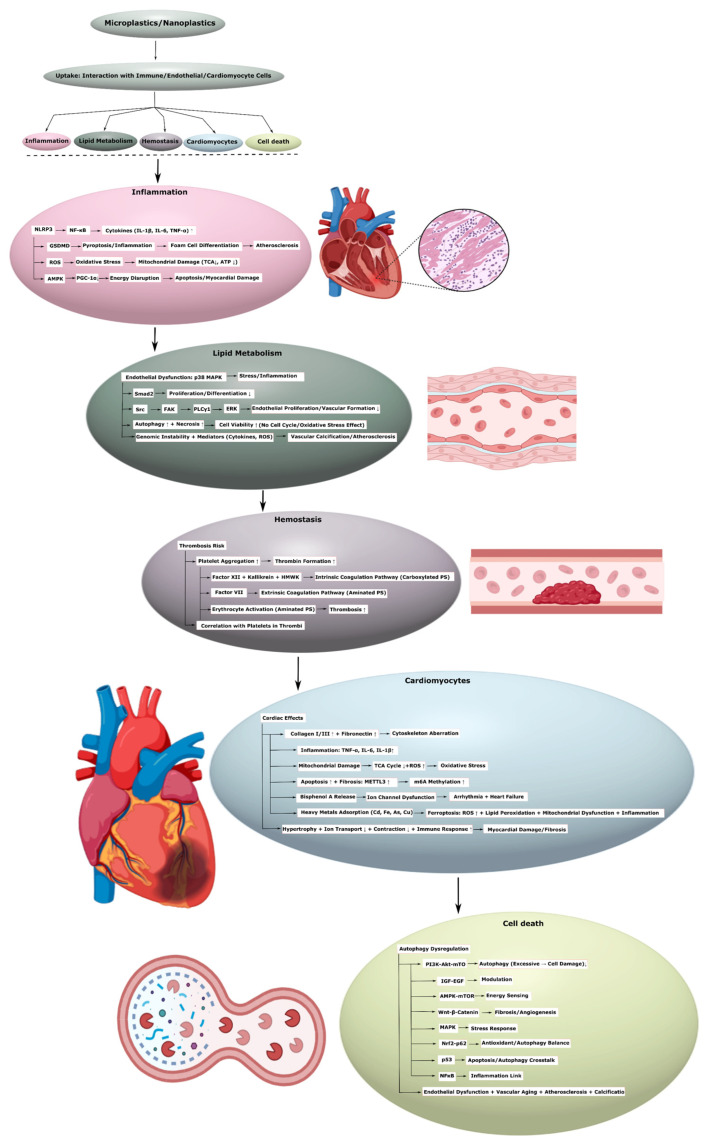
Conceptual diagram of the molecular-cellular mechanisms of microplastics and nanoplastics in the pathogenesis of cardiovascular diseases. The diagram illustrates the uptake of particles by immune, endothelial, and cardiomyocyte cells, triggering pathways of inflammation (NLRP3, NF-κB, IL-1β, IL-6, TNF-α), lipid metabolism disorders (p38 MAPK, Smad2, Src, FAK, PLCγ1, ERK), hemostasis dysregulation (Factor XII, Kallikrein, HMWK, Factor VII), cardiomyocyte damage (METTL3, m6A, ROS), and cell death (PI3K-Akt-mTOR, AMPK, Wnt-β-Catenin, Nrf2-p62, p53). Arrows (→) indicate process continuation, ↑ denotes increase, and ↓ denotes decrease. Colored blocks summarize processes related to inflammation, oxidative stress, lipid metabolism, hemostasis, cardiomyocytes, and autophagy. As a result of micro/nanoplastic-induced cardiotoxicity, atherosclerosis, thrombosis, fibrosis, arrhythmia, and heart failure may develop. Based on in vitro, in vivo, and clinical studies. Abbreviation Key: NLRP3—NOD-like receptor protein 3, NF-κB—Nuclear factor kappa B, IL-1β—Interleukin-1β, IL-6—Interleukin-6, TNF-α—Tumor necrosis factor-α, GSDMD—Gasdermin D, p38 MAPK—p38 Mitogen-activated protein kinase, Smad2—Mothers against decapentaplegic homolog 2, Src—Src kinase, FAK—Focal adhesion kinase, PLCγ1—Phospholipase C gamma 1, ERK—Extracellular signal-regulated kinase, Factor XII—Coagulation factor XII, Kallikrein—Kallikrein, HMWK—High-molecular-weight kininogen, Factor VII—Coagulation factor VII, METTL3—Methyltransferase-like protein 3, m6A—N6-methyladenosine, ROS—Reactive oxygen species, TCA—Tricarboxylic acid cycle, ATP—Adenosine triphosphate, PI3K—Phosphoinositide 3-kinase, Akt—Protein kinase B, mTOR—Mammalian target of rapamycin, AMPK—AMP-activated protein kinase, Wnt—Wingless-related integration site, β-Catenin—β-Catenin, Nrf2—Nuclear factor erythroid 2-related factor 2, p62—Sequestosome 1, p53—Tumor protein 53.

Chronic inflammation and lipid metabolism disorders play an important role in the onset and development of cardiovascular diseases. Recent data indicate the influence of MNPs on the immune system, with an emphasis on the effect on the cardiovascular system [33]. In vitro MNPs enhance the inflammatory response by activating the NLRP3 inflammasome, as observed in THP-1 cells [22]. Animal models reveal that PS induces cardiotoxicity [6] in chickens through the NF-κB-NLRP3-GSDMD and AMPK-PGC-1α axes, triggering oxidative stress, myocardial apoptosis, inflammation, and disruption of mitochondrial and energy metabolism [78]. Furthermore, NPs promote the release of pro-inflammatory cytokines in human monocytes and monocyte-derived dendritic cells [79]. Moreover, polystyrene NPs also contribute to the development of atherosclerosis by influencing mitochondria-mediated oxidative stress, promoting foam cell differentiation, and negatively affecting lipid metabolism [13]. In addition, exposure to polystyrene microplastic particles disrupts lipid metabolism and enhances oxidative stress and inflammation in the cardiovascular system of rats, which may contribute to the development of cardiac metabolic diseases (Figure 2, Table 2, Supplementary Materials Table S3) [14].

These mechanisms not only contribute to foam cell formation but also trigger inflammation, influencing the function of endothelial cells [80]. In vitro, PS MPs significantly impair the viability and functionality of human umbilical vein endothelial cells, with the effects depending on size and dose [81]. Smaller PS MPs, especially sized 0.5 and 1 μm, exhibit increased toxicity. These microparticles disrupted critical pathways involving proteins such as the mitogen-activated protein kinase p38, an integral part of stress and inflammatory responses, and Mother Against Decapentaplegic Homolog 2 (Smad2), necessary for cell proliferation, differentiation, and development. Additional affected proteins included the proto-oncogene tyrosine-protein kinase Src, focal adhesion kinase (FAK), phospholipase C gamma 1 (PLCγ1), and extracellular signal-regulated kinase (ERK), which are crucial for endothelial cell proliferation and vascular structure formation. Polystyrene microplastic particles also induce autophagy and necrosis, reducing cell viability without significantly affecting cell cycle progression or the severity of oxidative stress (Figure 2, Table 2, Supplementary Materials Table S3). This complex inhibition suggests that polystyrene microplastics disrupt important processes underlying endothelial health and vital for vascular development and repair [81].

A prospective, multicenter, observational study involving 304 patients who underwent carotid endarterectomy for asymptomatic carotid artery disease identified the role of dispersed plastic as a potential risk factor for cardiovascular diseases. The results demonstrate that patients with detection of PE and PVC in carotid artery plaques had an increased risk of the composite endpoint of myocardial infarction, stroke, or death from any cause during 34 months of follow-up [82]. Patients with vascular calcification, which is a risk factor for poor cardiovascular prognosis, had a higher level of plastic particles in their feces than patients without vascular calcification (Figure 2, Table 2, Supplementary Materials Table S3) [83]. Moreover, the thoracic aortic calcification scores significantly correlated with the content of plastic particles in the feces. Since fecal excretion is an important mechanism for the elimination of plastic particles, their content in feces can potentially be used as a measure of plastic particle exposure in assessing the risk of cardiovascular diseases [84].

The results suggest that exposure to MNPs induces inflammatory reactions leading to vascular endothelial dysfunction, by activating inflammatory cells, promoting genomic instability, and stimulating the release of cytokines and oxidative stress-associated mediators. They can also affect lipid metabolism and contribute to the development of atherosclerosis.

#### 3.1.2. Hemostasis System Disorders

Thrombosis is considered a significant risk factor for cardiovascular diseases associated with the occlusion of blood vessels [37]. Since studies data indicate that MNPs, especially MPs, are detected in human blood thrombi and can accumulate in arteries, researchers believe that the risk of their exposure is underestimated. MPs and NPs disrupt hemostasis, leading to enhanced thrombin formation, thrombus formation, and an increased risk of thrombotic diseases associated with the exposure to these particles (Figure 2) [38].

Regarding the coagulation system, a data also demonstrate a significant positive correlation between the number of particles in the thrombus and the platelet count after adjustment for potential confounding factors [85]. Moreover, the relationship between MNPs, thrombosis, and the cardiovascular system strongly depends on their size and surface chemistry, which suggests that the effect of particles is closely related to their size and chemical composition of the surface. In addition, particles with a surface charge, for example, aminated MNPs, can promote platelet aggregation and thrombosis by interacting with receptors on the platelet surface [86]. A similar experiment conducted on a model of venous thrombosis in rats revealed that intravenous administration of polystyrene nanoparticles enhances thrombus formation, possibly due to prothrombotic activation of erythrocytes by amine-modified polystyrene (Figure 2, Table 2, Supplementary Materials Table S3) [39].

The molecular mechanisms underlying these effects differ. Carboxylated PS activates the intrinsic coagulation pathway, by serving as a platform for factor XII, kallikrein, and high-molecular-weight kininogen, whereas aminated polystyrene likely promotes coagulation via the extrinsic pathway through activation of factor VII. Non-functionalized polystyrene can activate the intrinsic coagulation pathway at low concentrations and small particle sizes, but this effect weakens at higher concentrations (Figure 2, Table 2, Supplementary Materials Table S3) [87]. These results prove that monomeric nanoparticles affect the blood coagulation system and lead to cardiovascular diseases.

#### 3.1.3. Effects on Cardiomyocytes

NPs can cause changes in cardiomyocytes at the molecular level. They include increased expression of collagen types I and III and fibronectin, expanded localization of microfilament proteins, and aberrant organization of the cytoskeleton [35]. Exposure to cardiomyocytes through inflammation is a consistent and rapid reaction to PS MP exposure, with a significant increase in inflammatory factors such as TNF-α, IL-6, and IL-1β (Figure 2), which were activated in cardiac tissues even after just one week of exposure (Table 2, Supplementary Materials Table S3) [15,34].

NP exposure also induces myocardial damage, primarily through cytotoxicity resulting from disrupted energy metabolism, particularly within the tricarboxylic acid (TCA) cycle, as a consequence of mitochondrial injury. This mitochondrial disruption emerges as the principal mechanism underlying NP-induced cardiotoxicity. Additionally, polystyrene NP promotes oxidative stress by increasing the production of reactive oxygen species (ROS), which in turn damage cellular components including lipids, proteins, and DNA. These findings suggest that metabolic dysregulation and oxidative stress are central contributors to polystyrene NP-induced cardiotoxicity [15].

Exposure to MPs and NPs causes a range of cardiac effects, including hypertrophy, inflammation, immune reactions, impaired ion transport, reduced heart contraction, and mitochondrial dysfunction, leading to heart damage (Table 2, Supplementary Materials Table S3) [34,35]. In addition, NMPs cause histopathological changes in myocardial tissues, including disrupted tissue architecture, cell edema, damaged fibers, and an increase in the number of apoptotic cells, which ultimately leads to cardiac fibrosis [26]. The consequences of inducing cardiac fibrosis are further exacerbated by a significant decrease in the activity of cardiac Na^+^/K^+^-ATPase, a condition associated with ischemic heart disease. Molecular analysis revealed abnormal increases in m6A methylation in ncRNA (non-coding RNA) in microplastic-exposed myocardial tissue, particularly implying the upregulation of methyltransferase-like protein 3 (METTL3), which is associated with cardiac fibrosis (Figure 2) [36].

Plastic-associated bisphenol A (BPA) contributes to arrhythmogenesis and heart failure. BPA, widely used in the production of polycarbonate plastics and epoxy resins, is present in many consumer goods, including food and beverage packaging, dental materials, and baby bottles. Although BPA is not a plastic, it is classified as an endocrine-disrupting environmental compound and can be released during the use of plastic products, including MPs and NPs [88].

Data indicate that BPA levels are higher in patients with dilated cardiomyopathy relative to healthy individuals, suggesting a potential relationship between BPA exposure and cardiovascular disease [89]. The association of BPA with the development of arrhythmias is possibly due to its effect on ion channel function and altered electrical activity of the heart. This arrhythmogenic potential is consistent with the association of BPA with heart failure—a frequent outcome of the progression of dilated cardiomyopathy (Figure 2, Table 2, Supplementary Materials Table S3) [90,91].

Multiple studies show that MPs can act as carriers of heavy metals, in both aquatic and soil environments [92]. Their adsorption potential is largely determined by characteristics such as specific surface area, porosity, and morphology which govern their interactions at the molecular level. The metal-binding capacity is further enhanced in water with high chemical and biological oxygen demand, such as urban wastewater and irrigation water [93]. Heavy metals such as cadmium, iron, arsenic, and copper are known to cause ferroptosis—a regulated form of cell death characterized by oxidative stress, mitochondrial dysfunction, lipid peroxidation, and inflammation (Figure 2) [94]. This process can increase health risks, including effects on the cardiovascular system.

Another mechanism potentially playing an important role in the development of cardiovascular diseases is autophagy. Normally, autophagy is an evolutionarily conserved lysosomal pathway that is necessary for the degradation of damaged organelles and harmful protein aggregates, thereby preserving cellular homeostasis and viability. Conversely, deregulation of autophagy can accelerate cell damage and death, thereby contributing to pathological cascades, including endothelial dysfunction, vascular aging, and cardiovascular pathologies, including atherosclerosis and vascular calcification. Autophagy also plays a role in angiogenesis and in the paracrine regulation of vasoactive substances from the endothelium [24]. Crucial signaling pathways involved in the reciprocal modulation between autophagy and cardiovascular diseases include the PI3K/Akt/mTOR, IGF–EGF, AMPK/mTOR, Wnt/β-catenin, MAPK, Nrf2/p62, p53, and NF-κB pathways (Figure 2) [95].

However, to date, only a limited number of experiments have addressed the role of autophagy in MNP-induced effects on the cardiovascular system (Table 2, Supplementary Materials Table S3) [96,97]. This highlights the need for further research to clarify the mechanisms involved and to determine the potential implications for human health.

#### 3.1.4. Prospects for Drug Therapy for Microplastic and Nanoplastic-Related Cardiovascular Diseases

Although research on potential targets for drug therapy for MNP-induced cardiovascular diseases is in the early stages, several possible approaches can be suggested. MNPs can trigger inflammatory reactions due to their size and large surface area, interacting with cells and tissues. They can enhance systemic inflammation by activating immune cells [98]. Anti-inflammatory drugs, including inhibitors of inflammatory pathways and immune modulators, may be promising. MNPs activate oxidative stress, as evidenced by the premature senescence of endothelial cells upon exposure to polystyrene NPs [99]. Antioxidants, such as N-acetylcysteine (NAC) and resveratrol, can modulate this effect. Due to MNP’s influence on the hemostasis system, causing prothrombotic states, the study of antiplatelet agents and anticoagulants is of interest (Table 2, Supplementary Materials Table S3) [100]. A positive effect on endothelial function may be mediated by the use of drugs such as nitrates, statins, and angiotensin-converting enzyme inhibitors. Thus, exploring targets for drug therapy for MNP-induced cardiovascular diseases represents a promising area of research that requires further investigation.

**Table 2:** Experimental studies on cardiovascular toxicity of microplastics and nanoplastics: models, particle types, concentrations, and key effects (n = 21 primary sources). Arrows (→) indicate process continuation, ↑ denotes increase, and ↓ denotes decrease. Abbreviations: MPs—microplastics; NPs—nanoplastics; PS—polystyrene; PVC—polyvinyl chloride; PE—polyethylene; PET—polyethylene terephthalate; PES—polyethersulfone; PAN—polyacrylonitrile; PA6—polyamide 6; BPA—bisphenol A; HUVEC—human umbilical vein endothelial cells; THP-1—human monocytic cell line; COs—cardiac organoids; ISO—isoproterenol; NAC—N-acetylcysteine; NF-κB—nuclear factor kappa B; NLRP3—NOD-like receptor protein 3; GSDMD—gasdermin D; AMPK—AMP-activated protein kinase; PGC-1α—peroxisome proliferator-activated receptor gamma coactivator 1-alpha; VEGF—vascular endothelial growth factor; ERβ—estrogen receptor beta; SR—sarcoplasmic reticulum; BMI—body mass index; LV-left ventricle; SERCA2a—sarco/endoplasmic reticulum Ca^2+^-ATPase 2a; NCX1—Na^+^/Ca^2+^ exchanger 1; PLB—phospholamban; CASQ2—calsequestrin 2; DNMT3a—DNA methyltransferase 3a; HFD—high-fat diet; PCAEC—porcine coronary artery endothelial cells; SD—Sprague-Dawley; HR—hazard ratio; TCA—tricarboxylic acid cycle; m6A—N^6^-methyladenosine; METTL3—methyltransferase-like 3; IL—interleukin; TNF-α—tumor necrosis factor alpha; MTT—3-(4,5-dimethylthiazol-2-yl)-2,5-diphenyltetrazolium bromide; LC3—microtubule-associated protein 1A/1B-light chain 3; pERK—phosphorylated extracellular signal-regulated kinase; p-p38—phosphorylated p38 mitogen-activated protein kinase.

№	Experimental Model	Type and Source of MPs/NPs	Size/Concentrations/Duration	Results/Observations	Reference
1.	Macrophages (murine + human, in vitro)	S-NP (sulfate-PS) F-NP (Fluoresbrite^®^ YG-PS), virgin	0.20 µm, 100 µg/mL, 24–48 h	Disruption of lipid metabolism, mitochondrial oxidative stress, foam cell differentiation, contribution to atherosclerosis	[13]
2.	Rats (in vivo, oral)	PS-MP, virgin	0.5, 5, 50 mg/kg/day, 90 days	Lipid metabolism disruption, oxidative stress, inflammation in CVS	[14]
3.	THP-1 cells (human monocytes, in vitro)	PS-NH_2_ (50 nm), PS (50 nm), PVC (235 nm), PE (611 nm), PET (16 nm + 5.7 µm), PES, PAN, PA6 (fibers ~10–27 µm), virgin	50 µg/cm^2^ (~60–100 µg/mL in 1 mL)	NLRP3 inflammasome activation, ↑ IL-1β, IL-18, inflammation	[22]
4.	Mice (in vivo, inhalation)	PS-NP, virgin	16–100 mg/day, 1, 4, 12 weeks	Myocardial edema, apoptosis, fibrosis; ↓ Na^+^-K^+^-ATPase; ↑ m6A, ↑ METTL3	[26]
5.	Mice (in vivo, oral) + cardiac organoids (in vitro)	PS-MP, virgin	COs: 0.025, 0.25, 2.5 µg/mL; Mice: 25–50 µg/injection, 4 weeks COs: 72 h (+ISO 50 µM 48 h)	↑ TNF-α, IL-6, IL-1β; myocardial damage, mitochondrial dysfunction, oxidative stress, TCA cycle disruptions	[34]
6.	Neonatal rat cardiomyocytes (in vitro, synchronized)	PS-NP, virgin	60 min	Impaired collective contractility, cytoskeletal changes, ↑ collagen I/III, fibronectin	[35]
7.	In vitro (human plasma/erythrocytes) + in vivo (SD rats, 250–400 g)	Aminated PS-NP PS-MP, virgin	In vitro: 0.01–1 µg/mL, 3 h In vivo: 0.1 mg/kg, 3 h → thrombosis	Enhanced thrombogenesis, prothrombotic erythrocyte activation Coagulation activation (intrinsic/extrinsic pathways), ↑ thrombosis, platelet aggregation	[39]
8.	Chickens (in vivo, oral) + primary embryonic cardiomyocytes	PS-MP, virgin	5 µm, 1 mg/L (L), 10 mg/L (M), 100 mg/L (H) Cells: 0.01–2.5 mg/mL (+NAC 0.5 mM), 42 days (in vivo) 4–48 h (in vitro)	Cardiotoxicity: ↑ NF-κB-NLRP3-GSDMD, ↓ AMPK-PGC-1α; oxidative stress, apoptosis, inflammation, mitochondrial dysfunction	[78]
9.	Human monocytes and dendritic cells (in vitro)	PS (irregular/spherical, 50–310 nm) PMMA, PVC (irregular, polydisperse)	30–300 particles/cell (~0.1–10 µg/mL by mass), 24 h	↑ TNF-α, IL-6, IL-1β, immune response activation	[79]
10.	HUVEC (+HepG2, 3T3-L1, RAW264.7)	PS-MP, virgin	0–100 µg/mL (0.5, 1, 2, 5 µm), 24, 48, 72 h	↓ Viability (MTT)—only HUVEC, 0.5 and 1 µm > 5 µm (48–72 h) Other cells: no effect ↓ Tube formation (VEGF suppression, short-term) ↑ Autophagy/necrosis (LC3 ↑, pERK/p-p38 ↑, long-term) Angiogenesis impairment	[81]
11.	Patients (n-304, carotid endarterectomy, 34-month follow-up)	PE, PVC (in plaques)	57% plaques, ~20 particles/plaque	HR 4.2 for MI, stroke, death	[82]
12.	Patients with vascular calcification (n-47)	Various polymers (in feces)	↑ in calcification group	Correlation of fecal plastics with calcification; exposure marker	[83]
13.	Human thrombi (n-26)	Various polymers	21–530 particles/thrombus	Positive correlation with platelets; contribution to thrombogenesis	[85]
14.	In vitro (human whole blood)	Polystyrene: aPS, cPS, nPS	50, 100, 500 nm, 25, 100, 250 µg/mL	cPS: ↑ fibrin polymerization rate, ↑ clot strength (size- and concentration-dependent); nPS: minimal effect (except 50 nm at low conc.); aPS (100 nm): effect like cPS, ↓ at 500 nm	[87]
15.	Patients with dilated cardiomyopathy	BPA (leached from plastics)	↑ in serum vs. healthy	Potential link of BPA to dilated CMP	[89]
16.	C57bl/6n mice (males and females)	Bisphenol A (BPA)—leached from plastics	0.5; 5.0 µg/kg/day lifelong; 200 µg/kg/day—from GD11 to PND21	↑ Body weight, BMI, obesity (5.0 µg/kg/day); concentric LV remodeling (males); ↑ diastolic BP (females); sex dimorphism: ↑ Ca^2+^ mobility (males), ↓ (females); changes in SERCA2a, NCX1, PLB, p-PLB, CASQ2; epigenetics: ↑ DNMT3a, sex-specific CpG methylation in CASQ2	[90]
17.	Female rat myocytes (in vitro)	Bisphenol A (BPA)—endocrine disruptor from plastics	10^−12^–10^−6^ M; acute exposure	Non-monotonic dose–response: ↑ arrhythmias, Ca^2+^ transient changes; monotonic: ↑ SR Ca^2+^ release/uptake, ↓ L-type Ca^2+^ current; via ERβ	[91]
18.	HUVEC (in vitro)	PS-NP, virgin	25 µg/mL (100, 500 nm), 48 h	↑ Autophagy (↑ LC3-II, Beclin-1; ↓ p62), size-dependent; contribution to endothelial dysfunction	[97]
19.	In silico (docking), in vitro (BV2), in vivo (HFD-obese mice)	fPS-MP (fluorescent polystyrene), 1 µm	Dose-dependent (in vitro); oral (in vivo)	Docking: styrene > ethylene/propylene in macrophage binding ↑ fPS-MP binding to microglia (dose-dependent) In vivo: fPS-MP in blood + immune cells, ↑ insulin resistance, ↑ systemic inflammation Brain accumulation, ↑ hypothalamic microglia activation MPs exacerbate inflammation and metabolism in obesity	[98]
20.	PCAEC (in vitro)	PS-NP, virgin	10 µg/mL	Premature endothelial senescence, oxidative stress, inflammation	[99]
21.	Mice (in vivo, i.v.)	PS, PS-NH_2_, PS-COOH, virgin	80 nm, 4 mg/kg (100 µL × 1 mg/mL), 24 h	Endothelial injury, coagulopathy, thrombosis	[100]

### 3.2. Neurodegenerative Diseases

MNPs pose an increasing risk to human health, with potential involvement in both neurological and psychiatric disorders. Their capacity to penetrate biological barriers, including the blood–brain barrier and intestinal barrier, allows them to accumulate in brain tissue, inducing a spectrum of neurotoxic effects, including neuroinflammation, oxidative stress, mitochondrial dysfunction, disruption of neurotransmitter systems- that are closely associated with the development of neurodegenerative diseases (e.g., Parkinson’s disease, Alzheimer’s disease, amyotrophic lateral sclerosis, ischemic stroke) and psychiatric conditions (e.g., depression, anxiety, schizophrenia, bipolar disorder).

#### 3.2.1. Parkinson’s Disease

There is increasing evidence today that the neurotoxic effects of MPs and NPs may underlie the pathogenesis of PD. As demonstrated by experiments on animal and cellular models, these particles contribute to the degeneration of dopaminergic neurons, α-synuclein aggregation, and, as a consequence, the onset of motor disturbances characteristic of PD.

**Figure 3:**
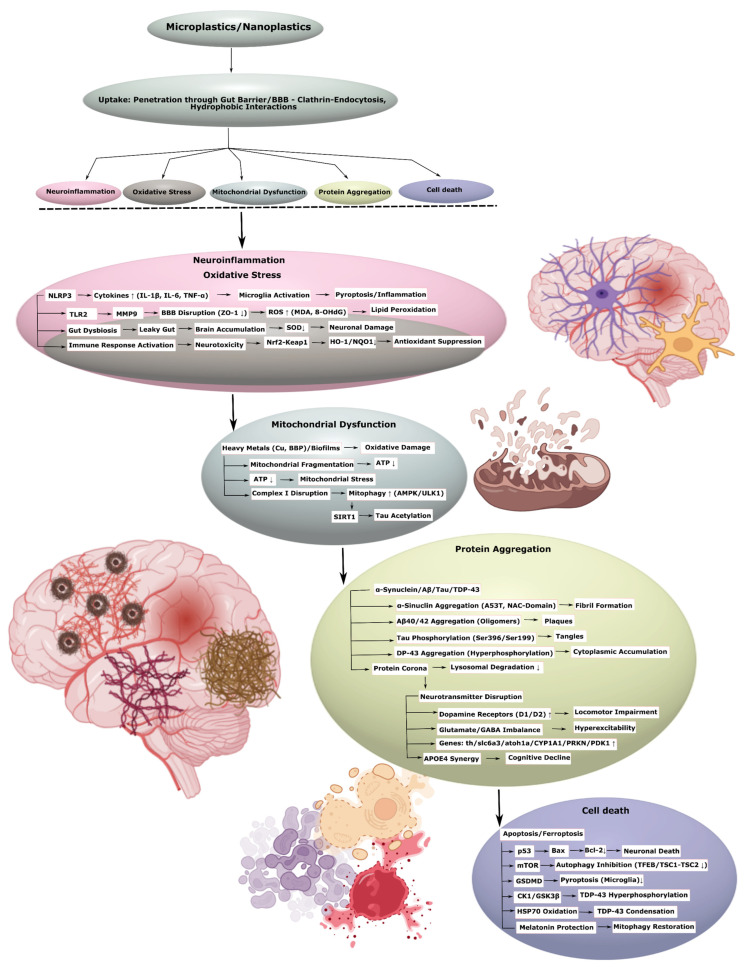
Conceptual diagram of the molecular-cellular mechanisms of microplastics and nanoplastics in the pathogenesis of neurodegenerative diseases. The diagram illustrates particle uptake through the intestinal barrier and blood–brain barrier (BBB) via clathrin-mediated endocytosis and hydrophobic interactions, triggering pathways of neuroinflammation (NLRP3, TLR2, MMP9, IL-1β, IL-6, TNF-α), oxidative stress (ROS, MDA, 8-OHdG, SOD, Nrf2-Keap1), mitochondrial dysfunction (AMPK, ULK1, SIRT1), protein aggregation (α-Synuclein, Aβ, Tau, TDP-43), and dysregulation of autophagy/pyroptosis (p53, Bax, Bcl-2, mTOR, GSDMD). Arrows (→) indicate process continuation, ↑ denotes increase, and ↓ denotes decrease. Colored blocks summarize processes related to neuroinflammation, oxidative stress, mitochondrial dysfunction, protein aggregation, and cell death. As a result of micro/nanoplastic-induced pathological cascades, neurotoxicity develops, leading to protein aggregation, cell death, mitochondrial dysfunction, impaired neurotransmission, cognitive deficits, and neurodegenerative diseases. Based on in vitro and in vivo studies. Abbreviation Key: BBB—blood–brain barrier, NLRP3—NOD-like receptor protein 3, IL-1β—Interleukin-1β, IL-6—Interleukin-6, TNF-α—Tumor necrosis factor-α, TLR2—Toll-like receptor 2, MMP9—Matrix metalloproteinase 9, ZO-1—Zonula occludens-1, ROS—Reactive oxygen species, MDA—Malondialdehyde, 8-OHdG—8-Hydroxy-2′-deoxyguanosine, SOD—Superoxide dismutase, Nrf2—Nuclear factor erythroid 2-related factor 2, Keap1—Kelch-like ECH-associated protein 1, HO-1—Heme oxygenase-1, NQO1—NAD(P)H quinone dehydrogenase 1, ATP—Adenosine triphosphate, AMPK—AMP-activated protein kinase, ULK1—Unc-51-like autophagy-activating kinase 1, SIRT1—Sirtuin 1, α-Synuclein—α-Synuclein, Aβ—Amyloid-β, Tau—Tau protein, TDP-43—TAR DNA-binding protein 43, A53T—A53T mutation in α-Synuclein, NAC-Domain—Non-amyloid component domain, Ser396/Ser199—Phosphorylation sites Ser396/Ser199 on Tau protein, D1/D2—Dopamine receptors D1/D2, GABA—Gamma-aminobutyric acid, th—Tyrosine hydroxylase, slc6a3—Dopamine transporter, atoh1a—Atonal homolog 1a, CYP1A1—Cytochrome P450 1A1, PRKN—Parkin, PDK1—Pyruvate dehydrogenase kinase 1, APOE4—Apolipoprotein E4, p53—Tumor protein 53, Bax—BCL2-associated X protein, Bcl-2—B-cell lymphoma 2, mTOR—Mammalian target of rapamycin, TFEB—Transcription factor EB, TSC1-TSC2—Tuberous sclerosis complex 1-2, GSDMD—Gasdermin D, CK1—Casein kinase 1, GSK3β—Glycogen synthase kinase 3β, HSP70—Heat shock protein 70.

MPs and NPs, including polystyrene nanoplastics within the 25–100 nm size range, are capable of traversing the intestinal and blood–brain barriers, triggering neurotoxic effects associated with Parkinson’s disease. In mice and *Caenorhabditis elegans*, even low concentrations of MPs accelerated the degeneration of dopaminergic neurons in the substantia nigra, causing locomotor disturbances. These neurotoxic effects appear to be mediated by systemic disruption, involving the degradation of the intestinal barrier, microbiota dysbiosis, and activation of the immune response, accompanied by excessive production of ROS and mitochondrial stress [5]. Interestingly, polystyrene NPs accumulate in the digestive tract, causing “leaky gut” syndrome and mitochondrial fragmentation, which ultimately correlates with the accumulation of α-synuclein aggregates in human cells and in *C. elegans* [101]. Also, oral administration of polystyrene NPs led to the aggregation of A53T-mutated α-synuclein against a background of increasing inflammatory response, consisting of the expression of pro-inflammatory cytokines and oxidative stress with degradation of mitochondrial and lysosomal membranes (Figure 3, Table 3, Supplementary Materials Table S4) [16].

Neurotoxic mechanisms of MPs and NPs extend to programmed cell death pathways, including autophagy impairment and pyroptosis induction. Polystyrene NPs disrupt the BBB and promote dopaminergic neuron loss, aggravating cognitive impairment. These effects occur through enhanced mTOR pathway signaling, suppression of TFEB, and degradation of the TSC1-TSC2 complex, resulting in autophagy inhibition and pyroptosis activation (Figure 3) [40]. In turn, spectroscopic analysis confirms that polystyrene NPs effectively promote the formation of amyloidogenic α-synuclein oligomers due to changes in its secondary structure and because of their small sizes [43]. Anionic NPs can accelerate the formation of α-synuclein fibrils through interaction with NAC domains (Figure 3), penetrating neurons by clathrin-dependent endocytosis and slowing lysosomal degradation, inducing neurotoxic effects in the substantia nigra (Table 3, Supplementary Materials Table S4) [102].

Mass spectrometry analysis reveals that polystyrene NPs modulate the oligomerization of NACore, an α-synuclein surrogate, enhancing toxicity in microglia through hydrophobic interactions, which is also observed in *Danio rerio* models [103]. In SH-SY5Y cells, polystyrene NPs disrupt the function of mitochondrial complex I, reducing ATP production and causing excessive mitophagy via the AMPK/ULK1 pathway. Melatonin, in turn, mitigates these effects, restoring mitophagy regulation and improving motor function in mice (Figure 3, Table 3, Supplementary Materials Table S4) [17].

Unlike other nanoparticles, such as graphene oxide, polystyrene NPs can cause neuroexcitation in *Danio rerio* through oxidative stress and inhibition of the expression of the atoh1a gene, altering neuroprotein profiles [104]. Furthermore, MP impurities, such as benzyl butyl phthalate (BBP), can interact with the PRKN and PDK1 proteins, enhancing neurodegeneration [105]. Also, biofilms on the MP surface adsorb heavy metals, such as copper, disrupting cellular homeostasis and inducing mitochondrial dysfunction, which is a key factor in PD pathogenesis [18]. Chronic exposure to MPs and copper in *Danio rerio* increased the expression of the th and slc6a3 genes, associated with the dopaminergic system, and reduced locomotor activity [106]. Polystyrene NPs also dose-dependently increased the expression of dopamine receptors D1 and D2 in cortical neurons, indicating dysregulation of the dopaminergic system (Figure 3, Table 3, Supplementary Materials Table S4) [107].

Collectively, these data underscore the role of MPs and NPs as environmental risk factors for Parkinson’s disease, contributing to neurodegeneration through α-synuclein aggregation, mitochondrial dysfunction, neuroinflammation, and disruption of biological barriers.

#### 3.2.2. Alzheimer’s Disease

MPs and NPs can influence another neurodegenerative disease, AD, as indicated by many scientific data. These particles can act as active participants in this pathological process, stimulating the formation of amyloid-β (Aβ) plaques and tau tangles, and also disrupting the fragile balance in the gut–liver–brain axis, which ultimately leads to the progression of AD-associated cognitive impairments (Figure 3).

MPs, including PP, PVC, PE, and PS, are found in human cerebrospinal fluid (CSF), with their concentrations correlating with the frequency of bottled water consumption and increased BBB permeability. In patients with amyloid-positive status, the levels of polyethylene and PVC in the CSF are higher than in amyloid-negative individuals and show an inverse correlation with Aβ42 levels and MMSE scale scores. PE is also linked to the acceleration of cognitive decline, where Aβ42 acts as a mediator [108]. Polystyrene NPs (PS-NPs) have also been shown to enhance cognitive impairment in AD mice by inducing microglial pyroptosis and neuroinflammation (Figure 3, Table 3, Supplementary Materials Table S4) [4].

In BV2 and RAW264.7 cell lines, GSDMD inhibition suppressed pyroptosis. PS-NPs were also shown to enhance AD symptoms in wild-type mice and APP/PS1 transgenic models by activating microglia and causing hippocampal neuron death. These changes were accompanied by systemic disturbances, such as hepatic steatosis, inflammation, and microbiota dysbiosis [42]. The synergistic interaction of MPs with the APOE4 allele leads to sex-dependent changes in locomotion and memory in APOE4 mice, accompanied by modification of astrocytic and microglial markers in the brain and increased CYP1A1expression in the liver [109]. In APP/PS1 and BV2 models, environmentally relevant doses of PS-NPs exacerbate cognitive deficits and Aβ-plaque deposition by disrupting lysosomal function and inducing microglial pyroptosis, reducing their phagocytic capacity. Melatonin restores microglial function, reduces plaques, and improves cognitive scores (Table 3, Supplementary Materials Table S4) [110].

PS-NPs in low concentrations accelerate the aggregation of Aβ40 and Aβ42, promoting the formation of toxic oligomers through hydrophobic interactions [111]. For example, amino-modified PS-NPs can penetrate the brain, activating APP and MAPT genes and disrupting BBB tight junctions occludin (ZO-1) through the TLR2/MMP9 pathway, and causing neuronal apoptosis due to iNOS/nNOS activation, SIRT1 inactivation, and tau acetylation. *Camellia pollen*, in turn, mitigated these effects, including apoptosis via the p53/Bax/Bcl-2 pathway [27]. PS-NPs also accelerate Aβ fibrillation, forming a protein corona, and disrupt Aβ uptake by microglia, depleting energy resources and damaging ABC transporters, enhancing inflammation and metabolic dysregulation (Figure 3, Table 3, Supplementary Materials Table S4) [41].

Prenatal exposure to PS-NPs in mice reduced the brain mass of the offspring, caused neuron loss, Nissl body degeneration, neurofibrillary tangle formation, and increased phosphorylated tau at ser396 and ser199 against a background of increasing Aβ [112]. Also, the biofilm forming on the MP surface and adsorbing heavy metals can lead to AD through enhanced oxidative stress and the development of mitochondrial dysfunction [18]. Chronic exposure to polyethylene MPs in rats reduced superoxide dismutase (SOD) expression and serum Aβ42 levels, while increasing markers of oxidative stress, such as MDA, 8-OHdG, in hippocampal neurons, which is linked to the accumulation of amyloid aggregates [113]. Epidemiological data confirm higher concentrations of MPs/NPs in the brains of dementia patients, especially in vascular walls and immune cells, compared to the control group [114]. In chickens, polystyrene MPs reduced the expression of BBB tight junction proteins and caused ferroptosis and apoptosis through suppression of the Nrf2-Keap1/HO-1/NQO1 pathway, promoting the accumulation of pathological proteins (Figure 3, Table 3, Supplementary Materials Table S4) [28].

Collectively, the evidence indicates that MPs and NPs act as accelerators of Alzheimer’s disease pathology, enhancing Aβ and tau aggregation, compromising microglial and BBB function, and causing systemic perturbations that drive disease progression.

#### 3.2.3. Amyotrophic Lateral Sclerosis

MPs and NPs may also be involved in the pathogenesis of ALS—a severe disease affecting motor neurons. Scientific studies conducted on cellular and animal models demonstrate that these particles provoke the pathological aggregation of the TDP-43 protein, as well as enhance chronic neuroinflammation and oxidative stress, disrupting motor neuron function.

PS-NP particles cross BBB, triggering neurotoxic effects, such as inflammation with increased pro-inflammatory cytokines, oxidative stress, mitochondrial dysfunction, and disruptions in neurotransmitter homeostasis, including glutamate and GABA. These changes promote neuronal hyperexcitability, paralleling mechanisms involved ALS. Furthermore, MPs and NPs disrupt the gut microbiota composition, amplifying pathological processes via the gut–brain axis. Exposure to MPs through food, inhalation, or dermal contact, including exposure of newborns via milk or toys-may contribute to the preclinical development of ALS [19].

In neurons derived from human induced pluripotent stem cells (iPSC), PS-NPs penetrate cells depending on their size, interacting with proteins including TDP-43, and the kinases CK1 and GSK3β, inducing TDP-43 hyperphosphorylation, causing ALS-like phenotypes with impaired neuronal morphology, worsened mitochondrial respiration, and accelerated motor neuron death [115]. PS-NPs also induce oxidative stress, contributing to TDP-43 aggregation and its accumulation in the cytoplasm. Oxidized heat shock protein 70 (HSP70) lost its ability to return TDP-43 to the nucleus, enhancing the condensation and hardening of TDP-43 in a complex with PS-NPs (Figure 3). These processes, confirmed in in silico and in vivo studies, illustrate the link between MP contamination and neurodegenerative processes characteristic of ALS (Table 3, Supplementary Materials Table S4) [44].

Taken together, these data underscore the role of MPs and NPs as factors contributing to ALS pathogenesis through oxidative stress, TDP-43 homeostasis disruption, and motor neuron dysfunction.

### 3.3. Stroke

The role of MPs and NPs in ischemic stroke attracts attention due to the ability of these particles to cause neuroinflammation, damage the BBB, and disrupt cerebral blood flow, which exacerbates neurological deficits. Studies on animal models and in vitro highlight the neurotoxic effect of MPs and NPs, revealing mechanisms that may contribute to stroke progression.

In a mouse transient middle cerebral artery occlusion (tMCAO) model, oral administration of MPs, including PE and PVC, significantly exacerbated neurological deficits. Compared to placebo, the PE and PVC groups showed reduced Garcia scale scores, increased neurological deficit, worsened motor function on the rotating rod test, and increased infarct volume on the third day after tMCAO. Polystyrene MPs did not show significant effects in this model, indicating differences in toxicity depending on the type of plastic (Table 4, Supplementary Materials Table S5) [116].

In a global cerebral ischemia model, oral administration of MPs before and after ischemia enhanced neuroinflammation, microtubule disruption, and neuronal death. MPs were shown to activate microglia, damage myelin and microtubules, and increase levels of inflammatory cytokines and phosphorylated tau (Table 4, Supplementary Materials Table S5) [117].

In the blood, MPs and NPs are phagocytized, causing cortical capillary obstruction, which leads to reduced cerebral blood flow and neurological deficits similar to thrombosis. High-resolution in vivo imaging confirms that these changes are due to the indirect effect of MPs through circulatory disruption, rather than solely their direct penetration into tissues [3]. Polystyrene NPs increase BBB permeability in mice by suppressing the expression and distribution of the ZO-1 protein in brain microvascular endothelial cells. PS-NPs reduce transendothelial resistance and induce ferroptosis through Fe^2+^ accumulation and lipid peroxidation, disrupting glutathione metabolism. The ferroptosis inhibitor ferrostatin-1 (Fer-1) restores ZO-1 expression, protects the BBB, and mitigates these changes, indicating ferroptosis as a key damage mechanism upon MP exposure (Table 4, Supplementary Materials Table S5) [118].

In chickens, exposure to polystyrene MPs caused intracerebral hemorrhage, microthrombus formation, and Purkinje cell loss. These changes were accompanied by inflammatory cell infiltration and activation of the ASC/NLRP3/GSDMD signaling pathway, inducing pyroptosis. Disrupted mitochondrial dynamics and activation of the AMPK pathway indicate mitochondrial dysfunction as a key mechanism of physical brain damage induced by MPs, which may be relevant to hemorrhagic stroke [23]. In the tMCAO mouse model, oral administration of polyethylene and polyvinyl chloride enhanced neurological deficits, reducing Garcia scale scores, increasing neurological deficit, worsening motor function, and increasing infarct volume. Polystyrene MPs did not show significant effects, indicating differences in the toxicity of plastic types (Table 4, Supplementary Materials Table S5) [116].

Collectively, these data underscore the role of MPs and NPs as factors exacerbating ischemic stroke through enhanced neuroinflammation, BBB damage, disrupted blood flow, and neuronal death.

### 3.4. Neurological Disorders

#### 3.4.1. Neurodevelopmental Toxicity

**Figure 4:**
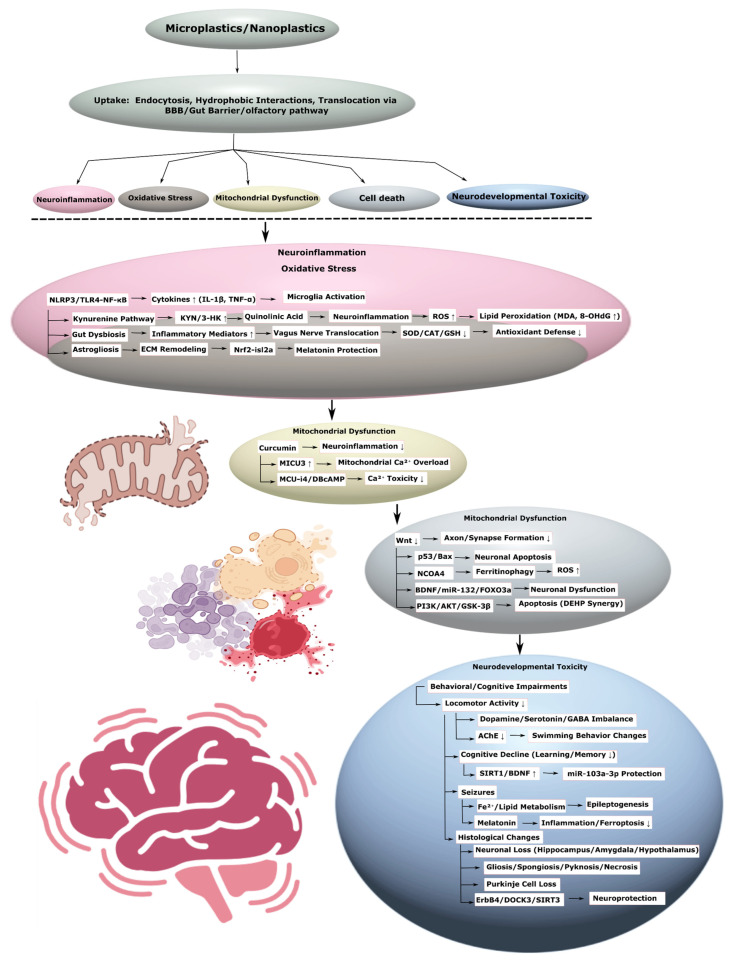
Conceptual diagram of the molecular-cellular mechanisms of microplastics and nanoplastics in neurological disorders and general neurotoxic processes. The diagram illustrates particle uptake via endocytosis, hydrophobic interactions, and translocation through the blood–brain barrier (BBB), intestinal barrier, or olfactory pathway, triggering pathways of neuroinflammation (NLRP3, TLR4-NF-κB, IL-1β, TNF-α, Kynurenine Pathway), oxidative stress (ROS, MDA, 8-OHdG, SOD, CAT, GSH, Nrf2-isl2a), mitochondrial dysfunction (MICU3, MCU-i4, DBcAMP, Wnt), apoptosis/ferroptosis (p53, Bax, NCOA4, BDNF, miR-132, FOXO3a, PI3K/AKT/GSK-3β), and neurodevelopmental toxicity (AChE, SIRT1, miR-103a-3p, Fe^2+^). Arrows (→) indicate process continuation, ↑ denotes increase, and ↓ denotes decrease. Colored blocks summarize processes related to neuroinflammation, oxidative stress, mitochondrial dysfunction, cell death, and neurodevelopment. As a result of micro/nanoplastic-induced pathological cascades, apoptosis, behavioral/cognitive impairments, seizures, and histological changes develop. Based on in vitro and in vivo studies. Abbreviation Key: BBB—blood–brain barrier, NLRP3—NOD-like receptor protein 3, TLR4—Toll-like receptor 4, NF-κB—Nuclear factor kappa B, IL-1β—Interleukin-1β, TNF-α—Tumor necrosis factor-α, KYN—Kynurenine, 3-HK—3-Hydroxykynurenine, ROS—Reactive oxygen species, MDA—Malondialdehyde, 8-OHdG—8-Hydroxy-2′-deoxyguanosine, SOD—Superoxide dismutase, CAT—Catalase, GSH—Glutathione, Nrf2—Nuclear factor erythroid 2-related factor 2, isl2a—Islet2a (transcription factor), MICU3—Mitochondrial calcium uniporter 3, MCU-i4—Mitochondrial calcium uniporter inhibitor 4, DBcAMP—Dibutyryl cyclic AMP, Wnt—Wingless-related integration site, p53—Tumor protein 53, Bax—BCL2-associated X protein, NCOA4—Nuclear receptor coactivator 4, BDNF—Brain-derived neurotrophic factor, miR-132—MicroRNA-132, FOXO3a—Forkhead box O3a, PI3K—Phosphoinositide 3-kinase, AKT—Protein kinase B, GSK-3β—Glycogen synthase kinase 3β, DEHP—Di(2-ethylhexyl)phthalate, GABA—Gamma-aminobutyric acid, AChE—Acetylcholinesterase, SIRT1—Sirtuin 1, miR-103a-3p—MicroRNA-103a-3p, Fe^2+^—Ferrous iron, ErbB4—ErbB4 receptor, DOCK3—Dedicator of cytokinesis 3, SIRT3—Sirtuin 3.

MNPs have a significant impact on neurodevelopment. Polystyrene MPs reduced swimming speed and motor neuron axon length in Danio rerio, causing apoptosis and suppression of neurodevelopmental genes [119]. Melatonin mitigated these effects through activation of the nrf2-isl2a axis. In chick embryos, polystyrene NPs caused neural tube defects through activation of autophagy and apoptosis [29]. In human neural stem cells, polystyrene NPs reduced proliferation and induced apoptosis through endocytosis, without penetrating the nucleus [30]. Polystyrene NPs sized 50 nm penetrated deeper into tissues than larger particles, reducing organoid viability in cerebral organoids and mice. Mice exposed to MPs exhibited DNA fragmentation in the hippocampus and cerebral cortex, as well as increased levels of inflammatory markers and neurotoxic metabolites, such as kynurenine (KYN) and 3-hydroxykynurenine (3-HK). RNA sequencing revealed changes in gene expression associated with neurotoxicity, indicating the activation of the kynurenine pathway, which promotes quinolinic acid accumulation and neuroinflammation [20]. In human neural stem cells, polystyrene NPs reduced proliferation and induced apoptosis through endocytosis, without penetrating the nucleus, indicating high neurodevelopmental toxicity of smaller particles [30]. Co-exposure to polystyrene MPs/NPs and silver nanoparticles in *Danio rerio* larvae caused neurodevelopmental abnormalities, including malformations, reduced locomotor activity, and delayed response. These changes were accompanied by oxidative stress, neuroinflammation, and altered expression of genes related to immunity, apoptosis, vascular, and neural development (Figure 4). Curcumin, a neuroinflammation inhibitor, mitigated these effects, especially with NP exposure, highlighting their higher toxicity compared to MPs (Table 5, Supplementary Materials Table S6) [120].

In *Danio rerio* embryos, polystyrene MPs did not affect survival or hatching, but caused changes in swimming behavior, linked to apoptosis, reduced acetylcholinesterase (AChE) activity, increased nitric oxide (NO), and neurotransmitter dysregulation, such as serotonin and dopamine. Reduced BDNF expression indicates neuronal dysfunction, confirmed by bioinformatics analysis showing high affinity of styrene for Bcl-2, p53, and BDNF proteins [121]. In *Caenorhabditis elegans*, polystyrene NPs dose-dependently caused oxidative stress, mitochondrial damage, and reduced dopamine content, leading to inhibited growth, locomotor activity, and neurodevelopment. Smaller particles showed less toxicity due to distribution and polymerization characteristics, and presenilin genes, such as *sel-12* and *hop-1*, regulated these effects, linking them to oxidative stress and mitochondrial dysfunction [122]. Polystyrene NPs reduced cell viability and differentiation in cerebral organoids by disrupting mitochondrial function. Transcriptomic analysis revealed suppression of the Wnt signaling pathway, responsible for neuronal development, and reduced expression of neuronal cadherin, which hinders axon extension and synapse formation (Figure 4, Table 5, Supplementary Materials Table S6) [123].

In neonatal rats, maternal exposure to polystyrene NPs during pregnancy and lactation induced ferroptosis in the offspring’s hippocampus, reducing cognitive ability, learning, and memory. Ferritinophagy, mediated by NCOA4 and p53, increased ROS, reducing GPX4 and GSH levels [124]. In Danio rerio, combined exposure to PS-MPs and amitriptyline did not affect development, but amitriptyline increased AChE and carboxylesterase activity, altering swimming behavior [125,126]. MPs did not modulate amitriptyline effects. In Danio rerio, PS-MPs and amitriptyline enhanced locomotor activity and schooling behavior, reducing SOD, CAT, and GSH in the eyes, with additive behavioral toxicity linked to oxidative stress (Figure 4, Table 5, Supplementary Materials Table S6) [127].

#### 3.4.2. Seizures

Long-term exposure to polystyrene MPs has been linked to seizure symptoms through mechanisms involving hippocampal inflammation and ferroptosis. Studies in humans and mice indicate that MPs disrupt iron and lipid metabolism, thereby promoting seizure activity. Treatment with melatonin mitigated these effects by suppressing inflammation and ferroptosis (Figure 4). Suggesting that MPs may play a contributory role in epileptogenesis (Table 5, Supplementary Materials Table S6) [128].

#### 3.4.3. General Neurotoxic Effects

MPs/NPs cause a wide range of neurotoxic effects through BBB disruption, oxidative stress, neuroinflammation, and apoptosis. Polyethylene MPs disrupted BBB integrity in rats, increased oxidative stress through reduced antioxidant defense and increased MDA, and lowered BDNF levels [129]. In mice, polystyrene NPs penetrated the BBB, activating microglia and inducing endothelial cell necroptosis [130]. In Danio rerio, MPs enhanced methylmercury neurotoxicity, disrupting locomotor activity and glutathione metabolism [131]. Polystyrene MPs caused neuronal toxicity, increasing lipid peroxidation (LPO), NO, hydroxyl radical, ammonia, and glutamate levels, while reducing levels of GABA, antioxidant enzymes, and mTOR (Figure 4). Lycopene (LYC) restored neurochemical balance, GABA level, and cerebral cortex structure, mitigating oxidative stress and improving lysosomal function, indicating a link between MPs and cognitive dysfunction (Table 5, Supplementary Materials Table S6) [132].

Analysis of human olfactory bulbs revealed the presence of MPs in 8 out of 15 deceased individuals, with particle sizes from 5.5 to 26.4 μm. This confirms the translocation of MPs into the brain, likely via the olfactory pathway, highlighting their potential neurotoxicity and the need to study BBB penetration mechanisms [133]. Polystyrene MPs increased BBB permeability by 15.6–27.3 times compared to control in a BBB model, causing more damage than 1.0 μm particles. Pretreatment with TNF-α enhanced absorption and toxicity, emphasizing the dependence of effects on particle size and inflammatory status [134]. In *Danio rerio*, co-exposure to polyvinyl chloride MPs and copper caused neuronal apoptosis through the BDNF/miR132/FOXO3a axis, with increased ROS, LPO, protein carbonyls, and 8-OHdG, as well as reduced dopamine, serotonin, and acetylcholine. These changes were accompanied by neuroarchitectural damage and enhanced apoptosis, especially with combined exposure [135]. Co-exposure to polystyrene NPs and di-(2-ethylhexyl) phthalate (DEHP) in mice caused neuronal apoptosis through the PI3K/AKT pathway, with disruption of mitochondrial function and increased GSK-3β expression, confirmed by pathological changes in the brain [136]. MPs and NPs disrupt the gut microbiota, causing dysbiosis, activation of inflammatory mediators, and metabolite translocation via the vagus nerve, leading to neuroinflammation, accumulation of neurofibrillary tangles, and oxidative stress, associated with neurological disorders [137]. Biodegradable plastics, such as polylactic acid (PLA), also exhibit neurotoxicity. In the gastrointestinal tract, PLA degrades into oligomeric NPs, increasing their bioavailability and causing mitochondrial calcium overload through enhanced MICU3 expression. Inhibitors of calcium influx (MCU-i4) or stimulators of its efflux (DBcAMP) reduce toxicity [138]. PS-MPs also caused a reduction in neuron count in the hippocampus, amygdala, and hypothalamus [139]. In juvenile Pomatochistus microps, polyethylene MPs modulated the sublethal toxicity of cadmium (Cd), reducing AChE activity and post-exposure predatory performance (PEPP), with an antagonistic interaction between MPs and Cd (Figure 4, Table 5, Supplementary Materials Table S6) [140].

In mice, polystyrene MPs caused cognitive impairment linked to oxidative stress, pyroptosis, apoptosis, and endoplasmic reticulum (ER) stress in the hippocampus. Intracerebroventricular administration of miR-103a-3p reduced levels of malondialdehyde, protein carbonyl, nitrite, caspase-3, caspase-1, TNFα, and NLRP3, restoring SIRT1 and BDNF expression and reducing ER stress by decreasing PERK, CHOP, GRP78, improving learning and memory [141]. In human neural stem cells and derived astrocytes, polystyrene MPs/NPs reduced astrocyte viability, causing astrogliosis. Transcriptomic analysis revealed differential expression of 1274 genes and 531 genes related to neuroinflammation, immunity, cell migration, extracellular matrix remodeling, and lipid metabolism [142]. In *Caenorhabditis elegans*, polystyrene NPs caused lethality, inhibition of growth, reproduction, and locomotor behavior, damaging GABAergic and cholinergic neurons. Toxicity was size- and dose-dependent and regulated by insulin signaling pathways [143]. In mice, intranasal administration of polystyrene NPs caused accumulation in the brain and neurotoxicity, which was reduced by ACY-1215, an HDAC6 inhibitor, by enhancing NP exocytosis from neurons [144]. In cerebral organoids, polystyrene NPs disrupted mitochondrial function, reduced cell viability and differentiation, confirming their toxicity to the human brain [145]. In carp, polystyrene MPs caused lymphocyte necroptosis in the head kidney, increasing mitochondrial ROS, calcium, and inflammatory cytokines, which was reduced by astaxanthin through regulation of the miR-25-5p/MCU axis [32]. Activation of the ErbB4 receptor by the E4A agonist in mice mitigated neuronal damage induced by polystyrene MPs, improving cognitive function, increasing DOCK3 and SIRT3 expression, and reducing neuroinflammation through the TLR4-NF-κB-NLRP3 pathway [21]. In *Danio rerio*, polystyrene MPs reduced locomotor activity and induced apoptosis, and melatonin reduced these effects through activation of the Nrf2-isl2a axis (Figure 4) [119]. In another study on *Danio rerio*, MPs enhanced visual tectum lesions upon co-exposure with ketamine and methionine, causing oxidative stress, necrosis, and edema [146]. In *Danio rerio*, co-exposure to polystyrene MPs and methyltestosterone caused neuronal damage and disruption of the “neuroactive ligand-receptor interaction” signaling pathway, which may be linked to cognitive impairment (Table 5, Supplementary Materials Table S6) [147].

Proteomic analysis of neuronal and glial cells showed that MPs adsorb proteins from biological fluids, forming a heterogeneous corona layer that alters the expression of proteins related to protein synthesis, RNA processing, lipid metabolism, and nucleocytoplasmic transport. This disrupts cellular signaling pathways, promoting MPs accumulation in the brain and enhancing neurotoxicity through cell recognition mechanisms [148]. In mice, polystyrene MPs caused cognitive impairment and anxious behavior, with accumulation in the brain and pathological changes in the limbic system. A reduction in the total number of cells and dystrophic changes affecting anxiety and fear responses were observed [139]. In African catfish, exposure to polyethylene MPs altered the activity of neurological enzymes and caused histological changes in the brain, including neuron deformation, encephalomalacia, gliosis, spongiosis, Purkinje cell pyknosis, and necrosis. The addition of chlorella, citric acid, or lycopene restored enzyme activity and histological structure, especially with chlorella, highlighting their neuroprotective potential (Figure 4, Table 5, Supplementary Materials Table S6) [149].

**Table 5:** Experimental studies on neurotoxicity and neurodevelopmental toxicity of MNPs: models, particle types, concentrations, and key effects (n = 34 primary sources). Arrows ↑ and ↓ denote increase and decrease, respectively. Abbreviations: MNPs—micro- and nanoplastics; MP—microplastics; NP—nanoplastics; PS-MP—polystyrene microplastic; PS-NP—polystyrene nanoplastic; PE-MP—polyethylene microplastic; PVC-MP—polyvinyl chloride microplastic; PLA—polylactic acid; PP—polypropylene; virgin—pristine/untreated plastic particles (no additives or aging); hpf—hours post-fertilization; hNSC—human neural stem cells; BBB—blood–brain barrier; ROS—reactive oxygen species; mtROS—mitochondrial ROS; LPO—lipid peroxidation; MDA—malondialdehyde; GSH—glutathione; GPX4—glutathione peroxidase 4; SOD—superoxide dismutase; CAT—catalase; AChE—acetylcholinesterase; DA—dopamine; 5-HT—serotonin (5-hydroxytryptamine); ACh—acetylcholine; BDNF—brain-derived neurotrophic factor; NO—nitric oxide; KYN—kynurenine; 3-HK—3-hydroxykynurenine; TLR4—Toll-like receptor 4; NF-κB—nuclear factor kappa B; NLRP3—NOD-like receptor family pyrin domain containing 3; SIRT3—sirtuin 3; DOCK3—dedicator of cytokinesis 3; ErbB4—erb-b2 receptor tyrosine kinase 4; E4A—ErbB4 agonist; MeHg—methylmercury; Cu—copper; Cd—cadmium; DEHP—di(2-ethylhexyl) phthalate; AgNP—silver nanoparticles; MT—methyltestosterone; AMI—amitriptyline; LYC—lycopene; MCU—mitochondrial calcium uniporter; MICU3—mitochondrial calcium uptake family member 3; DBcAMP—dibutyryl cyclic AMP; MCU-i4—MCU inhibitor 4; HDAC6—histone deacetylase 6; ACY-1215—HDAC6 inhibitor; Wnt—Wingless/Integrated; N-cadherin—neural cadherin; PI3K/AKT—phosphoinositide 3-kinase/protein kinase B; GSK-3β—glycogen synthase kinase 3 beta; mTOR—mechanistic target of rapamycin; GABA—gamma-aminobutyric acid; Glu—glutamate; •OH—hydroxyl radical; NH_3_—ammonia; 8-OHdG—8-hydroxy-2′-deoxyguanosine; FOXO3a—forkhead box O3a; PERK—protein kinase R-like endoplasmic reticulum kinase; CHOP—C/EBP homologous protein; GRP78—glucose-regulated protein 78; ER—endoplasmic reticulum; ECM—extracellular matrix; DEGs—differentially expressed genes; PEPP—protein extractable with perchloric acid; PP-MF—polypropylene microfibers; bEnd.3—brain endothelial cell line (mouse); hCMEC/D3—human cerebral microvascular endothelial cell line (D3).

№	Experimental Model	Type and Source of MP/NP	Concentrations	Results/Observations	Reference
1.	Mice (in vivo, oral administration) + cerebral organoids (in vitro)	PS-NP, virgin	2.5 mg/mL, 200 µL (mice, 7 days); 10 mg/mL (organoids, 21 days, 50, 100 nm)	Deep penetration of 50 nm NP, ↓ organoid viability, DNA fragmentation (hippocampus/cortex), ↑ KYN/3-HK, kynurenine pathway activation, neuroinflammation	[20]
2.	Mice (in vivo, oral administration) + E4A	PS-MP, virgin	2 µm, 50 mg/kg, oral, every 2 days, 4 weeks; E4A (400 ng/kg, i.p., daily, weeks 2–4)	Neuronal damage, ↓ DOCK3/SIRT3, ↑ TLR4-NF-κB-NLRP3, E4A (ErbB4 agonist) restores cognitive functions	[21]
3.	Chickens (in vivo, yolk injection)	PS-NP, virgin	60–900 nm	Neural tube defects, ↑ autophagy, apoptosis	[29]
4.	Human neural stem cells (hNSC, in vitro)	PS-NP, virgin	1–50 µg/mL (50–200 nm)	↓ proliferation, ↑ apoptosis (endocytosis, no nuclear entry); smaller particles more toxic	[30]
5.	Carp (head kidney lymphocytes, in vitro)	PS-MP, virgin	5–150 µM	Necroptosis, ↑ mtROS, Ca^2+^, cytokines; astaxanthin ↓ toxicity via miR-25-5p/MCU	[32]
6.	Zebrafish (larvae, in vivo)	PS-MP, virgin	PS-MP: 0.5–25 mg/L, 4–144 hpf; melatonin (1 µM)	↓ swimming speed, ↓ motor neuron axon length, apoptosis, suppression of neurodevelopmental genes, melatonin ↑ nrf2-isl2a	[119]
7.	Zebrafish (larvae, in vivo) + PS-MP/NP + AgNP	PS-MP/NP (polystyrene micro/nanospheres) + AgNP	200 µg/L MP (5 µm/100 nm) + 10 µg/L Ag (5 nm)	Developmental abnormalities, ↓ locomotion, ↑ ROS, neuroinflammation, gene dysregulation (immunity/apoptosis/neurogenesis); curcumin mitigates (especially NP)	[120]
8.	Zebrafish (embryos, in vivo)	PS-MP, virgin	0.1, 1, 10 ppm (500 nm), 96 h	No effect on survival/hatching; ↓ swimming, ↑ apoptosis, ↓ AChE, ↑ NO, dysregulation of 5-HT/DA, ↓ bdnf; styrene binds Bcl-2/p53/BDNF	[121]
9.	*C. elegans* (in vivo)	PS-NP, virgin	10–100 µg/mL (25, 50, and 100 nm)	Dose-dependent: ↑ ROS, mitochondrial damage, ↓ DA, growth/locomotion inhibition; smaller NP less toxic; regulation of sel-12/hop-1	[122]
10.	Cerebral organoids (in vitro)	PS-NP, virgin	0.1–100 µg/mL	↓ viability/differentiation, mitochondrial dysfunction, ↓ Wnt pathway, ↓ N-cadherin, axon/synapse disruption	[123]
11.	Rats (postnatal, via mother)	PS-NP, virgin	2.5 mg/kg from day 1 of pregnancy to postnatal day 21 (50 nm)	Ferroptosis in offspring hippocampus (NCOA4/p53), ↑ ROS, ↓ GPX4/GSH, ↓ cognitive functions/memory	[124]
12.	Zebrafish (in vivo) + amitriptyline	PS-MP + amitriptyline	50 µm, 100–10^6^ particles/L + 48 ± 33 µg/L amitriptyline	↑ AChE/carboxylesterases, swimming changes; MP does not modulate amitriptyline	[125]
13.	Brown trout juveniles (*Salmo trutta* f. *fario*)	Irregular PS	<50 µm; 10^4^ and 10^5^ particles/L; 3 weeks	Amitriptyline (1–1000 µg/L): ↑ AChE activity, swimming behavior changes; >300 µg/L—acute side effects; PS-MP alone: no effects; combination: similar to amitriptyline, but high PS conc. ↓ behavioral impact	[126]
14.	Zebrafish (in vivo) + amitriptyline	PS-MP + amitriptyline	MP (2 µm, 0.44 mg/L), AMI (2.5 µg/L) and mixtures for 7 days	↑ locomotion/schooling, ↓ SOD/CAT/GSH in eyes; additive behavioral toxicity	[127]
15.	Mice (in vivo, oral administration) + melatonin	PS-MP, virgin	0.5–5 mg/kg/day (8 weeks)	Seizures,↑ inflammation/ferroptosis in hippocampus, Fe/lipid disruption, melatonin ↓ seizures	[128]
16.	Rats (in vivo, oral administration)	PE-MP, virgin	1–100 mg/kg/day (<30 µm, 3–6 weeks)	BBB disruption, ↑ MDA, ↓ antioxidants, ↓ BDNF	[129]
17.	Mice (in vivo, oral administration) + in vitro (hCMEC/D3)	PS-NP, virgin	0.5–50 mg/kg (7 days)	BBB penetration, microglial activation, endothelial necroptosis	[130]
18.	Zebrafish (in vivo) + MeHg	PS-MP + MeHg	5 µm, 24 h	Enhanced MeHg neurotoxicity: ↓ locomotion, glutathione disruption	[131]
19.	Rats (in vivo, oral administration)	PS-MP, virgin	0.01 mg/kg/day (42 nm, 56 days)	↑ LPO, NO, •OH, NH_3_, Glu; ↓ GABA, antioxidants, mTOR; LYC restores balance, ↓ stress, ↑ lysosomes	[132]
20.	Olfactory bulbs, n = 15 (autopsies, São Paulo)	16 synthetic polymers: 75% particles, 25% fibers; PP 43.8%	8/15 positive; particles 5.5–26.4 µm, fibers ~21 µm	Absent in controls; olfactory translocation pathway to brain; risk of neurotoxicity, BBB	[133]
21.	BBB model (in vitro, bEnd.3 + TNF-α)	PS-MP, virgin	0–200 µg/mL; 24/72 h	↑ BBB permeability 15.6–27.3-fold (>1 µm), TNF-α enhancement; size-dependent	[134]
22.	Zebrafish (in vivo) + Cu	PVC-MP + Cu	0.5 mg/L MP + 0.85 mg/L Cu (60 days)	Apoptosis via BDNF/miR132/FOXO3a, ↑ ROS/LPO/carbonyls/8-OHdG, ↓ DA/5-HT/ACh, neuroarchitectural damage	[135]
23.	Mice (in vivo, oral administration) + DEHP	PS-NP + DEHP	775 mg/L MPs–25 µM DEHP	Neuronal apoptosis (PI3K/AKT), mitochondrial dysfunction, ↑ GSK-3β, brain pathology	[136]
24.	PLA-NP (in vivo → in vitro degradation)	PLA, biodegradable	2.5–25 mg/kg/day, oral, 28 days	Degradation to oligomers, ↑ Ca^2+^ in mitochondria (↑ MICU3), MCU-i4/DBcAMP ↓ toxicity	[138]
25.	Mice (in vivo, oral administration)	PS-MP, virgin	MP 0.1–1 mg/mL, 15–60 days (acute/subchronic)	Cognitive impairments, anxiety, brain accumulation, limbic system dystrophy	[139]
26.	*Pomacanthus microps* (in vivo) + Cd	PE-MP + Cd	0.14 mg/L PE-MP (1–5 µm) + 0–13 mg/L Cd (96 h)	↓ AChE, ↓ PEPP; MP–Cd antagonism	[140]
27.	Mice (in vivo, oral administration) + miR-103a-3p	PS-MP, virgin	30 mg/kg/day (5 µm, 7 days)	Cognitive impairments, ↑ MDA/carbonyl/nitrite/caspases/TNFα/NLRP3, ↓ SIRT1/BDNF, ER stress (PERK/CHOP/GRP78), miR-103a-3p restores	[141]
28.	Astrocytes (in vitro)	PS-MP/NP, virgin	0.01–1000 µg/mL (50, 500 nm)	↓ astrocyte viability, astrogliosis; 1274/531 DEGs (inflammation/immunity/migration/ECM/lipids)	[142]
29.	Mice (in vivo, intranasal)	PS-NP, virgin	100 nm	Brain accumulation, neurotoxicity, ACY-1215 (HDAC6 inhibitor) enhances NP exocytosis	[144]
30.	Cerebral organoids (in vitro)	PS-NP, virgin	~50 nm, 200 ng/mL, organoids 24 days	Mitochondrial dysfunction, ↓ viability/differentiation	[145]
31.	Zebrafish (in vivo) + ketamine/methionine	PS-MP + ketamine/methionine	PP-MF: <2 mm, 2 mg/L (with feed), 7 days	Enhanced tectum lesions: ↑ ROS, necrosis, edema	[146]
32.	Zebrafish (in vivo) + methyltestosterone	PS-MP + methyltestosterone	50 ng/L MT and 0.5 mg/L PS (5 µm diameter) 21 days	Neuronal damage, dysregulation of neuroactive ligand-receptor interactions	[147]
33.	Neurons/glia (in vitro, proteomics)	Various MP (PE/PP/PS)	1–100 µg/mL, 24–72 h	Heterogeneous protein corona formation, altered protein expression (synthesis/RNA/lipids/transport)	[148]
34.	African catfish (in vivo, oral administration)	PE-MP, virgin	MP 500 mg/kg diet, 15 days; chlorella (50 g/kg), citric acid (30 g/kg), lycopene (500 mg/kg)	Altered neurological enzymes, neuronal deformation, encephalomalacia, gliosis, Purkinje pyknosis, necrosis; chlorella/citric acid/lycopene restore	[149]

### 3.5. Enteric Nervous System and Gut–Brain Axis

MPs and NPs accumulate in the gastrointestinal tract (GIT), causing changes in the enteric nervous system (ENS), microbiota dysbiosis, and structural disruptions that can enhance neurotoxicity via the gut–brain axis. These effects are linked to impaired neuronal populations, inflammation, and altered neurotransmitters, affecting the central nervous system (Table 6, Supplementary Materials Table S7).

In sows, exposure to PET-MPs in the ileum caused a reduction in the population of neurons positive for CART, GAL, nNOS, VAChT, and VIP in the submucosal and myenteric plexuses, with an increase in GAL- and SP-positive neurons. Histological analysis revealed thinning of the mucosal and muscle layers, although levels of cytokines such as IL-1β, IL-6, IL-8, IL-10, and TNF-α did not change significantly. These changes indicate neuronal damage and ENS dysfunction [150]. *Danio rerio*, polylactic acid (PLA) and aged PLA (APLA) caused thinning of the intestinal wall, shortening of villi, microbiota dysbiosis, and neurotransmitter suppression, which was accompanied by neurotoxicity via the gut–brain axis. Bile acid supplementation reduced these effects, highlighting the role of the microbiota in neurotoxicity [54]. In mice, 30- and 60-day exposure to polystyrene MPs/NPs (PS-MPs/NPs) caused anxiety-like behavior in open-field and elevated plus-maze tests. 16S rRNA sequencing showed a reduction in beneficial microbiota and an increase in pathogenic bacteria, with decreased intestinal mucus secretion and increased intestinal permeability. Metabolomic analysis revealed changes in ABC transporter pathways, aminoacyl-tRNA biosynthesis, amino acids, and bile secretion, correlating with anxious behavior and neurotransmitter dysregulation [151]. In adolescent mice, PS-NPs caused cognitive impairments linked to changes in the gut microbiota and hippocampal metabolome, with dysregulation of the PI3K/AKT pathway [152]. In mice, PE-MPs accumulated through the food chain, causing anxious behavior and reduced locomotion (Table 6, Supplementary Materials Table S7) [153].

These evidence confirms that MPs and NPs cause ENS damage, dysbiosis, and structural changes, affecting neurotoxicity via the gut–brain axis. Protective agents, such as bile acid, show potential for mitigating the effects, but require further research.

### 3.6. Psychiatric Disorders

#### 3.6.1. Depression

MPs and NPs are increasingly being considered potential factors contributing to the development of psychiatric disorders, including depression, due to their ability to cause neuroinflammation, oxidative stress, and neurotransmitter balance disruption. Epidemiological and experimental studies on animal models demonstrate that MPs and NPs exposure is associated with depressive-like behavior (Table 7, Supplementary Materials Table S8).

**Figure 5:**
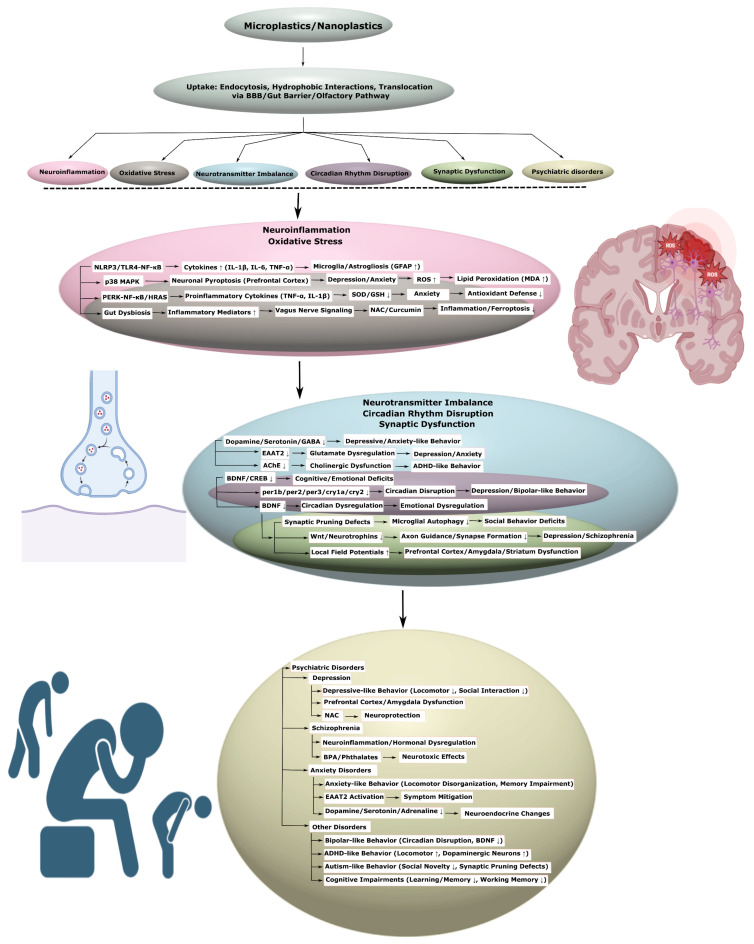
Conceptual diagram of the molecular-cellular mechanisms of microplastics and nanoplastics in the pathogenesis of psychiatric disorders. The diagram illustrates particle uptake via endocytosis, hydrophobic interactions, and translocation through the blood–brain barrier (BBB), intestinal barrier, or olfactory pathway, triggering pathways of neuroinflammation (NLRP3, TLR4-NF-κB, p38 MAPK, PERK-NF-κB, HRAS, IL-1β, IL-6, TNF-α), oxidative stress (ROS, MDA, SOD, GSH), neurotransmitter imbalance (Dopamine, Serotonin, GABA, EAAT2, AChE, BDNF, CREB), circadian rhythm disruption (per1b, per2, per3, cry1a, cry2), and synaptic dysfunction (Wnt, Neurotrophins). As a result, depression, schizophrenia, anxiety disorders, bipolar disorder, ADHD, autism, and cognitive impairments may develop. Based on in vitro and in vivo studies. Arrows (→) indicate process continuation, ↑ denotes increase, and ↓ denotes decrease. Abbreviation Key: BBB—blood–brain barrier, NLRP3—NOD-like receptor protein 3, TLR4—Toll-like receptor 4, NF-κB—Nuclear factor kappa B, IL-1β—Interleukin-1β, IL-6—Interleukin-6, TNF-α—Tumor necrosis factor-α, p38 MAPK—p38 Mitogen-activated protein kinase, PERK—Protein kinase R-like endoplasmic reticulum kinase, HRAS—Harvey rat sarcoma homolog, ROS—Reactive oxygen species, MDA—Malondialdehyde, SOD—Superoxide dismutase, GSH—Glutathione, NAC—N-acetylcysteine, GABA—Gamma-aminobutyric acid, EAAT2—Excitatory amino acid transporter 2, AChE—Acetylcholinesterase, BDNF—Brain-derived neurotrophic factor, CREB—cAMP response element-binding protein, per1b, per2, per3—Period genes 1b, 2, 3, cry1a, cry2—Cryptochrome genes 1a, 2, Wnt—Wingless-related integration site, GFAP—Glial fibrillary acidic protein, BPA—Bisphenol A, ADHD—Attention deficit hyperactivity disorder.

High MP exposure was linked to an increased likelihood of depressive symptoms among university students. Participants with the highest exposure level had a 38% greater probability of depression compared to the lowest quartile, with each 1000-particle increase in exposure raising the risk by 7%. This link was more pronounced in men and first-year students [45]. In mice, chronic exposure to polystyrene NPs caused depressive-like behavior linked to the activation of axonal guidance, neurotrophin, and dopaminergic synapse signaling pathways, confirmed by mRNA, microRNA, and dnRNA analysis [154]. In *Danio rerio*, polystyrene MP exposure reduced exploratory and locomotor activity, as well as social interaction, accompanied by increased IL-6, IL-1β, and circadian rhythm dysregulation through altered expression of per1b, per2, per3, cry1a, and cry2 genes [46]. In mice, PS-NP exposure induced depressive-like behavior by suppressing the expression of the glutamate transporter protein EAAT2 in astrocytes, which was partially restored upon EAAT2 activation [47]. Co-exposure to PS-NPs and ozone enhanced neuroinflammation and neuronal pyroptosis in the prefrontal cortex through activation of the p38 MAPK pathway, causing depressive symptoms that were mitigated by the antioxidant N-acetylcysteine (NAC) [155]. PS-NP exposure during pregnancy in mice caused anxious and depressive-like behavior in the offspring, linked to suppressed GABA synthesis in the prefrontal cortex and amygdala, which was alleviated by NAC (Figure 5, Table 7, Supplementary Materials Table S8) [48].

These data indicate neuroinflammation, oxidative stress, and neurotransmitter dysregulation as key mechanisms linking MPs and NPs to depression.

#### 3.6.2. Schizophrenia

Although the link between MPs and NPs and schizophrenia is insufficiently studied, available data indicate their potential role through neuroinflammation and hormonal dysregulation, requiring further research. The detection of MPs in human brain tissues underscores the importance of studying their long-term impact on psychiatric disorders.

Plastic chemicals associated with MPs, such as bisphenols and phthalates, can cause neuroinflammation, which is a key factor in the pathogenesis of schizophrenia (Figure 5) [156]. In Danio rerio using schizophrenia models induced by ketamine and methionine, polypropylene MPs consumed in food acted as a buffer against ketamine toxicity, but enhanced the effects of methionine. Groups with higher anxiety levels showed improved memory, which indicates a complex interaction of MPs with neurotoxicants (Table 7, Supplementary Materials Table S8) [157].

#### 3.6.3. Anxiety Disorders

MPs and NPs may contribute to the development of anxiety disorders by influencing neuroinflammatory and neurotransmitter systems. Studies on animal models demonstrate that MP and NP exposure induces anxious behavior linked to oxidative stress and impaired synaptic transmission, highlighting their neurotoxic potential.

In mice, PS-NP exposure induced anxious behavior linked to impaired synaptic transmission and increased expression of the astrocyte marker GFAP in the medial prefrontal cortex. EAAT2 activation mitigated these effects [47]. Co-exposure to PS-MPs and low temperatures in mice caused locomotor disorganization, anxious behavior, and memory impairments, linked to neuroendocrine changes, including dysregulation of dopamine, serotonin, adrenaline, corticosterone, and oxidative stress in brown adipose tissue [49]. In *Danio rerio*, early-life PS-MP exposure elicited attention-deficit/hyperactivity disorder (ADHD)-like behaviors by disrupting dopaminergic neurodevelopment, with a 30% increase in the number of dopaminergic neurons [158]. In *C. elegans*, polystyrene MPs/NPs increased movement frequency and damaged cholinergic and GABAergic neurons, indicating excitatory toxicity, mitigated by the antioxidant curcumin [159]. In *Danio rerio*, PS-NPs caused changes in locomotor activity, aggressiveness, and circadian rhythms, accompanied by oxidative stress and neurotransmitter dysregulation, such as serotonin and dopamine [160]. Also, in mice, PS-NP exposure during pregnancy led to anxious behavior in the offspring through mechanisms of oxidative stress and reduced GABA [48]. PS-MPs induced anxiety through activation of the PERK-NF-κB signaling pathway, mediated by HRAS, in microglia, with increased pro-inflammatory cytokines TNFα and IL-1β [161]. *C. elegans*, PS-COOH caused neurotoxicity, affecting neurotransmission of dopamine, glutamate, serotonin, and GABA (Figure 5, Table 7, Supplementary Materials Table S8) [162].

#### 3.6.4. Other Psychiatric Disorders

MPs and NPs may be associated with other psychiatric disorders. For example, these particles may be linked to bipolar disorder, suicidal behavior, and epilepsy, etc.

An epidemiological study among children aged 6–9 in Shenyang, China, found that increased levels of MPs in urine correlate with emotional problems, behavioral difficulties, hyperactivity, and peer problems. These changes may be relevant to affective disorders, including bipolar disorder, although direct data are insufficient [50]. In *Danio rerio*, exposure to biodegradable MPs reduced survival, increased wakefulness, and caused anxious behavior, disrupting circadian rhythms through effects on BDNF. These changes suggest a potential link to disorders involving circadian rhythm disruption, which are sometimes associated with bipolar disorder [163]. In mice, NP exposure at different developmental stages altered the dopaminergic system and reduced social behavior, especially in late pregnancy and adulthood, with increased local potentials in the prefrontal cortex, amygdala, and striatum [164]. In neonatal mice, PS-NPs disrupted microglial autophagy and energy metabolism, leading to defects in synaptic pruning and social behavior in adulthood [165]. In children aged 7 to 10 in Shenyang, high levels of MPs in urine correlated with impaired working memory and increased inattention, with a dose-dependent effect (Figure 5, Table 7, Supplementary Materials Table S8) [166].

MPs and NPs are also linked to cognitive impairments and behavioral changes that may be relevant to other psychiatric disorders. In mice, chronic exposure to polystyrene MPs impaired learning and memory in the Morris water maze test, causing oxidative stress and reduced acetylcholine level and CREB/BDNF pathway activity (Figure 5). Vitamin E mitigated these effects, indicating oxidative stress as a key mechanism [167]. In mice, chronic exposure to polystyrene MPs from the embryonic stage caused impairments in social novelty, which may suggest autism spectrum disorders Table 7, Supplementary Materials Table S8) [168].

**Table 7:** Experimental studies on the role of MNPs in mental disorders: models, particle types, concentrations, and key effects (n = 20 primary sources). Arrows ↑ and ↓ denote increase and decrease, respectively. Abbreviations: MNPs—micro- and nanoplastics; MP—microplastics; NP—nanoplastics; PS-MP—polystyrene microplastic; PS-NP—polystyrene nanoplastic; PP-MP—polypropylene microplastic; PLA-MP—polylactic acid microplastic; PS-COOH—carboxylated polystyrene; virgin—pristine/untreated plastic particles (no additives or aging); IL-6—interleukin 6; IL-1β—interleukin 1 beta; EAAT2—excitatory amino acid transporter 2; GABA—gamma-aminobutyric acid; NAC—N-acetylcysteine; DA—dopamine; 5-HT—serotonin (5-hydroxytryptamine); ROS—reactive oxygen species; ADHD—attention-deficit/hyperactivity disorder; per1b/per2/per3—period circadian regulators; cry1a/cry2—cryptochrome circadian regulators; p38 MAPK—p38 mitogen-activated protein kinase; BDNF—brain-derived neurotrophic factor; PERK—protein kinase R-like endoplasmic reticulum kinase; NF-κB—nuclear factor kappa B; HRAS—Harvey rat sarcoma viral oncogene homolog; TNFα—tumor necrosis factor alpha; Glu—glutamate; ACh—acetylcholine; CREB—cAMP response element-binding protein; GD—gestational day; PND—postnatal day.

№	Experimental Model	Type and Source of MP/NP	Concentrations	Results/Observations	Reference
1.	1420 college students	Various MP (environmental exposure)	Exposure quartiles (particles/day)	↑ 38% depression risk in top quartile; +7% per 1000 particles; stronger in males/freshmen	[45]
2.	Zebrafish (in vivo, water)	PS-MP, virgin	25 and 250 µg/L, 40 days	↓ exploratory/locomotor activity, social interaction; ↑ IL-6/IL-1β; dysregulation of per1b/per2/per3/cry1a/cry2	[46]
3.	Mice (in vivo, oral)	PS-NP, virgin	10 mg/kg	Depression-like behavior; ↓ EAAT2 in astrocytes; EAAT2 activation mitigates	[47]
4.	Mice (prenatal, in vivo)	PS-NP, virgin	100 nm/1 µm, 1 mg/day, oral, 17 days	Anxiety/depression in offspring; ↓ GABA in prefrontal cortex/amygdala; NAC restores	[48]
5.	Mice (in vivo, oral) + cold stress	PS-MP, virgin	10 mg/kg/day, 21 days	Locomotor disorganization, anxiety, ↓ memory; DA/5-HT/adrenaline/corticosterone dysregulation; ↑ ROS in brown adipose tissue	[49]
6.	Children 6–9 years (n = 1000, Shenyang)	Various MP (in urine)	Concentration quartiles (particles/mL)	↑ emotional/behavioral problems, hyperactivity, peer issues	[50]
7.	Mice (in vivo, oral)	PS-NP, virgin	0–50 mg/kg/day (8 weeks)	Depression-like behavior; activation of axonal guidance/neurotrophins/dopaminergic synapses (mRNA/miRNA/lncRNA)	[154]
8.	Mice (in vivo, inhalation) + ozone	PS-NP, virgin	60 nm, 12.5 mg/kg/day, oral, 30 days	↑ neuroinflammation/pyroptosis in prefrontal cortex (p38 MAPK); depression; NAC mitigates	[155]
9.	Zebrafish (in vivo, ketamine/methionine model)	PP-MP, virgin	0.1–100 µg/mL	Buffers ketamine, ↑ methionine effects; memory improvement under high anxiety	[157]
10.	Zebrafish (early life, in vivo)	PS-MP, virgin	0.1 µm and 5 µm, 0, 0.1, 10, 100 µg/mL	ADHD-like behavior; ↑ locomotion, +30% dopaminergic neurons	[158]
11.	*C. elegans* (in vivo)	PS-MP/NP, virgin	100 nm/0.5/1.0/2.0/5.0 µm, 1.0 mg/L, 3 days	↑ movement, damage to cholinergic/GABAergic neurons; curcumin mitigates	[159]
12.	Zebrafish (in vivo)	PS-NP, virgin	0.5, 1.5, 5 ppm; acute: 7 days; chronic: 30 days/7 weeks	Locomotion/aggressiveness/circadian rhythm changes; ↑ ROS; 5-HT/DA dysregulation	[160]
13.	Mice (in vivo, oral), in vitro (BV2 microglia)	PS-MP, virgin	2 mg/kg and 10 mg/kg (mice, 7 days), 25, 50, 100 µg/mL (cells, 24, 48, 72 h)	Anxiety; ↑ PERK-NF-κB (HRAS) in microglia, ↑ TNFα/IL-1β	[161]
14.	*C. elegans* (in vivo)	PS-COOH (carboxylated), virgin	0.1–100 µg/L	Neurotoxicity; DA/Glu/5-HT/GABA dysregulation	[162]
15.	Zebrafish (in vivo, biodegradable)	PLA-MP (polylactic acid), virgin	1, 25, 50, 100, 250, 500 mg/L (up to 5 days)	↓ survival, ↓ wakefulness, anxiety; circadian rhythm/BDNF dysregulation	[163]
16.	Mice (in vivo, different life stages)	PS-NP, virgin	0.1–1 mg/kg/day (GD1–PND21 or adults)	Dopamine system changes, ↓ social behavior; ↑ local field potentials (prefrontal cortex/amygdala/striatum)	[164]
17.	Neonatal mice (in vivo)	PS-NP, virgin	100 nm, 7 days	Microglial autophagy impairment, energy metabolism defects; synaptic pruning defects, ↓ social behavior	[165]
18.	Children 7–10 years (n = 5670, Shenyang)	Various MP (in urine)	Dose-dependent (particles/mL)	↓ working memory, ↑ inattention	[166]
19.	Mice (in vivo, chronic)	PS-MP, virgin	0.01, 0.1, 1 mg/day (4 weeks)	↓ learning/memory (Morris water maze); ↑ ROS,↓ ACh, CREB/BDNF; vitamin E mitigates	[167]
20.	Mice (embryonic, in vivo), in vitro (primary cortical neurons)	PS-MP, virgin	2 µm, 1 mg/kg/day (dam), 0, 1, 10 mg/L (neurons)	Impaired social novelty recognition (autism-like)	[168]

### 3.7. Reproductive System

**Figure 6:**
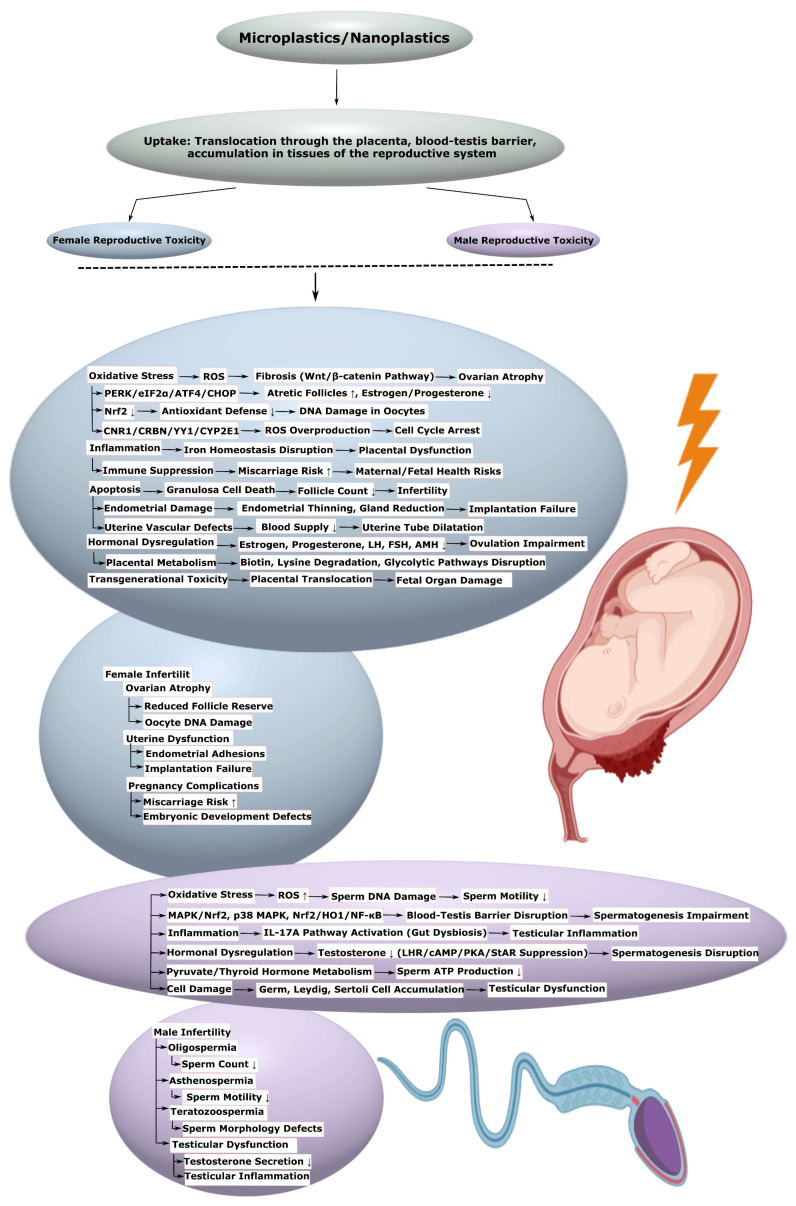
Conceptual diagram of the molecular-cellular mechanisms of microplastics and nanoplastics in the pathogenesis of reproductive system disorders. The diagram illustrates particle uptake and translocation through the placenta and blood-ovarian barrier, with accumulation in ovarian and testicular tissues, triggering pathways of female reproductive toxicity (oxidative stress [ROS, Nrf2], inflammation [IL-6], apoptosis, hormonal dysregulation [Estrogen, Progesterone, LH, FSH, AMH], transgenerational toxicity) and male reproductive toxicity (oxidative stress [ROS, MAPK, Nrf2], inflammation [IL-17A], hormonal dysregulation [Testosterone, LHR, cAMP, PKA, StAR], cell damage). Arrows (→) indicate process continuation, ↑ denotes increase, and ↓ denotes decrease. As a result, female infertility (ovarian atrophy, uterine dysfunction, pregnancy complications) and male infertility (oligospermia, asthenospermia, teratozoospermia, testicular dysfunction) may develop. Based on in vitro and in vivo studies. Abbreviation Key: ROS—Reactive oxygen species, Wnt—Wingless-related integration site, β-catenin—β-Catenin, PERK—Protein kinase R-like endoplasmic reticulum kinase, eIF2α—Eukaryotic translation initiation factor 2α, ATF4—Activating transcription factor 4, CHOP—C/EBP homologous protein, Nrf2—Nuclear factor erythroid 2-related factor 2, CNR1—Cannabinoid receptor 1, CRBN—Cereblon, YY1—Yin Yang 1 (transcription factor), CYP2E1—Cytochrome P450 2E1, IL-6—Interleukin-6, LH—Luteinizing hormone, FSH—Follicle-stimulating hormone, AMH—Anti-Müllerian hormone, MAPK—Mitogen-activated protein kinase, p38 MAPK—p38 Mitogen-activated protein kinase, HO1—Heme oxygenase-1, NF-κB—Nuclear factor kappa B, IL-17A—Interleukin-17A, LHR—Luteinizing hormone receptor, cAMP—Cyclic adenosine monophosphate, PKA—Protein kinase A, StAR—Steroidogenic acute regulatory protein.

Reproductive health, as one of the most important indicators of quality of life, is closely linked to the state of the human reproductive system and can change under the influence of many factors [169,170]. In recent years, the negative impact of MNPs on fertility has been widely investigated in animal models. Data show that MNPs cause reproductive toxicity by disrupting the structure and function of the uterus, ovaries, and endocrine glands, as well as the hypothalamic-pituitary axis [51,52]. Exposure to these particles causes fibrosis in these organs due to the accumulation of reactive oxygen species with the activation of relevant signaling pathways [171,172]. The activation of oxidative stress, inflammation, and apoptosis can affect the processes of spermatogenesis and ovulation and generally compromise fertility and health [173]. By causing apoptosis in granulosa cells and reducing the number of follicles in the ovaries, these substances alter androgen production levels and then disrupt the reproductive endocrine system (Figure 6, Table 8, Supplementary Materials Table S9) [174].

#### 3.7.1. Female Reproductive Health

In human studies in recent years, special attention has been paid to the negative impact of MNPs on female fertility. Available data indicate a significant link between constant MNP exposure and reduced female fertility [175,176]. These substances, accumulating in human reproductive organs and exerting a toxic effect, can disrupt their function [177]. These particles have been shown to damage cellular components through intracellular pathways and disrupt the cell cycle [178]. MNPs, in addition to maternal damage, passing through the placenta during pregnancy and lactation and penetrating various fetal organs, including the heart, liver, lungs, and spleen [179], and affecting their reproductive and nervous systems, cause transgenerational toxicity and disrupt embryonic development [180,181]. The penetration of these particles into the human trophoblast and the altered gene expression in its cells can lead to general disruptions in the maternal and fetal immune systems [182]. After the maternal immune system is suppressed due to exposure to these substances, the risk of miscarriage increases, and maternal health is also endangered (Figure 6, Table 8, Supplementary Materials Table S9) [183]. The toxic effect of MNPs and the vulnerability of the female reproductive system to these exogenous substances have raised concerns about female fertility and focused great attention on identifying these environmental hazards [184].

Fundamental research has shown that MNP exposure can lead to ovarian inflammation, oxidative stress, endoplasmic reticulum (ER) stress, and apoptosis, resulting in ovarian atrophy [185,186]. Furthermore, MNPs negatively affect hormone secretion, indicated by reduced levels of estrogen, progesterone, LH, FSH, and AMH in female rats [187,188]. Specifically, MNPs induce oxidative stress and activate the PERK/eIF2α/ATF4/CHOP signaling pathway in juvenile mice, correlating with an increased proportion of atretic follicles in the ovaries and a significant reduction in serum estrogen and progesterone levels [189]. MNPs lead to a noticeable reduction in the antioxidant protective role of the Nrf2 protein in mouse and COV434 ovarian cells [190]. Additionally, MNPs are suggested to cause fibrosis by activating the Wnt/β-catenin signaling pathway, leading to granulosa cell apoptosis through oxidative stress in rats, and ultimately reducing ovarian reserve function [171]. MNP exposure can cause DNA damage in mouse oocytes. MNPs alter spindle assembly and Juno localization in mouse oocytes after ingestion, directly affecting sperm-egg fusion [191]. In vitro experiments with cultured ovarian granulosa cells demonstrated a synergistic effect between MNPs and DEHP, promoting ROS overproduction and causing oxidative stress through the CNR1/CRBN/YY1/CYP2E1 signaling axis. This led to oxidative DNA damage and cell cycle arrest (Figure 6, Table 8, Supplementary Materials Table S9) [192].

The uterus is important for reproductive function. MNPs exposure correlates with reduced uterine mass, thinning of endometrial layers, decreased endometrial gland count, and reduced viability of endometrial epithelial cells in mice [188,191]. MNPs may cause infertility problems such as endometrial adhesions, narrowing of the uterine cavity, and extensive cavities in the uterine wall. MNPs exposure is also linked to reduced uterine blood supply, which can lead to a decrease in the number and diameter of uterine vessels due to immune dysregulation [193]. The effect of MNPs on fallopian tube dilation is possibly related to their immunotoxicity [194]. The effect of MNPs on the placenta may be due to disruptions in immune mechanisms, leading to a reduction in its mass [179,180]. The effect of MNPs on placental metabolic function has also been shown [195,196]. Multidimensional analysis determined metabolic profile changes caused by MNPs, revealing disruptions in biotin metabolism, lysine degradation, and glycolytic/gluconeogenic pathways [197]. MNP particles can activate inflammatory factor IL-6 and disrupt iron homeostasis [198]. Notably, maternal lung exposure to MNPs leads to their translocation into the placenta and fetal tissues, making the fetal-placental units vulnerable to adverse effects [179]. Numerous studies consistently demonstrate that maternal MNPs lead to a reduced number of embryos and a decreased implantation rate in mice [174,191]. This adverse effect is linked to oxidative stress [190] and uteroplacental ischemia resulting from indirect vascular defects (Figure 6, Table 8, Supplementary Materials Table S9) [191].

#### 3.7.2. Male Reproductive Health

Although causality has not been definitively established, growing evidence points to chronic toxic MNP exposure as a potential threat to male reproductive health. This emerging hypothesis opens new avenues for research, emphasizing the need to elucidate the mechanisms by which MNPs may negatively influence fertility and reproductive outcomes (Table 8, Supplementary Materials Table S9) [199].

A significant decline in male sperm analysis parameters has been observed over the past decades, and environmental pollutants are a possible cause. Accumulating research has shown that MNP exposure has a detrimental effect on male reproductive function. Studies of plastic particles and human tissues are rare. Nevertheless, animal experiment results have revealed some important evidence. Sperm motility efficiency, including ATP production, sperm viability, and DNA integrity, is reduced upon MNP exposure, thus decreasing the probability of gamete collision [200]. Moreover, upon MNP exposure, activation of the IL-17A signaling pathway, mediated by gut microbiota dysregulation, causes spermatogenesis disruption and suppression of sex hormone synthesis (Figure 6, Table 8, Supplementary Materials Table S9) [201]. Given the regulation between key organs, MNP-induced male reproductive system damage may be secondary to damage in other key organs [61]. Therefore, new research focused on studying MNP-induced disruptions in the brain-testis axis and gut–testis axis systems is of scientific interest.

In recent years, a number of studies have demonstrated that polystyrene microplastics cause reproductive toxicity in male mice. The mechanism of sperm damage by polystyrene microplastics is the disruption of pyruvate and thyroid hormone metabolism [202]. The blood-testis barrier plays a crucial role in maintaining the homeostasis of the seminiferous tubule microenvironment, which is essential for spermatogenesis [203]. Previous studies have shown that its damage can disrupt spermatogenesis, leading to male infertility and conditions such as oligozoospermia, asthenozoospermia, and teratozoospermia, as it is necessary to maintain the biochemical environment required to support spermatogenesis (Table 8, Supplementary Materials Table S9) [204]. Furthermore, most studies on male animals have shown that MPs can disrupt the blood-testis barrier, triggering the MAPK/Nrf2, p38 MAPK, and Nrf2/HO1/NF-B signaling pathways through mechanisms linked to reactive oxygen species. This disruption negatively affects testicular health, reducing semen quality and ultimately leading to male infertility (Figure 6) [205].

Testosterone plays a crucial role in maintaining testicular structural integrity [206]. Long-term exposure to polystyrene microplastic leads to male reproductive toxicity and decreased testosterone levels via LH-mediated suppression of the LHR/cAMP/PKA/StAR pathway [52]. MNP particles can penetrate three types of testicular cells, such as germ cells, Leydig cells, and Sertoli cells, accumulate in the testes, and subsequently cause spermatogenesis disruptions, reduced testosterone secretion, testicular inflammation, and testicular dysfunction (Figure 6, Table 8, Supplementary Materials Table S9) [207].

Recent studies have made significant progress in understanding MNP reproductive toxicity to humans. MNPs appear in human testes and semen, demonstrating for the first time that MNPs enter the male reproductive system [208].

In semen samples from 40 participants undergoing premarital health examination in China, MNP emerge in seminal fluid, even in individuals without occupational exposure. Polystyrene, polyethylene, and polyvinyl chloride rank as the most common MPs and show differential association with progressive sperm motility (Figure 6, Table 8, Supplementary Materials Table S9) [209].

Accordingly, there is an urgent need to understand the effects of MNPs on the male reproductive system in realistic exposure contexts to inform future investigations. Furthermore, more comprehensive and methodologically robust studies are needed to validate these findings and provide a thorough understanding of MPs’ influence on human reproductive health.

**Table 8:** Experimental studies on reproductive toxicity of MNPs: models, particle types, concentrations, and key effects (n = 32 primary sources). Arrows (→) indicate process continuation, ↑ denotes increase, and ↓ denotes decrease. Abbreviations: MNPs—micro- and nanoplastics; MP—microplastics; NP—nanoplastics; PS-MP—polystyrene microplastic; PS-NP—polystyrene nanoplastic; PE—polyethylene; PP—polypropylene; PVC—polyvinyl chloride; PET—polyethylene terephthalate; PC—polycarbonate; virgin—pristine/untreated plastic particles (no additives or aging); PS-COOH—carboxylated polystyrene; PS-NH_2_—aminated polystyrene; G-PS-MPs—graphene-modified polystyrene microplastics; LH—luteinizing hormone; FSH—follicle-stimulating hormone; PLZF—promyelocytic leukemia zinc finger; DAZL—deleted in azoospermia-like; ABP—androgen-binding protein; GnRH—gonadotropin-releasing hormone; LHR—luteinizing hormone receptor; cAMP—cyclic adenosine monophosphate; PKA—protein kinase A; StAR—steroidogenic acute regulatory protein; Wnt—Wingless/Integrated; HMGB1—high mobility group box 1; TLR4—Toll-like receptor 4; NOX2—NADPH oxidase 2; ROS—reactive oxygen species; TGF-β—transforming growth factor beta; Hippo—Hippo signaling pathway; MST1—mammalian sterile 20-like kinase 1; LATS1—large tumor suppressor kinase 1; YAP1—Yes-associated protein 1; CTGF—connective tissue growth factor; Cyr61—cysteine-rich angiogenic inducer 61; GD—gestational day; E2—estradiol; P4—progesterone; PERK—protein kinase R-like endoplasmic reticulum kinase; eIF2α—eukaryotic initiation factor 2 alpha; ATF4—activating transcription factor 4; CHOP—C/EBP homologous protein; Nrf2—nuclear factor erythroid 2-related factor 2; Juno—Juno izumo sperm-egg fusion protein; DEHP—di(2-ethylhexyl) phthalate; CNR1—cannabinoid receptor 1; CRBN—cereblon; YY1—Yin Yang 1; CYP2E1—cytochrome P450 2E1; IL-6—interleukin 6; IL-17A—interleukin 17A; sEH—soluble epoxide hydrolase; UPRT—uracil phosphoribosyltransferase; B3GAT1—beta-1,3-glucuronyltransferase 1; SULT—sulfotransferase; NAT2—N-acetyltransferase 2; CYP1A1—cytochrome P450 1A1; Prm3—protamine 3; Tnp1—transition protein 1; Aurkc—aurora kinase C; Mea1—male enhanced antigen 1; Mettl14—methyltransferase-like 14; Pmfbp1—polyamine modulated factor 1 binding protein 1; Ggn—gametogenetin; Fsip2—fibrous sheath interacting protein 2.

№	Experimental Model	Type and Source of MP/NP	Concentrations	Results/Observations	Reference
1.	Male rats (in vivo, oral)	PS-NP	38.92 nm; 1, 3, 6, 10 mg/kg/day; 5 weeks	↓ testosterone, LH, FSH (serum); dose-dependent testicular damage, ↓ sperm quality (morphology, viability, DNA fragmentation); ↓ PLZF, DAZL, FSH, LH expression (testes); ↑ FSH/LH at 10 mg/kg; nonlinear ABP modulation; ↑ GnRH	[51]
2.	Mice (in vivo, oral)	PS-MP, virgin	100 µg/L and 1000 µg/L (180 days), 0.5 µm, 4 µm, and 10 µm	↓ testosterone (LHR/cAMP/PKA/StAR), reproductive toxicity	[52]
3.	Rats (in vivo, oral)	PS-NP, virgin	0.5 µm, 0, 0.015, 0.15, 1.5 mg/day, 90 days	Ovarian fibrosis (Wnt/β-catenin), granulosa cell apoptosis, ↓ ovarian reserve	[171]
4.	Female mice (in vivo); mouse endometrial epithelial cells (in vitro)	PS-MP, virgin	Oral, various doses	Endometrial thinning, ↑ collagen deposition; ↑ HMGB1, acetyl-HMGB1 → TLR4/NOX2 activation → ↑ ROS → Notch and TGF-β activation → ↑ fibrotic proteins and collagen; TLR4/NOX2 inhibition ↓ ROS, Notch, TGF-β, fibrosis	[172]
5.	Mice, human granulosa-like tumor cells (KGN cells)	PS-NP, virgin	20 nm, 1 mg/day, 5 weeks (in vivo), 100 µg/mL, 48 h (in vitro)	Induction of granulosa cell apoptosis, ROS accumulation, ↑ Hippo (MST1, LATS1, YAP1), ↓ CTGF and Cyr61 mRNA	[173]
6.	Mice (in vivo, oral)	PS-MP/NP, virgin	0.1 mg/day, 30 or 44 days	↓ embryos, ↓ implantation, sperm deformation, oxidative stress	[174]
7.	In vitro (human placental cells)	PS-NP	1–100 µg/mL, 25, 50, 100, 500 nm	Cell damage, cell cycle disruption, oxidative stress, PKA activity inhibition	[178]
8.	Rats (in vivo)	Various MP (PE/PP/PS/PVC)	2.64 × 10^14^ particles, inhalation	Detected in fetal placenta/heart/liver/lungs/spleen	[179]
9.	Human placenta from pregnant women	PS-NP, virgin	Concentration assessment in samples	↓ placental weight, immune disruptions, transgenerational toxicity	[180]
10.	In vitro (human trophoblast), in vivo (mice)	PS-NP, virgin	50 nm, 50 or 100 mg/kg	Gene changes, maternal/fetal immune disruptions, Bcl-2/Cleaved-caspase-2/Cleaved-caspase-3 activation	[182]
11.	Mice (in vivo, oral)	PS-NP, virgin	250 µg in 200 µL saline on GD5.5 and GD7.5	↑ miscarriage risk, maternal immunosuppression	[183]
12.	Rats (in vivo, oral)	PS-MP, virgin	876 nm, 2.5, 5, 10 mg/kg/day, 45 days	Ovarian inflammation, ↑ ROS, ER stress, apoptosis, ovarian atrophy	[185]
13.	Rats (in vivo, oral)	PS-MPs/G-PS-MPs, virgin	0.7918 µm, 30 mg/kg/day (35 days)	Ovarian atrophy, accumulation in heart, liver, spleen, lungs, kidneys, brain, large/small intestine, uterus, ovaries, blood	[186]
14.	Rats (in vivo, oral)	PS-NP, virgin	2.5 mg/kg (6 weeks)	↓ hormones (E2/P4), ↓ uterine weight, endometrial thinning, ↓ glands, ↓ epithelial viability	[188]
15.	Young mice (in vivo, oral)	PS-NP, virgin	1 µm, 0.5 and 2.0 mg/kg/day (28 days)	↑ atretic follicles, ↓ E2/P4 (PERK/eIF2α/ATF4/CHOP)	[189]
16.	Mice (in vivo) + COV434 (in vitro)	PS-NP, virgin	5 and 25 mg/kg/day (mice, 8 weeks), 50, 100, 150, 200 µg/mL (COV434)	↓ Nrf2, ovarian oxidative stress	[190]
17.	Mice (in vivo, oocytes)	PS-NP, virgin	40 mg/kg/day, 30 days	DNA damage, spindle disruption, ↓ Juno, ↓ fertilization	[191]
18.	Granulosa cells (in vitro) + DEHP	PS-NP + DEHP	100 µg/L NP + 200 mg/kg DEHP	↑ ROS, oxidative DNA damage, cell cycle arrest (CNR1/CRBN/YY1/CYP2E1)	[192]
19.	Mice (in vivo) + Caco-2 (in vitro)	PS-NP (~100 nm) virgin/PS-COOH/PS-NH_2_	100 nm, ~1 mg/dose (100 µL × 10 mg/mL)	Apoptosis, inflammation, tissue structure disruption	[193]
20.	Mice (in vivo, oral)	PS-NP, virgin	0.125, 0.5, 2, 90 mg/day/mouse	Uterine tube dilation, immunotoxicity, high IgA	[194]
21.	In vitro (BeWo b30)	PS-NP, virgin	0.05–10, 80 µm, 100 mg/mL	Placental metabolism disruption	[195]
22.	In silico (human placenta)	PS-MP, virgin	1–100 nm	Inhibition of placental enzymes (sEH, UPRT, B3GAT1, SULT, NAT2, CYP1A1); highest toxicity: PC > PET > PS	[196]
23.	Mice (in vivo, oral)	PS-NP, virgin	10^2^ ng/L, 10^4^ ng/L, 10^6^ ng/L, throughout pregnancy	Metabolome changes (biotin/lysine/glycolysis)	[197]
24.	Ex vivo, human placental tissue	PS-NP, virgin	25 µg/mL	↑ IL-6, placental Fe homeostasis disruption	[198]
25.	In vivo (mice)	PS-MP	0, 0.5 mg/L, 5 mg/L, 50 mg/L, 35 days	Threat to male fertility	[199]
26.	Blood clam (*Tegillarca granosa*)	PS-MP, virgin	0.5, 5 µm, 0.069/0.69 mg/L	↓ motility, ATP, viability, DNA integrity	[200]
27.	Mice (in vivo, oral)	PS-NP, virgin	100 µg/L and 1000 µg/L, 90 days	Spermatogenesis disruption, ↓ hormones (IL-17A, dysbiosis)	[201]
28.	Mice (in vivo, oral), in vitro	PS-MP, PS-NP, virgin	5 µm, 80 nm, 40 mg/kg/day (60 days, mice), 10, 20, 40 µg/mL, 72 h	Pyruvate/thyroid hormone metabolism disruption, sperm damage	[202]
29.	Mice (in vivo)	PS-NP, virgin	25, 50, 100 nm, 28 days	Blood-testis barrier disruption → oligospermia/asthenospermia, inhibiting Prm3/Tnp1/Aurkc/Mea1/Mettl14 transcription and Pmfbp1/Ggn/Fsip2 expression	[204]
30.	Mice (in vivo, oral), in vitro (testicular cells)	PS-MP/NP, virgin	0.5/4/10 µm, 0.1 mL × 0.01% (~1 mg/day), 28 days	Penetration into Leydig/Sertoli cells, ↓ spermatogenesis/testosterone, inflammation	[207]
31.	Humans (testes/semen)	Various MP (PS/PE/PVC)	Concentration determination in samples	Presence of MP in male reproductive system	[208]
32.	Humans (semen, n = 40)	PS (31%), PE, PVC (8 polymers, 0.72–7.02 µm)	2 MP/sample	↓ progressive sperm motility	[209]

### 3.8. Urinary System

#### 3.8.1. Accumulation of MNPs in the Urinary System

A person can ingest tens of thousands of MNPs annually. They then penetrate the systemic circulation through the intestinal and lung epithelium and spread throughout the body, reaching the kidneys and bladder, among other organs [7,8].There is increasing literature data on MNP particle accumulation in the urinary system. Several studies have demonstrated that microplastics were detected in kidney biopsies, urine samples, and even bladder cancer tissues [210,211]. Review works emphasize that MNP accumulation in animal organs and tissues has been histologically recorded, while convincing evidence of the development of renal effects in humans is still insufficient [212]. Toxicity depends on particle size, dose, and duration of exposure (Table 9, Supplementary Materials Table S10) [213].

#### 3.8.2. Mechanisms of Cytotoxic Effects

**Figure 7:**
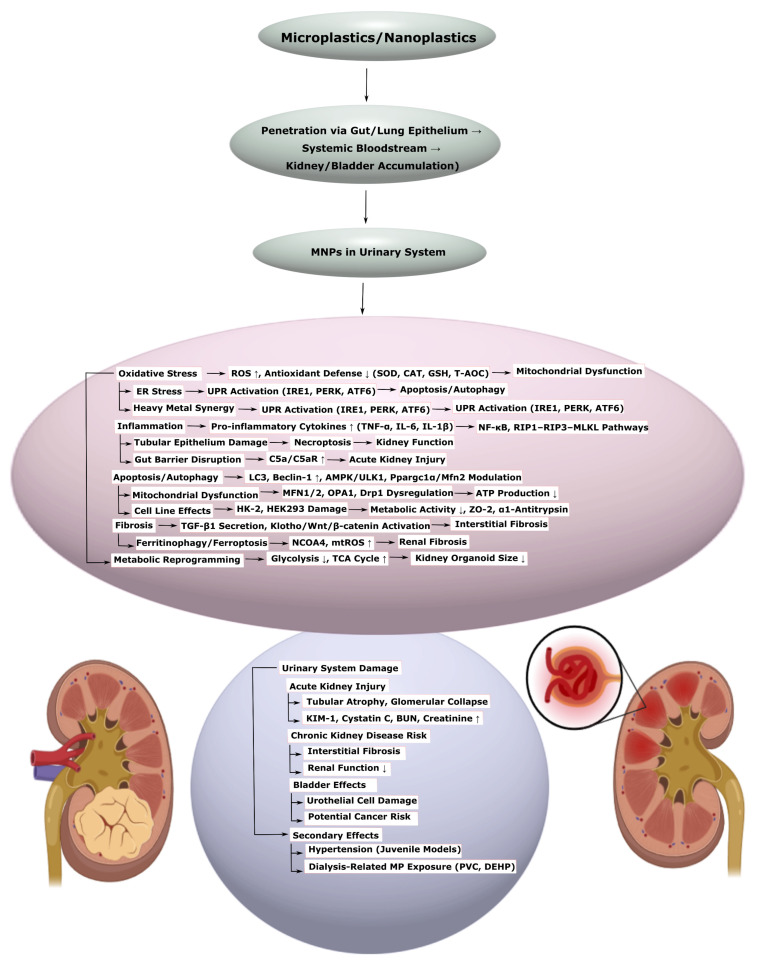
Conceptual diagram of the molecular mechanisms of microplastics and nanoplastics in the urinary system. The diagram illustrates particle uptake and penetration through the intestinal/lung epithelium, entry into systemic circulation, and accumulation in kidneys/bladder, triggering pathways of oxidative stress (ROS, SOD, CAT, GSH, T-AOC), inflammation (TNF-α, IL-6, IL-1β, NF-κB, RIP1–RIP3–MLKL), apoptosis/autophagy (LC3, Beclin-1, AMPK, ULK1, Ppargc1α, Mfn2), fibrosis (TGF-β1, Klotho, Wnt/β-catenin), and metabolic reprogramming (glycolysis, TCA cycle). Arrows (→) indicate process continuation, ↑ denotes increase, and ↓ denotes decrease. As a result, acute kidney injury, increased risk of chronic kidney disease, bladder damage, and hypertension may develop. Based on in vitro and in vivo studies. Abbreviation Key: ROS—Reactive oxygen species, SOD—Superoxide dismutase, CAT—Catalase, GSH—Glutathione, T-AOC—Total antioxidant capacity, ER—Endoplasmic reticulum, UPR—Unfolded protein response, IRE1—Inositol-requiring enzyme 1, PERK—Protein kinase R-like endoplasmic reticulum kinase, ATF6—Activating transcription factor 6, SLC7A11—Cystine/glutamate transporter, GPX4—Glutathione peroxidase 4, TNF-α—Tumor necrosis factor-α, IL-6—Interleukin-6, IL-1β—Interleukin-1β, NF-κB—Nuclear factor kappa B, RIP1—Receptor-interacting protein 1, RIP3—Receptor-interacting protein 3, MLKL—Mixed lineage kinase domain-like protein, C5a—Complement component 5a, C5aR—Complement 5a receptor, LC3—Microtubule-associated protein 1A/1B-light chain 3, Beclin-1—Autophagy-related protein, AMPK—AMP-activated protein kinase, ULK1—Unc-51-like autophagy-activating kinase 1, Ppargc1α—Peroxisome proliferator-activated receptor gamma coactivator 1-alpha, Mfn2—Mitofusin 2, MFN1—Mitofusin 1, OPA1—Optic atrophy 1, Drp1—Dynamin-related protein 1, HK-2—Human proximal tubule cell line, HEK293—Human embryonic kidney cell line, ZO-2—Zonula occludens-2, TGF-β1—Transforming growth factor-β1, Klotho—Klotho protein, Wnt—Wingless-related integration site, β-catenin—β-Catenin, NCOA4—Nuclear receptor coactivator 4, mtROS—Mitochondrial reactive oxygen species, TCA—Tricarboxylic acid cycle, KIM-1—Kidney injury molecule-1, BUN—Blood urea nitrogen, DEHP—Di(2-ethylhexyl)phthalate.

Kidney damage by MNP particles occurs via several key mechanisms. Oxidative stress develops as a result of an imbalance between the oxidant and antioxidant systems, with suppression of the latter and the formation of ROS, which then trigger the processes of apoptosis and autophagy [7]. OS induces the Unfolded Protein Response (UPR), resulting in the activation of mediators inositol-requiring kinase 1α(IRE1), protein kinase R-like ER kinase (PERK), and activating transcription factor 6 (ATF6) and the development of ER stress. The inflammatory process represents another response to MNP exposure: their particles induce excess ROS, increased pro-inflammatory cytokines TNF-α, IL-6, IL-1β, and others, and through the NF-κB and RIP1–RIP3–MLKL pathways, cause damage to renal tubular epithelial cells (Figure 7) [55].

MNP exposure triggers processes of apoptosis and cell death, realizing its action through increased LC3, Beclin-1, and modulation of the AMPK/ULK1 and Ppargc1α/Mfn2 pathways. Autophagy can be both adaptive and damaging, depending on the duration, type of MNP, and its quantity [25]. One mechanism is the development of mitochondrial dysfunction: damage to mitochondria, dysregulation of proteins responsible for their activity, such as MFN1/2, OPA1, Drp1, consequently reduced ATP production, energy deficiency, and kidney cell stress [31,214]. Feeding chickens PS-MPs caused mitochondrial damage, suppression of antioxidant system markers such as SOD, CAT, MDA, GSH, T-AOC, and activation of NF-κB, increased pro-inflammatory cytokines TNFα, iNOs, IL-1β, IL-6, and induction of necroptosis via the RIP1/RIP3/MLKL pathway. These data indicate the universality of inflammatory-degenerative changes in animal kidneys under the effect of PS-NPs and confirm the high risk of nephrotoxicity (Figure 7, Table 9, Supplementary Materials Table S10) [31].

Studies show that PS-NPs cause cytotoxicity in human embryonic kidney and liver cells, with particles sized less than 100 nm increasing cell mortality due to enhanced internalization, leading to mitochondrial damage. It is noted that enrichment of PS-NPs with heavy metals, such as Pb^2+^, enhances toxicity [215]. Experiments on cell models show that PS-NPs exhibit cytotoxic properties against human kidney proximal tubular epithelial cells (HK-2). PS-NPs were found to be dose-dependently absorbed by cultured HK-2 cells, causing mitochondrial dysfunction, increased levels of ROS, pro-inflammatory cytokines, ER stress (Figure 7), and activation of autophagy through the MAPK and AKT/mTOR signaling pathways (Table 9, Supplementary Materials Table S10) [216].

On human embryonic kidney cells (HEK 293) culture exposed to MPs at doses of 5 and 100 μg/mL, a reduction in their metabolic activity, proliferation, and progression of mitochondrial dysfunction was shown, which likely leads to negative consequences and causes reduced kidney function [217]. Also, on this model, but using PS-MPs at concentrations of 3–300 ng/mL, it was found that PS-MP particles first attach to the cell surface and then are absorbed by them, causing suppression of antioxidant defense through suppression of heme oxygenase-1, inflammation, and inducing apoptosis and autophagy in them. The action of PS-MPs causes suppression of zonula occludens-2 (ZO-2) and α1-antitrypsin proteins, which increases the risk of acute kidney injury and renal failure [218]. Studying the toxicity of di(2-ethylhexyl) phthalate (DEHP) and PS-MPs on HEK293 embryonic kidney cells showed increased expression of genes in the ROS/AMPK/ULK1 and Ppargc1α/Mfn2 signaling pathways and associated oxidative stress and cell autophagy (Figure 7, Table 9, Supplementary Materials Table S10) [25].

The combination of a high-fat diet (HFD) and PS-NPs revealed enhanced nephrotoxicity, manifested by increased blood urea nitrogen (BUN), creatinine, kidney injury molecule-1 (KIM-1), and cystatin C levels, as well as increased IL-1β, IL-6, TNF-α, and MDA with reduced SOD and GPx activity [219]. Co-exposure to Cd and PS-NPs synergistically increases iron accumulation in the kidneys, inducing ferroptosis and mitophagy through inhibition of the Nrf2 pathway, regulation of SLC7A11, GPX4, and increased LC3 expression [220]. Co-exposure to arsenic (As) and PS-NPs causes mitochondrial oxidative damage, ferritinophagy, and ferroptosis, mediated by NCOA4 and mtROS, leading to renal fibrosis (Figure 7, Table 9, Supplementary Materials Table S10) [221].

In experimental animal studies, MNPs also cause disruptions in renal structure and function. Thus, in experimental mice after 6 weeks of PS-NP consumption, a reduction in the “renal index,” tubular atrophy, glomerular collapse, and inflammatory tissue infiltration are noted, followed by reduced kidney function [222]. Moreover, in response to chronic PS-NP exposure, transforming growth factor β1 (TGF-β1) secretion and enhanced ferroptosis by tubular epithelial cells are observed, fibroblasts are activated, normal tissue is replaced by connective tissue, and interstitial renal fibrosis develops (Table 9, Supplementary Materials Table S10) [223].

Oral administration of PS-MPs leads to particle accumulation in the kidneys, histopathological changes in tubules and glomeruli, increased markers of ER stress, OS, inflammation, apoptosis/autophagy, and fibrosis [224]. In juvenile rat models, reduced weight gain and worsened kidney function indicators were observed, while antioxidants NAC and MitoTEMPO partially restored the effect (Table 9, Supplementary Materials Table S10) [216].

Co-exposure to PS-NPs and pharmaceutical residues, such as gentamicin, synergistically increases OS, NF-κB, TNF-α, IL-6, and Bax, caspase-3/9, significantly impairing kidney function [225]. PS-MPs carrying benzopyrene disrupt lipid metabolism and induce ferroptosis in vivo, but not in HK-2 cells, indicating cellular specificity [226]. In young rats, high doses of PS-MPs cause hypertension and kidney dysfunction, which are mitigated by sodium butyrate by reducing OS and modulating the gut microbiota [227]. In human kidney transplant tissues, polyethylene and polyvinyl chloride MPs accumulate at concentrations of 25.7–98.9 μg/g and 31.2–65.4 μg/g, with potential but statistically insignificant correlations with changes in blood pressure (Table 9, Supplementary Materials Table S10) [228].

Studies also showed that short-term (24 h) exposure to PS-NPs and PS-MPs in mouse kidneys led to necrotic, inflammatory, and infiltrative changes with the development of OS, detachment of tubular epithelium, loss of brush border, which manifested as kidney function impairment [229]. MPs induce inflammation, fibrosis, and epithelial–mesenchymal transformation of renal tubular epithelial cells in mice through activation of the Klotho/Wnt/β-catenin pathway and TGF-β1 secretion (Figure 7). Apparently, restoring Klotho function can slow the aging of epithelial cells and reduce fibroblast activity, which may seem promising for the development of a nephroprotective strategy (Table 9, Supplementary Materials Table S10) [230].

In the early stages of nephrogenesis, PS-MPs disrupt the development of human kidney organoids, adhering to nephron progenitor cells (NPCs), accumulating in glomerulus-like structures, and reducing organoid size through ROS-dependent apoptosis and suppression of Notch signaling, which impairs proximal and distal tubule formation [231]. Polypropylene MPs alter the renal lipidome, increasing ROS and disrupting triglyceride and phospholipid metabolism, leading to ultrastructural changes such as podocyte foot process effacement [232]. Polyethylene terephthalate NPs (PET-NPs) cause nephrotoxicity in mice, increasing BUN, creatinine, and MDA while reducing glutathione, with histopathological changes including glomerular hypotrophy and tubular degeneration. Betaine, an antioxidant, mitigates these effects, reducing OS and restoring kidney structure [233]. PS-MPs suppress glycolysis and enhance the activity of the tricarboxylic acid cycle, causing metabolic reprogramming and reduced kidney organoid size [234]. Single-cell RNA sequencing in mouse models with PS-MPs and HFD revealed changes in extracellular matrix organization, activation of PI3K-Akt, MAPK, and IL-17 pathways, enrichment of PF4+ macrophages, contributing to a profibrotic and protumorigenic microenvironment [235]. PS-MPs increase extracellular vesicle production in tubular cells, causing ER stress and fibrosis protein expression in fibroblasts (Figure 7, Table 9, Supplementary Materials Table S10) [236].

MPs can cause kidney damage not only directly through OS or inflammation in kidney tissue, but also through secondary mechanisms related, for example, to intestinal barrier disruption, which increases the functional load on the kidneys. This is followed by an increase in the level of the complement-activated product C5a in urine, increased expression of the C5aR receptor in the kidneys, and the development of acute kidney injury [237].

Collectively, the described MNP effects, upon long-term exposure, can lead to kidney damage, impaired filtration capacity, and increase the risk of chronic urinary system diseases [7]. Since MNPs are found in urothelial cells, hypotheses are put forward about the link between plastic and bladder cancer, as well as frequent recurrent urinary tract infections [211]. In patients receiving renal replacement therapy, the risk of contact with MPs particles through dialysis water and materials (PVC, DEHP) is higher, requiring further evaluation and monitoring (Table 9, Supplementary Materials Table S10) [238,239].

Most data are derived from animal and cell culture experiments; epidemiological data on this problem are insufficient. Accumulated preclinical and first clinical data indicate that MNPs are capable of accumulating in renal tissue, inducing mitochondrial dysfunction, ER–stress, inflammation, autophagy, and fibrosis; these processes are enhanced when combined with certain other compounds. Despite compelling mechanistic foundations for MNP effects, confirmation of a causal link in human populations requires large, standardized epidemiological and experimental studies. At the same time, reducing MNP exposure at the level of water supply, medical procedures, and consumer practices seems appropriate.

**Table 9:** Experimental studies on nephrotoxicity of micro- and nanoplastics: models, particle types, concentrations, and key effects (n = 27 primary sources). Arrows ↑ and ↓ denote increase and decrease, respectively. Abbreviations: MP—microplastics; NP—nanoplastics; PS-MP—polystyrene microplastic; PS-NP—polystyrene nanoplastic; PE—polyethylene; PP-MP—polypropylene microplastic; PET-NP—polyethylene terephthalate nanoplastic; PVC-MP—polyvinyl chloride microplastic; virgin—pristine/untreated plastic particles (no additives or aging); DEHP—di(2-ethylhexyl) phthalate; Cd—cadmium; As—arsenic; BaP—benzo[a]pyrene; HFD—high-fat diet; ROS—reactive oxygen species; AMPK—AMP-activated protein kinase; ULK1—Unc-51-like autophagy activating kinase 1; Ppargc1α—peroxisome proliferator-activated receptor gamma coactivator 1 alpha; Mfn2—mitofusin 2; SOD—superoxide dismutase; CAT—catalase; GSH—glutathione; T-AOC—total antioxidant capacity; MDA—malondialdehyde; NF-κB—nuclear factor kappa B; TNFα—tumor necrosis factor alpha; iNOS—inducible nitric oxide synthase; IL-1β—interleukin 1 beta; IL-6—interleukin 6; RIP1—receptor-interacting protein kinase 1; RIP3—receptor-interacting protein kinase 3; MLKL—mixed lineage kinase domain-like protein; HMOX1—heme oxygenase 1; GPX1—glutathione peroxidase 1; H_2_O_2_—hydrogen peroxide; mtDNA—mitochondrial DNA; MAPK—mitogen-activated protein kinase; AKT—protein kinase B; mTOR—mechanistic target of rapamycin; NAC—N-acetylcysteine; MitoTEMPO—mitochondria-targeted antioxidant; GAPDH—glyceraldehyde-3-phosphate dehydrogenase; SOD2—superoxide dismutase 2; HO-1—heme oxygenase 1; NLRP3—NOD-like receptor family pyrin domain containing 3; ZO-2—zonula occludens-2; BUN—blood urea nitrogen; KIM-1—kidney injury molecule 1; GPx—glutathione peroxidase; Nrf2—nuclear factor erythroid 2-related factor 2; SLC7A11—solute carrier family 7 member 11; GPX4—glutathione peroxidase 4; LC3—microtubule-associated protein 1 light chain 3; NCOA4—nuclear receptor coactivator 4; mtROS—mitochondrial ROS; TGF-β1—transforming growth factor beta 1; ER—endoplasmic reticulum; OS—oxidative stress; Bax—Bcl-2-associated X protein; Klotho—anti-aging protein Klotho; Wnt—Wingless/Integrated; EMT—epithelial–mesenchymal transition; NPC—nephron progenitor cells; Notch—Notch signaling pathway; TG—triglycerides; PL—phospholipids; TCA—tricarboxylic acid (cycle); ECM—extracellular matrix; PI3K-Akt—phosphoinositide 3-kinase/protein kinase B; IL-17—interleukin 17; PF4—platelet factor 4; EVs—extracellular vesicles; CD63—cluster of differentiation 63; CD81—cluster of differentiation 81; NTA—nanoparticle tracking analysis; TEM—transmission electron microscopy; IF—immunofluorescence; IHC—immunohistochemistry; p-EIF2α—phosphorylated eukaryotic initiation factor 2 alpha; p-IRE1α—phosphorylated inositol-requiring enzyme 1 alpha; ATF6—activating transcription factor 6; PAI-1—plasminogen activator inhibitor 1; CTGF—connective tissue growth factor; C5a—complement component 5a; C5aR—C5a receptor; AKI—acute kidney injury; HEK293—human embryonic kidney 293 cells; HK-2—human kidney proximal tubular cells; NRK-49F—normal rat kidney fibroblasts; NIH/3T3—mouse embryonic fibroblasts; scRNA-seq—single-cell RNA sequencing.

№	Experimental Model	Type and Source of MP/NP	Concentrations	Results/Observations	Reference
1.	In vitro (HEK293) + DEHP	PS-MP + DEHP	5 µm, MP: 200 mg/kg/day; DEHP: 200 mg/kg/day; combo: 200 each	↑ ROS/AMPK/ULK1, Ppargc1α/Mfn2; oxidative stress, autophagy	[25]
2.	Chickens (in vivo, oral)	PS-MP, virgin	30 nm, 1–100 mg/L feed (6 weeks)	Mitochondrial damage, ↓ SOD/CAT/GSH/T-AOC, ↑ MDA; ↑ NF-κB/TNFα/iNOS/IL-1β/IL-6; necroptosis (RIP1/RIP3/MLKL)	[31]
3.	Humans, in vivo (native tissues/biological fluids)	PE and PS	Concentration determination in samples	Presence/deposition in kidneys and urine; first evidence in human kidneys	[210]
4.	Caco-2 (in vitro, 24 h)	PS-MP, virgin	Spheres 200 nm; 2 µm, 0, 10, 100 µg/mL	↑ uptake (max 200 nm), ↑ HMOX1/CAT/GPX1, ↓ H_2_O_2_, ↑ mtDNA/morphology; particle number-dependent effect	[213]
5.	HK-2 (in vitro), mice (in vivo, oral)	PS-NP, virgin	2 µm PS-MP; 0.025–0.8 µg/mL (in vitro, 0–120 min); 0.2–0.4 mg/day (in vivo, 4–8 weeks)	Dose-dependent uptake, ↑ ROS, cytokines, ER stress, autophagy (MAPK/AKT/mTOR); body weight gain, renal dysfunction; NAC/MitoTEMPO partially restore	[216]
6.	Human embryonic kidney cells (HEK293) and human hepatocellular liver cells (Hep G2)	PS-MP, virgin	1 µm, 100 µg/mL, 24, 48, 72 h	↑ viability, significant morphological changes in liver/kidney cells, ↑ ROS, ↓ GAPDH, SOD2, CAT	[217]
7.	HEK293 cells (human embryonic kidney)	Round polystyrene MP (PSMP), 3.54 ± 0.39 µm	3 ng/mL (non-cytotoxic); 300 ng/mL (cytotoxic)	Membrane adhesion and full cellular uptake, oxidative stress, HO-1 inhibition, apoptosis + autophagy, ↑ inflammation,↓NLRP3, ↓ ZO-2, α1-antitrypsin	[218]
8.	Mice (in vivo, oral) + HFD	PS-NP, virgin	100 nm, 25 mg/kg/day	↑ BUN/creatinine/KIM-1/cystatin C/IL-1β/IL-6/TNF-α/MDA; ↓ SOD/GPx	[219]
9.	Mice (in vivo, oral) + Cd	PS-NP + Cd	300 nm PSNPs (1 mg/kg) + Cd (1.5 mg/kg) (35 days)	↑ Fe, ferroptosis/mitophagy (↓ Nrf2/SLC7A11/GPX4,↑LC3)	[220]
10.	Mice (in vivo, oral) + As	PS-NP + As	0.5 ppm PSNPs + 5 ppm arsenic (60 days)	Mitochondrial damage, ferritinophagy/ferroptosis (NCOA4/mtROS), fibrosis	[221]
11.	Mice (in vivo, oral)	PS-NP, virgin	100 nm, 6 weeks	↓ renal index, tubular atrophy, glomerular collapse, inflammation	[222]
12.	In vitro (HK-2, NRK-49F)	PS-NP, virgin	60 µg/mL	↑ TGF-β1, epithelial ferroptosis, fibroblast activation, interstitial fibrosis	[223]
13.	Mice (in vivo, oral)	PS-MP, virgin	1000 nm, 2.0 mg/kg/day, 28 days	Accumulation, histopathology, ↑ ER/OS/inflammation/apoptosis/autophagy/fibrosis	[224]
14.	Mice (in vivo, oral) + gentamicin	PS-NP + gentamicin	100 mg/kg/day (15 days)	↑ OS/NF-κB/TNF-α/IL-6/Bax/caspases-3/9; synergistic dysfunction	[225]
15.	HK-2/in vivo + BaP	PS-MP + BaP	100 nm PS-MP; 400 µg/mL (56 days); BaP—4 µM/L (48 h)	Lipid disruption, ferroptosis in vivo (not in HK-2)	[226]
16.	Young rats (in vivo, oral)	PS-MP, virgin	10 mg/L and sodium butyrate (400 mg/kg/day), 12 weeks	Hypertension, renal dysfunction; sodium butyrate ↓ OS, modulates microbiota	[227]
17.	Humans (kidney transplants)	PE/PVC-MP	25.7–98.9 µg/g PE; 31.2–65.4 µg/g PVC	Accumulation; correlation with BP (non-significant)	[228]
18.	Mice (in vivo, oral)	PS-NP/MP, virgin	PS-NP (50 nm) and PS-MP (300 nm, 600 nm, 4 µm), 0.1 mg/day (0.1 mL × 1 mg/mL)	Necrosis, inflammation, infiltration, OS, epithelial detachment, loss of brush border	[229]
19.	Mice (in vivo, oral)	PS-MP, virgin	5 µm, 28 days	Inflammation/fibrosis/EMT (Klotho/Wnt/β-catenin, TGF-β1); Klotho slows progression	[230]
20.	Human kidney organoids (in vitro)	PS-MP, virgin	1 µm	Adhesion to NPC, glomerular accumulation,↓size, ROS-apoptosis, ↓ Notch; tubular disruption	[231]
21.	Mice (in vivo, oral)	PP-MP (polypropylene), virgin	<5 µm PP-MP; 100–1000 µg/L (7 or 42 days)	Lipidome changes, ↑ ROS, TG/PL disruption, podocyte effacement	[232]
22.	Mice (in vivo, oral)	PET-NP (polyethylene terephthalate), virgin	200 mg/kg daily, 30 days	↑ BUN/creatinine/MDA, ↓ GSH; glomerular hypotrophy, tubular degeneration; betaine mitigates	[233]
23.	Kidney organoids (in vitro)	PS-MP, virgin	1 µm	↓ glycolysis, ↑ TCA cycle, metabolic reprogramming, ↓ size	[234]
24.	Mice (in vivo, oral) + HFD, scRNA-seq	PS-MP, virgin	1 µm, 10 mg/L + HFD (18 weeks)	ECM changes, ↑ PI3K-Akt/MAPK/IL-17, PF4^+^ macrophages, profibrotic/protumorigenic environment	[235]
25.	HK-2 (in vitro, conditioned medium on NIH/3T3 fibroblasts), in vivo (C57BL/6 mice, oral)	PS-MP, virgin	In vitro: 0.4–0.8 mg/mL (24–48 h); in vivo: 0.2–0.4 mg/day	↑ exosomes/EVs (CD63^+^, CD81^+^, NTA, TEM, IF, IHC), ↑ ER stress (p-EIF2α ↑, p-IRE1α ↑, ATF6 ↑), ↑ fibrotic markers (collagen 1 ↑, PAI-1 ↑, CTGF ↑), ↑ ROS (in vitro/in vivo)	[236]
26.	Mice (in vivo, oral)	PS-MP, virgin	0.5 mg/kg/day (L-MP), 2 mg/kg/day (H-MP) (8 weeks)	Intestinal barrier disruption, ↑ C5a in urine, ↑ C5aR in kidneys, AKI	[237]
27.	Hemodialysis patients, field study (London)	MP/NP	300–600 L water/week; filtration 99% → pass-through: 0.0021–3.768 particles/week	↑ MP/NP exposure risk via dialysate; sources: tap water → dialysis system; potential accumulation in blood/kidneys (estimated)	[238]

### 3.9. Gastrointestinal Tract

#### 3.9.1. Routes of Penetration, Absorption, Accumulation, and Transport of MNPs in the GIT

**Figure 8:**
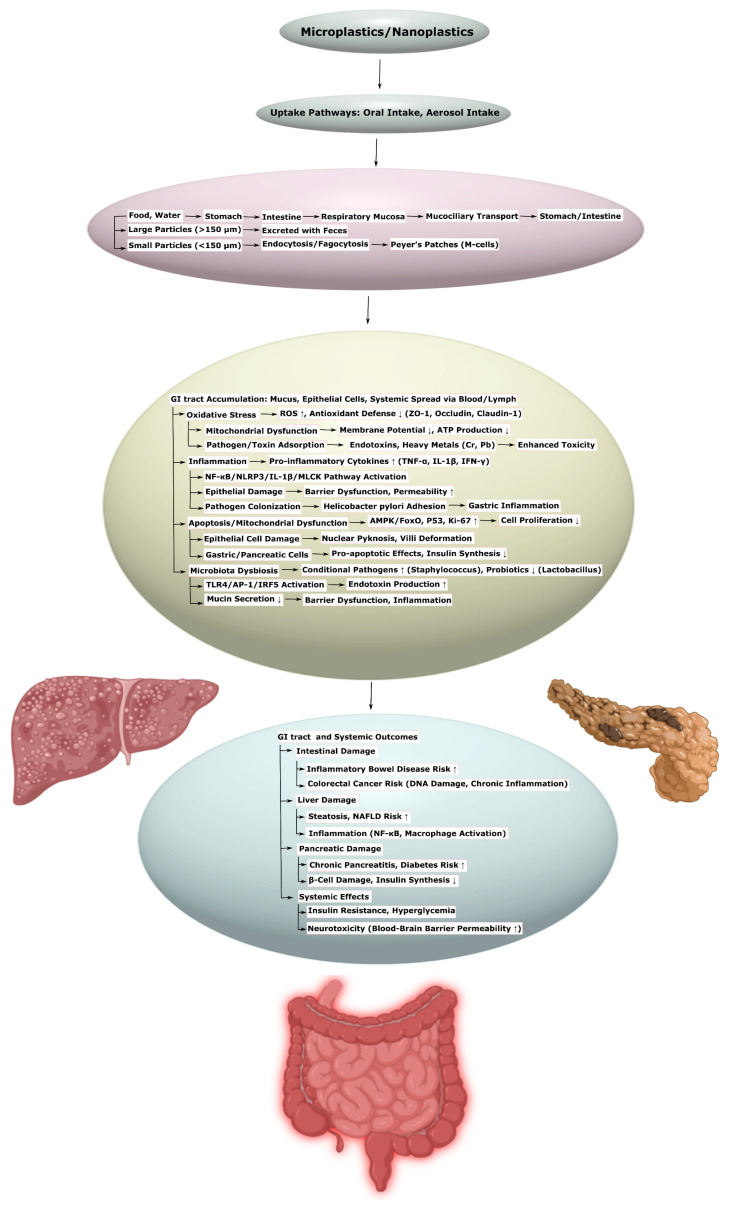
Conceptual diagram of the molecular mechanisms of microplastics and nanoplastics in the gastrointestinal tract. The diagram illustrates particle uptake through the intestine or respiratory tract, their accumulation in the mucosal layer and epithelial cells, and systemic spread via blood/lymph, inducing oxidative stress (ROS ↑, ZO-1 ↓, Occludin ↓, Claudin-1 ↓), inflammation (TNF-α ↑, IL-1β ↑, IFN-γ ↑, NF-κB/NLRP3/MLCK ↑), apoptosis/mitochondrial dysfunction (AMPK/FoxO ↑, P53 ↑, Ki-67 ↑), and microbiota dysbiosis (Staphylococcus ↑, Lactobacillus ↓). Arrows (→) indicate process continuation, ↑ denotes increase, and ↓ denotes decrease. The outcomes include intestinal damage, increased risk of inflammatory bowel disease, colorectal cancer, liver damage (steatosis, NAFLD), pancreatic damage (chronic pancreatitis, diabetes), and systemic effects (insulin resistance, hyperglycemia, neurotoxicity). Based on in vitro and in vivo studies. Abbreviation Key: MPs—Microplastics, NPs—Nanoplastics, ZO-1—Zonula occludens-1, Occludin—Occludin, Claudin-1—Claudin-1, Cr—Chromium, Pb—Lead, TNF-α—Tumor necrosis factor-α, IL-1β—Interleukin-1β, IFN-γ—Interferon-gamma, NF-κB—Nuclear factor kappa B, NLRP3—NOD-like receptor pyrin domain-containing 3, MLCK—Myosin light-chain kinase, AMPK—AMP-activated protein kinase, FoxO—Forkhead box O transcription factor family, p53—Tumor suppressor protein p53, Ki-67—Cell proliferation marker, TLR4—Toll-like receptor 4, AP-1—Activator protein 1, IRF5—Interferon regulatory factor 5, NAFLD—Non-alcoholic fatty liver disease.

The GIT is the main route of MNP entry into the human body. Once in the stomach and intestines, MNPs can exert toxic effects on tissues, however the mechanisms of their action are not yet fully understood [240]. Reviews note that in human and animal tissues, MNPs can cause oxidative stress, inflammation, cellular stress, and even apoptosis [241]. A study showed that MPs particles can accumulate in the digestive tract and disrupt the gut microbiota, causing not only dysbiosis but also inflammation, metabolic disorders, and various diseases (Figure 8) [242].

The main route of entry is oral. MNPs are present in food, water, and air. Some aerosol MNPs settle on the respiratory tract mucosa, from where they enter the stomach and intestines via mucociliary transport [243]. Small (<2.5 μm) particles can penetrate the bloodstream through the bronchial epithelium, while larger ones are transported by tracheal cilia into the gastrointestinal tract. After entering the stomach, MNP particles encounter gastric juice and salts, but the strength and chemical inertness of the polymers prevent their digestion, so larger particles are eliminated from the body in stool, and only a small portion accumulates on the mucosal surface (Table 10, Supplementary Materials Table S11) [241]. MNPs sized <150 μm are capable of penetrating the intestinal epithelium, entering the lymphatic and circulatory systems and subsequently spreading throughout the body. This process is realized by endocytosis by enterocytes, phagocytosis by phagocytes and non-phagocytic cells, and also due to a strong electrostatic interaction between positively charged MNPs and the cell plasma membrane. Peyer’s patches, rich in M-cells, likely serve as the main gateway for MNPs: in them, barrier microvilli are absent, which facilitates particle uptake (Figure 8) [244].

Through the lymphatic system and bloodstream, MNPs are transported to other organs. Animal experiments have shown MNP accumulation in the liver, kidneys, spleen, lungs, brain, and even the placenta in pregnant women [114]. Specifically, it is reported that upon oral administration of nanoparticles, they are found in the thick intestine, liver, pancreas, and other organs of mice in just a few days [245]. In the intestine, MNPs accumulate in mucus and epithelial cells and disrupt barrier function. Feeding mice PS-MPs was shown to reduce the level of ZO-1, occludin (OCLN), and claudin-1 (CLDN-1) proteins, damaging epithelial cells and disrupting their function (Figure 8, Table 10, Supplementary Materials Table S11) [53]. Also, MNPs adsorb microorganisms, as well as contaminants, and deliver them to the body, which enhances their toxicity, as endotoxins and carcinogens enter the body along with them [246].

#### 3.9.2. Mechanisms of MNP Toxic Action on GIT Organs

The key mechanisms of MNP cytotoxic action following particle uptake by the cell consist of the development of oxidative stress, inflammation, mitochondrial dysfunction, and apoptosis. At the same time, MNPs are capable of influencing the composition of the GIT microbiota.

MNPs promote the adhesion and accelerated colonization of Helicobacter pylori on the gastric mucosa with subsequent development of the inflammatory process and cell damage [247]. An average of 9.4 MPs per person, predominantly as fibers, were found in human gastric contents, confirming their pervasive spread and potential interaction with pathogens [248]. In gastric cell cultures, NPs showed cytotoxicity: they reduced epithelial cell proliferation and demonstrated a pro-apoptotic potential (Figure 8) [249]. The latter may be due to their additive properties, particularly the ability to carry pathogens and toxins from the external environment into the body on their surface. For example, a study using a dynamic GIT simulator showed that 23.11% of chromium and 23.17% of lead from polyethylene and polypropylene MPs overcome the intestinal absorption barrier, with release depending on pH and polymer properties (Table 10, Supplementary Materials Table S11) [250].

In the liver, MNPs induce metabolic and inflammatory changes: increased blood glucose level and insulin resistance, reduced cholesterol and triglycerides in the liver [61]. Simultaneously, inflammation develops in liver cells through increased expression of pro-inflammatory cytokines, activation of the NF-κB signaling pathway [251], and secondary activation of hepatic macrophages by endotoxins produced by the altered gut microbiota [252]. MPs found in cirrhotic liver include six types of polymers ranging from 4 to 30 μm in size, unlike healthy tissues, suggesting a role for cirrhosis in their accumulation [253]. The combination of ethanol and polystyrene MP enhances particle accumulation in the liver and intestine, exacerbating steatosis and mucosal damage [254]. In aged mice, polystyrene MP with a gastrointestinal corona activates the AMPK/FoxO pathway, causing oxidative stress and inflammation in the liver [255]. Furthermore, MNPs may contain various toxic substances and pollutants on their surface that are themselves hepatotoxic. The developing changes in liver cells increase the risks of liver damage and the development of NAFLD (Figure 8) [56]. Low-dose polyethylene terephthalate MP exposure is noted not to cause pathological changes but affects immune cell transcriptome and microbiota metabolism (Table 10, Supplementary Materials Table S11) [256].

MNPs can exert both direct and indirect effects on the pancreas, causing enhanced local inflammation in its tissues [257]. PET-MPs can change the pancreatic transcriptome, triggering genetic programs typical of autoimmune inflammation and diabetes. They increase the expression of inflammation markers, accompanied by oxidative stress. Available data suggest that MNPs can reduce insulin synthesis by damaging β-cells and increase the risk of chronic pancreatitis (Figure 8) [258].

Mechanisms of intestinal damage by MNP particles are multifactorial. MPs physically irritate the epithelium and disrupt its barrier function through oxidative stress, cell infiltration, inflammation, increased epithelial permeability, and reduced expression of tight junction proteins. Activation of the NF-κB/NLRP3/IL-1β/MLCK pathway increases ROS formation and triggers the inflammatory cascade, increasing levels of TNFα, IL-1β, IFNγ, and MLCK, which leads to epithelial cell contraction and weakened intestinal barrier function [53,54]. Polystyrene nanospheres penetrate intestinal epithelial cells more effectively than microparticles, causing damage to membranes and moderate oxidative stress [259]. Polyethylene MP induces nuclear pyknosis, villus deformation, and goblet cell hyperplasia in the mouse ileum, with increased expression of P53 and Ki-67 [260]. Polystyrene MP disrupts intestinal homeostasis by activating the Notch pathway, which exacerbates colitis and liver inflammation [261]. In rats, polyethylene MP reduces mucin secretion and expression of occludin, ZO-1, and claudin-1, enhancing inflammation and apoptosis [262]. Polypropylene MP causes apoptosis and oxidative stress in the colon through TLR4/NF-κB activation (Figure 8, Table 10, Supplementary Materials Table S11) [263].

Secondly, MNPs cause dysbiosis and increase the growth of opportunistic bacteria, reducing the diversity of the normal flora [56,57]. High doses of polyethylene MP increase the abundance of Staphylococcus and reduce Parabacteroides, causing inflammation through activation of TLR4, AP-1, and IRF5 [264]. Polystyrene MP suppresses the growth of probiotics, such as Lactobacillus, contributing to dysbacteriosis [265]. In mice, polystyrene MP reduces the expression of tight junction genes and causes dysbiosis, especially in females [266]. The dysbiotic microbiome further produces endotoxins and toxic metabolites, enhancing mucosal inflammation and systemic immune response. Microorganisms can also use MNPs as a shuttle to penetrate the intestine and release toxins there, potentiating carcinogenesis [267]. Increased MP content in the feces of inflammatory bowel disease (IBD) patients correlates with disease severity (Table 10, Supplementary Materials Table S11) [58].

A summary of 30 in vitro studies showed that MNPs cause various effects depending on particle size. Small particles easily penetrate cells and are more often localized near the nucleus, while larger particles are retained on the membrane [240]. Depletion of antioxidants and enhanced synthesis of pro-inflammatory signaling molecules are observed in cells [268]. Disruptions of mitochondrial functions were also found: changes in membrane potential, increased ROS formation, and elevated markers of apoptosis [269,270]. At high NP concentrations, reduced cell viability and proliferation, and sometimes destruction of cell membranes, were found (Figure 8, Table 10, Supplementary Materials Table S11) [271].

Studies also showed that fish fed with MPs of different sizes accumulated particles in the gastrointestinal tract. Inflammatory and structural changes in the intestinal mucosa were found in sea bass when PVC plastic was included in the diet. In Danio rerio fish, PS-NPs sized 5 μm accumulated in the intestine, gills, and liver, causing pathological changes and increased markers of oxidative stress (Figure 8) [244]. In red mullet and hake, MPs correlate with increased cytokines and antioxidant enzymes, indicating inflammation (Table 10, Supplementary Materials Table S11) [272].

In experiments on mice and rats with oral administration of PS-MPs over several weeks, intestinal and systemic effects are observed. Long-term exposure caused disruption of the intestinal barrier, low antioxidant activity, and mild inflammation in the large intestine of mice. This was accompanied by changes in the microbiota, in particular, an increase in the number of opportunistic pathogenic bacteria. In mice pre-fed MPs, cyclophosphamide administration caused a more pronounced hepatotoxic effect compared to those without MPs in the diet. This correlated with changes in the intestinal microflora [56]. Additionally, polystyrene MP reduces lipid digestion by forming heteroaggregates with lipids and adsorbing lipase [273]. Melatonin, in turn, mitigates the toxic effect of polyethylene MP, restoring mucin secretion and intestinal tight junctions (Table 10, Supplementary Materials Table S11) [262].

In the induction of acute pancreatitis in mice, preliminary oral feeding of PS-MPs enhanced the severity of inflammation in the pancreas [257]. A high dose of MPs led to greater intestinal barrier dysfunction, which exacerbated the course of pancreatitis. A recently published study in piglets showed that the use of PET-MPs in their diet caused specific changes in the pancreas. High doses of PET-MPs disrupted the expression of 86 genes, caused oxidative stress and an immune response similar to that in diabetes (Table 10, Supplementary Materials Table S11) [258].

Short-term exposure to composite MPs causes inflammation and dysbacteriosis, reducing the abundance of Alistipes, which is linked to obesity risk [274]. A high-fat diet combined with polystyrene MP increases intestinal permeability, increases inflammatory cells, and disrupts glucose and lipid metabolism [275]. Available data suggest the ability of PS-NPs particles to induce lipophagy in hepatocytes via the AMPK/ULK1 pathway and potentiate lipid accumulation in them. Subsequently, the body attempts to compensate for the loss of lipid reserves by using glucose, and insulin resistance and hyperglycemia develop. The result of this process may be the development of NAFLD and its subsequent transformation into fibrosis (Figure 8) [60].

The consequences of chronic MNP exposure for humans are not fully established. Intestinal barrier dysfunction and dysbiosis may increase the risk of IBD by sustaining the inflammatory response and disrupting epithelial regeneration [59]. Experimentally, MNPs were shown to cause insulin resistance, increased blood glucose [241], and increased risk of NAFLD and diabetes [61]. Probably, in humans, long-term MNP load may increase the risk of diabetes, obesity, and metabolic syndrome. In patients predisposed to pancreatitis, MNPs may accelerate its development, increase the frequency of exacerbations, and cause endocrine dysfunction [257]. The growth of opportunistic microflora in the intestine under the influence of MNPs leads to activation of the immune system by circulating pathogen-associated molecular patterns, inflammation, and disruption of blood–brain barrier permeability. This facilitates MNP penetration into the brain and induces neurotoxicity, neuroinflammation, and likely increases the risk of Alzheimer’s disease and Parkinson’s disease [276]. Increased MNP content was found in the tumor tissues of the large intestine of colorectal cancer patients, which may indicate a potential carcinogenic effect (Table 10, Supplementary Materials Table S11) [277]. Mechanisms include direct DNA damage, chronic inflammation, and oxidative stress, which create a background for mutations [246]. Consequently, long-term MNP exposure to the human body may increase the risk of colorectal cancer. At the same time, Escherichia coli can use MNP particles to deliver carcinogenic toxins to the colonic epithelium, thereby increasing the risk of colorectal cancer (Figure 8) [267].

In general, the clinical consequences of MNP exposure are difficult to predict. However, available data demonstrate a uniformity of mechanisms for MNP particle delivery to the body, their penetration into cells, organs, and biological fluids, and the effects they cause. At the same time, we observe a wide range of in vitro and in vivo studies and a small number of works devoted to studying the impact of microplastics on the human body and health, which is of scientific interest.

**Table 10:** Experimental studies on gastrointestinal toxicity of MNPs: models, particle types, concentrations, and key effects (n = 36 primary sources). Arrows (→) indicate process continuation, ↑ denotes increase, and ↓ denotes decrease. Abbreviations: MNPs—micro- and nanoplastics; MP—microplastics; NP—nanoplastics; PS-MP—polystyrene microplastic; PS-NP—polystyrene nanoplastic; PE-MP—polyethylene microplastic; PP-MP—polypropylene microplastic; PET-MP—polyethylene terephthalate microplastic; PC—polycarbonate; PETF—polyethylene terephthalate fiber; PA—polyamide; virgin—pristine/untreated plastic particles (no additives or aging); PS-COOH—carboxylated polystyrene; ROS—reactive oxygen species; ZO-1—zonula occludens-1; OCLN—occludin; CLDN-1—claudin-1; NF-κB—nuclear factor kappa B; NLRP3—NOD-like receptor family pyrin domain containing 3; MLCK—myosin light-chain kinase; NAC—N-acetylcysteine; MCC950—NLRP3 inhibitor; ML-7—MLCK inhibitor; TJ—tight junction; IL-1β—interleukin 1 beta; CTX—cyclophosphamide; FMT—fecal microbiota transplantation; IBD—inflammatory bowel disease; dm—dry matter; DSS—dextran sulfate sodium; LD—lipid droplets; AMPK—AMP-activated protein kinase; ULK1—Unc-51-like autophagy activating kinase 1; HFD—high-fat diet; GO—Gene Ontology; CC—cellular component; BP—biological process; MF—molecular function; MAPK—mitogen-activated protein kinase; TLR—Toll-like receptor; qPCR—quantitative polymerase chain reaction; ERK—extracellular signal-regulated kinase; MER—likely MEK/ERK-related; CDK4—cyclin-dependent kinase 4; TRPV1—transient receptor potential vanilloid 1; iNOS—inducible nitric oxide synthase; IL-8—interleukin 8; MNPL—micro/nanoplastics; HCS—high-content screening; ECIS—electric cell–substrate impedance sensing; LDH—lactate dehydrogenase; MTT—3-(4,5-dimethylthiazol-2-yl)-2,5-diphenyltetrazolium bromide; DCFH-DA—2′,7′-dichlorofluorescin diacetate; DHE—dihydroethidium; MitoSOX—mitochondrial superoxide indicator; GI—gastrointestinal; Cr—chromium; Pb—lead; AP-1—activator protein 1; IRF5—interferon regulatory factor 5; DEGs—differentially expressed genes.

№	Model	Type of MP/NP	Dose/Duration	Key Results	Reference
1.	C57BL/6J mice (in vivo, oral, 28 days) + Caco-2 cells (in vitro)	PS-MP, 0.2/1/5 µm	1 mg/kg/day (in vivo); PS5—in vitro	↑ ROS, inflammation, cytokines, ↑ permeability, ↓ mucus, ↓ ZO-1, OCLN, CLDN-1 (5 µm > 1 > 0.2), PS5 → ↑ NF-κB/NLRP3/MLCK; NAC/MCC950/ML-7: ↓ ROS, inflammation, ↑ TJ proteins; Mechanism: ROS → NF-κB/NLRP3/IL-1β/MLCK	[53]
2.	Mice + CTX + FMT	PS-MP (virgin)	18/180 µg/kg/day, mice (drinking, 90 days) + CTX (80 mg/kg i.p.) + FMT	MP + CTX → ↑ hepatotoxicity, ↑ intestinal permeability, ↓ antioxidants, dysbiosis; Dysbiosis ↔ ↑ hepatotoxicity; FMT from MP-mice → ↑ liver sensitivity to CTX; MP risk revealed with xenobiotics	[56]
3.	Mice (lactation) + FMT + mouse mammary epithelial cells (EpH4)	PS-MP (virgin)	0, 0.15, 0.3 or 0.6 mg/mL, 48 h (in vitro); 3 mg/L, 30 mg/L (in vivo)	↑ intestinal permeability, colon inflammation, dysbiosis? Accumulation in mammary gland → inflammation, ↑ lipid metabolism, ferroptosis; FMT → ↑ blood-milk barrier permeability, inflammation; gut–mammary axis; microbiota role	[57]
4.	Feces from IBD patients and healthy controls	15 types MP (PET 22.3–34.0%; PA 8.9–12.4%; sheets/fibers)	IBD: 41.8 particles/g dm; healthy: 28.0 particles/g dm	↑ MP in IBD vs. healthy, IBD severity; sources: plastic food/water packaging, dust; IBD ↔ MP retention (or pathogenesis contribution); fecal MP—exposure biomarker	[58]
5.	C57BL/6 mice (healthy + DSS-colitis), oral, 6 weeks	Polystyrene MP	5 µm, 2.3 mg/kg/day, 6 weeks	Healthy: ↑ endocrine cells, ↑ highly sulfated mucins, ↑ lamina propria cells, ↓ macrophages; Colitis: ↑ severity (ulcers, inflammation), ↓ neutral mucins; MP–IBD link	[59]
6.	Human hepatocytes (in vitro)	Polystyrene NP (PSNP)	50, 100, 200 nm	Internalization → ↓ viability, ↑ LD; autophagy inhibition → ↑ LD; lipophagy activation (LD in autophagosomes) →flux block (↓ lysosomes); Pathway: AMPK/ULK1; ↓ AMPK → ↑ lipids; Mechanism: lipophagy → block → lipid accumulation	[60]
7.	Mice (environmentally relevant dose) + multiomics	MP	1 µm, 10^6^ particles/day, 1–2 weeks	↑ fasting glucose/insulin, gut–liver axis disruption → insulin resistance, diabetes risk; Mechanism: metabolomics + microbiomics; Recommendation: cohort studies	[61]
8.	8 healthy volunteers (Europe/Asia) + diet diary	9 types MP (PP, PETF—predominant)	50–500 µm, 20 particles/10 g feces	100% positive samples, unintentional ingestion; sources: food/water	[243]
9.	In vitro (gastric cells) + *H. pylori*	PS-MP (virgin)	100–150 µm	↑ *H. pylori* adhesion/colonization, inflammation	[247]
10.	Humans (gastric aspirate)	MP (fibers)	9.4 particles/person, estimated MP intake 32.2 particles/day	Ubiquitous presence in stomach	[248]
11.	In vitro (GES-1 gastric cells), in vivo (mice)	NPs (various)	60 nm	↓ proliferation, ↑ apoptosis, RhoA/F-actin signaling activation	[249]
12.	In vitro (SimuGIT), in vivo (mice, typical)	PE/PP-MP	0.625 mg/mL (in vitro), 4 h; 0.01–1 mg/kg/day (in vivo), 4–12 weeks	Release of 23.11% Cr, 23.17% Pb	[250]
13.	Mice, oral	PS-MP (virgin)	0.5 µm, 0.5 mg/day, 4 weeks	↑ NF-κB, pro-inflammatory cytokines in liver	[251]
14.	Humans (liver biopsy, cirrhosis)	MP (6 polymers)	4–30 µm	Accumulation only in cirrhotic liver	[253]
15.	Mice + ethanol	PS-MP (virgin)	0.1 mg/kg, 5 weeks	↑ steatosis, intestinal mucosa damage	[254]
16.	Aged mice	PS-MP (with GI corona)	1 × 10^3^ to 1 × 10^12^ particles/L, 10 days	↑ AMPK/FoxO, ↑ ROS and liver inflammation	[255]
17.	Mice, oral	PET-MP (virgin)	3 × 10^4^ particles/mouse every 3 days (in 100 µL PBS), 8 weeks	No histopathology, but transcriptome and microbiota changes	[256]
18.	Mice + induced pancreatitis	PS-MP (virgin)	100 and 1000 µg/L, 28 days	Enhanced pancreatic inflammation	[257]
19.	Piglets (diet)	PET-MP (virgin)	0.1 g/day or high 1 g/day, 4 weeks	↑ 86 genes, ↑ ROS, diabetes-like response	[258]
20.	In vitro (Caco-2 cells)	PS-NP vs. PS-MP	0.1, 0.5, 1, 5 µm, 500 µg/mL, 24 h	NP penetrate > MP, ↑ ROS, membrane damage	[259]
21.	Mice, oral	PE-MP (virgin)	6, 60, 600 µg/mL/day, 15 days	Nuclear pyknosis, villus deformation, ↑ P53/Ki-67	[260]
22.	Mice, oral	PS-MP (virgin)	5 µm (100 µg/L, 42 days)	↑ Notch → colitis, liver inflammation	[261]
23.	Rats, oral	PE-MP (virgin)	4.0–6.0 µm, 3.75 or 15 mg/kg/day, 5 weeks	↓ mucin/ZO-1/occludin/claudin-1, ↑ apoptosis; melatonin restores	[262]
24.	Rats, oral	PP-MP (virgin)	8 and 70 µm; 0.1, 1.0, 10 mg/mL, 28 days	↑ TLR4/NF-κB → apoptosis, ↑ ROS	[263]
25.	Mice, oral	PE-MP (virgin)	6, 60, 600 µg/day, 5 weeks	↑ *Staphylococcus*, ↓ *Parabacteroides*, ↑ TLR4/AP-1/IRF5	[264]
26.	Mice, oral	PS-MP (virgin)	5 µm and 400 nm, 100 mg/mL → 12.5 mg/mL (phase dilution), 4 h 5 min (5 min mouth + 2 h stomach + 2 h intestine)	↓ *Lactobacillus*, dysbiosis	[265]
27.	Mice, oral	PS-MP (virgin)	<25 µm, 30 g/kg feed → ~0.21 g MP/mouse/day (at ~7 g feed/day), 28 days	Dysbiosis (females > males), ↓ tight junctions	[266]
28.	Caco-2 cells (in vitro) + RNA-seq + qPCR	PS-MP	12.5/50.0 mg/L, 24 h	↓ viability (dose-dependent), 442 DEGs (210 ↑, 232 ↓); GO: CC, BP, MF; pathways: NF-κB, MAPK, cytokines, TLR → inflammation/proliferation; qPCR: ↑ Ras, ERK, MER, CDK4, Cyclin D1; ↑ TRPV1, iNOS, IL-1β, IL-8; intestinal inflammation mechanism	[268]
29.	Caco-2 cells (in vitro)	Polystyrene MNPL (nPS/y-nPS with fluorescent label)	—	Cellular penetration, ↑ cytotoxicity, ROS, genotoxicity, oxidative DNA damage, ↑ stress gene expression; nanotoxicity assessment via ingestion	[269]
30.	HepG2 and Caco-2 cells (incl. differentiated); LDH, MTT, HCS, ECIS	NP: PC and PET1 (laser ablation); PET2 (nanoprecipitation)	1–80 µg/mL, 24–48 h	Laser NP: ↓ size, ↑ distribution, ↑ oxidation; ↑ toxicity laser > PET2; PC > PET; HepG2/Caco-2: ↓ viability, ↑ ROS, ↓ mitochondrial activity, ↑ membrane permeability; Diff. Caco-2: no toxicity (barrier); ECIS: reversible barrier loss	[270]
31.	Caco-2 and HT-29 cells (in vitro, 48 h); MTT, LDH, DCFH-DA, DHE, MitoSOX	Ultra-high-molecular-weight PE (5–60 µm) + ethanol extracts	0.25–1.0 mg/mL	↓ viability (dose-dependent), ↑ mitochondrial superoxide (MitoSOX); extracts: similar (particle effect, not eluates); GI oxidative stress risk	[271]
32.	Fish (diet)	MP (various)	1–20 particles/individual (*M. barbatus*); 2–15 particles/individual (*M. merluccius*) (frequency: ~50–60% with MP)	↑ cytokines, antioxidant enzymes	[272]
33.	Mice, oral	PS-MP (virgin)	50 nm, 1 µm, 10 µm, 80 mg/L	↓ lipid digestion (heteroaggregates with lipases)	[273]
34.	Mice, oral	MNPs (composite)	20 mg/mL × 0.2 mL/day → 4 mg/mouse/day, 7 days	↓ *Alistipes* → obesity risk	[274]
35.	Mice + HFD	PS-COOH (fluorescent, GFP) (virgin)	0.45–0.53 µm, 1000 µg/L (1 mg/L) → ~0.05–0.1 mg/mouse/day	↑ permeability, inflammation, metabolic disruption	[275]
36.	Humans (colorectal tumors)	MNPs (various)	1–1299 µm	↑ MP content in tumors vs. healthy tissue	[277]

## 4. Discussion

Scientific data consistently show that MNPs exert systemic, multilevel effects on the body, in which general molecular and cellular mechanisms, such as oxidative stress [6,13,14,15,25,44,99,104,122,132,146,167,171,192,241], inflammation [6,15,21,33,41,56,155,218,225,276], mitochondrial dysfunction [5,15,32,35,77,94,115,138,216,270], apoptosis [6,7,26,28,119,121,136,185,216,218] and pyroptosis [4,23,40,77,110,141,155], autophagy [24,40,81,95,96,97], ferroptosis [28,94,118,128,221,223,228], and impaired barrier functions [19,28,40,53,54,108,130,133,204,205], are intertwined in a single network, realizing numerous interconnected pathologies [77]. Particle size plays a significant role: reduction to the nanometer range enhances their cytotoxicity due to penetration into cellular compartments and interaction with organelles. Thus, MNPs can interact with mitochondria through Drp1/Mfn2/OPA1 [31,214] or with the endoplasmic reticulum via the PERK/eIF2α/ATF4/CHOP axis [141], which enhances energy deficiency and causes metabolic disruption. MNPs also act as carriers for additives [88,89,90,91] and foreign contaminants [92], and the combined action of these components can often synergistically enhance key mechanisms of cell death, such as ferroptosis due to Fe^2+^ accumulation [28,118,128,220] and reduced GPX4 activity [124,127,220], as well as provoke microbiota disruption [56,227,242,252,256,267], forming a vicious cycle of inflammation via the gut–brain [19], gut–testis [61,201], and gut–kidney axes [7,8,227].

In the cardiovascular system, MNPs initiate a chain of events starting with oxidative stress and local inflammation, which lead to endothelial dysfunction, lipid metabolism disorders, and activation of hemostasis with subsequent cardiomyocyte damage [14,33]. At the molecular level, activation of the NLRP3-inflammasome in macrophages is observed, with increased pro-inflammatory cytokines and the integration of this response with mitochondrial disruptions [22]—suppression of PGC-1α and interaction of the NF-κB/NLRP3/GSDMD and AMPK/PGC-1α axes—which enhances myocardial apoptosis and promotes foam cell formation [6]. Size-dependent effects of particles around 0.5–1 μm disrupt the p38 MAPK/Smad2/Src/ERK/PLCγ1 pathways, inducing autophagy and endothelial necrosis [81], and the surface chemistry of the particles determines their effect on hemostasis [37,38]: aminated surfaces activate platelets through receptor-dependent mechanisms [86], and carboxylated ones through the intrinsic pathway, with a dose-dependent correlation observed between particle count and degree of platelet activation [87]. In cardiomyocytes, simultaneous increases in collagen I/III and fibronectin [35], cytoskeletal anomalies, enhanced inflammation [15,34], mitochondrial damage with disruption of the tricarboxylic acid cycle [34,35] and a shift toward oxidative stress [15] are recorded, leading to hypertrophy and fibrosis [26] through methylation mechanisms and reduced Na^+^/K^+^-ATPase activity [36]. Bisphenol A additionally increases arrhythmogenicity through ion channel modulation, and heavy metals promote ferroptosis and lipid peroxidation [88,89,90,91].

Neurotoxicity of MNPs manifests as a consequence of BBB penetration [5,40] and via the gut–brain axis [19,101]: oxidative stress stimulates pathological protein aggregation and microglial activation, and dysbiosis enhances neuroinflammation [16,101]. In clinical and preclinical models of Parkinson’s disease, polymer nanoparticles (25–100 nm range) destroy the intestinal barrier and microbiota, initiate ROS and mitochondrial stress [5], promote α-synuclein aggregation through NAC-domain interactions [102] and hydrophobic contacts, suppress autophagy via the mTOR/TFEB/TSC1–TSC2 pathway [40], and induce pyroptosis [41]. Melatonin, in turn, exhibits protective properties through AMPK/ULK1 activation [17], while heavy metals enhance damage through the PRKN/PDK1axes [105]. In Alzheimer’s disease, MNPs accelerate Aβ fibrillation [41,111] and tau [27,112], disrupt lysosomal function, and enhance microglial pyroptosis (GSDMD) with impaired phagocytosis [4], and the effects can synergize with genotypic factors, such as APOE4 and CYP1A1 polymorphisms [109]. Prenatal exposure is linked to increased tau phosphorylation at ser396 and ser199 and neurofibrillary tangle formation [18]. In ALS, TDP-43hyperphosphorylation and cytoplasmic accumulation involving oxidized HSP70 are recorded [44]. In stroke pathology, MNPs are associated with capillary obstruction, ferroptosis through reduced ZO-1, Fe^2+^ accumulation, and lipid peroxides [118] and pyroptosis activation through ASC/NLRP3/GSDMD activation [23]. Effects on the developing nervous system include reduced BDNF [121], disrupted Wnt-signaling [123], enhanced apoptosis [29,30,119,120] and autophagy. General BBB disruptions are also observed [5,19,40,108] through altered ZO-1, occluding, and ROS-mediated apoptosis induced by the p53/Bax/Bcl-2 signaling pathway [27,262] and enteric neuron loss [150] amid microbiota changes [151]. All these factors can lead to psychiatric disorders—from depression to anxiety and schizophrenia-like states, linked to neuroinflammation and synaptic dysregulation [45,46,47,48,49,154,155,158].

The reproductive system is particularly sensitive to MNPs: they cause hormonal disruptions [187,188,201,202], oxidative stress [171,173,185,186,189,191,192], and apoptosis [171,174,185,186], reducing fertility and exerting transgenerational effects. In the female reproductive tract, MNPs penetrate the ovaries, suppress adaptive antioxidant mechanisms [190], and activate the ER–stress axis PERK/eIF2α/ATF4/CHOP [189], leading to granulosa cell apoptosis, follicular atresia, and the development of fibrosis through Wnt/β-catenin activation [171]. Chemical additives, in particular DEHP, interact with the CNR1/CRBN/YY1/CYP2E1 axes, enhancing DNA damage and cell cycle arrest [192]. In the uterus, reduced endometrial gland count, impaired blood supply, and immunoregulation with adhesion and stenosis formation are noted [188,191,193,194], and in the placenta, metabolic disruptions [179,180,195,196] and iron homeostasis imbalance via IL-6 [198] are recorded, with possible particle translocation to fetal organs [179]. In men, MNP accumulation in the testes is accompanied by spermatogenesis disruption through pyruvate and thyroid metabolism imbalance [202], destabilization of the blood-testis barrier through ROS/MAPK/Nrf2/p38/NF-κB activation [205], and suppression of the hypothalamic-pituitary-gonadal (HPG) axis through reduced testosterone and LHR/cAMP/PKA/StAR [52], clinically manifesting as oligozoospermia and asthenozoospermia, confirmed by the detection of PS, PE, and PVC in semen [207,208,209].

In the kidneys and bladder, MNPs accumulate in the epithelium and interstitium, causing oxidative stress and reduced antioxidant defense, NF-κB-mediated inflammation, necroptosis activation via the RIP1/RIP3/MLKL pathway [31] and apoptosis [7], as well as autophagy induction through the AMPK/ULK1 signaling pathway [25] and disruption of key regulators of mitochondrial dynamics MFN1/2, OPA1, and Drp1 with a drop in mitochondrial membrane potential [31,214,215] in HK-2/HEK293 cells [216,217] and ER–stress activation via the IRE1/PERK/ATF6 pathway [55]. Experimental models demonstrate tubular and glomerular atrophy, developing fibrosis through TGF-β1/Klotho/Wnt/β-catenin signals [230], while the use of antioxidants NAC and MitoTEMPO shows a pronounced protective effect [216], confirming the therapeutic potential of interventions targeting oxidative stress.

Via the oral route, MNPs accumulate in the GIT epithelium and protective mucus [53,240,241,242], contribute to dysbiosis, growth of pathogenic strains, and disruption of barrier integrity through reduced expression of ZO-1, OCLN, CLDN-1, and MLCK activation [53], as well as via NF-κB/NLRP3-dependent mechanisms [53,54]. In the liver, this manifests as metabolic disruption, NAFLD development, insulin resistance [60,61], and chronic NF-κB-mediated inflammation [251], and in the pancreas—local inflammation [257] and increased risk of metabolic disorders and diabetes upon PET-MPs exposure with characteristic transcriptomic shifts [258]. Clinical associations cover inflammatory bowel disease, metabolic disorders, pancreatitis, and increased oncological risk.

Thus, the mechanisms of MNP action have a reproducible architecture across different organs but with organ-specific accents: impaired barrier functions and neuroinflammation in the CNS, hormonal dysregulation and transgenerational effects in reproduction, fibrosis and mitochondrial dysfunction in the heart and kidneys, and microbiota and metabolic disruption in the digestive tract and liver. MNPs function as an independent toxic agent and as a carrier for additives and contaminants, which enhances their pathogenicity and requires an interdisciplinary approach to prevention and treatment. Nevertheless, despite substantial progress in understanding the impacts of microplastics and nanoplastics on organ and system functioning, several critical gaps remain, including the need for timely detection and objective assessment of their toxicity and contribution to disease development, as well as the investigation of potential hidden and long-term effects, including possible influences on genetic mechanisms. However, it is already becoming evident that addressing these challenges will remain a major focus of scientific inquiry for decades to come.

## Figures and Tables

**Table 1:** Search Strategy for Each Database.

Database	Search Strategy
PubMed	(microplastic OR nanoplastic OR polymer particles OR polyethylene OR polypropylene OR polystyrene OR polyvinyl chloride OR plastic additives) AND (cardiovascular diseases OR endothelial dysfunction OR lipid metabolism OR hemostasis OR cardiomyocytes OR neurodegenerative diseases OR Parkinson’s disease OR Alzheimer’s disease OR amyotrophic lateral sclerosis OR stroke OR neuroinflammation OR psychiatric disorders OR depression OR anxiety disorders OR reproductive system OR fertility OR ovaries OR spermatogenesis OR urinary system OR kidneys OR gastrointestinal tract OR dysbiosis OR oxidative stress OR inflammation OR apoptosis OR autophagy OR ferroptosis OR pyroptosis OR mitochondrial dysfunction)
Scopus	TITLE-ABS-KEY (“microplastic” OR “nanoplastic” OR “polymer particles”) AND TITLE-ABS-KEY (“cardiovascular system” OR “nervous system” OR “reproductive system” OR “urinary system” OR “gastrointestinal tract” OR “neurodegenerative diseases” OR “psychiatric disorders” OR “oxidative stress” OR “inflammation”)
Web of Science	TOPIC (*microplastic* OR *nanoplastic* OR *polymer particles*) AND TOPIC (*cardiovascular diseases* OR *neurodegeneration* OR *psychiatric disorders* OR *reproductive toxicity* OR *kidneys* OR *gastrointestinal tract* OR *oxidative stress* OR *inflammation* OR *apoptosis* OR *autophagy* OR *ferroptosis* OR *pyroptosis*)

Note: *—wildcard search for word root; “”—exact keyword; ()—keyword grouping; “AND”—both terms required; “OR”—either term required.

**Table 3:** Experimental studies on neurodegenerative toxicity of microplastics and nanoplastics: models, particle types, concentrations, and key effects (n = 26 primary sources). Arrows ↑ and ↓ denote increase and decrease, respectively. Abbreviations: MPs—microplastics; NPs—nanoplastics; PS—polystyrene; PE—polyethylene; PVC—polyvinyl chloride; PP—polypropylene; APP/PS1—amyloid precursor protein/presenilin 1 transgenic mice; A53T—α-synuclein A53T mutant mice; APOE4—apolipoprotein E4 mice; BV2—murine microglial cell line; RAW264.7—murine macrophage cell line; SH-SY5Y—human neuroblastoma cell line; hCMEC/D3—human cerebral microvascular endothelial cell line; HT22—murine hippocampal neuronal cell line; MN9D—murine dopaminergic neuronal cell line; HeLa—human cervical cancer cell line; iPSC—induced pluripotent stem cell; *C. elegans*—*Caenorhabditis elegans*; Danio rerio—zebrafish; Aβ—amyloid-beta; α-syn—α-synuclein; TDP-43—TAR DNA-binding protein 43; GSDMD—gasdermin D; IL—interleukin; ROS—reactive oxygen species; AMPK—AMP-activated protein kinase; ULK1—Unc-51-like autophagy activating kinase 1; TLR2—Toll-like receptor 2; MMP9—matrix metalloproteinase 9; iNOS/nNOS—inducible/nitric oxide synthase; SIRT1—sirtuin 1; Nrf2—nuclear factor erythroid 2-related factor 2; Keap1—Kelch-like ECH-associated protein 1; HO-1—heme oxygenase-1; NQO1—NAD(P)H quinone dehydrogenase 1; mTOR—mechanistic target of rapamycin; TFEB—transcription factor EB; TSC1-TSC2—tuberous sclerosis complex 1/2; NAC—non-amyloid component; DRD1/DRD2—dopamine receptor D1/D2; th—tyrosine hydroxylase; slc6a3—solute carrier family 6 member 3 (dopamine transporter); SOD—superoxide dismutase; MDA—malondialdehyde; 8-OHdG—8-hydroxy-2′-deoxyguanosine; CK1—casein kinase 1; GSK3β—glycogen synthase kinase 3 beta; CYP1A1—cytochrome P450 family 1 subfamily A member 1; ZO-1—zonula occludens-1; MMSE—Mini-Mental State Examination; CSF—cerebrospinal fluid; BBB—blood–brain barrier; BBP—benzyl butyl phthalate; PRKN—parkin; PDK1—pyruvate dehydrogenase kinase 1; FITC—fluorescein isothiocyanate; BODIPY—boron-dipyrromethene; p-tau—hyperphosphorylated tau; ser—serine.

№	Experimental Model	Type and Source of MPs/NPs	Concentrations	Results/Observations	Reference
1.	APP/PS1 mice (in vivo, oral) + BV2, RAW264.7 (in vitro)	PS-NP, virgin	–	Microglial pyroptosis, ↑ IL-1β/IL-18, neuroinflammation, cognitive impairments; GSDMD inhibition suppresses pyroptosis	[4]
2.	Mice (in vivo, oral) + *C. elegans* (in vivo)	PS-MP/NP, virgin	1, 5 µm, 0.05, 0.1, 0.5 mg/day, 7–8 months	Intestinal barrier degradation, dysbiosis, ↑ ROS, mitochondrial stress, dopaminergic neuron degeneration, locomotor deficits	[5]
3.	A53T mice (in vivo, oral)	PS-NP, virgin	–	Mutant α-syn aggregation, ↑ cytokines, oxidative stress, membrane degradation	[16]
4.	Mice (in vivo, oral) + SH-SY5Y (in vitro)	PS-NP, virgin	250 mg/kg/day (mice, 28 days); 0.5–500 µg/mL (cells)	Complex I impairment, ↓ ATP, excessive mitophagy (AMPK/ULK1); melatonin restores mitophagy and locomotion	[17]
5.	Mice (in vivo, oral) + hCMEC/D3 and HT22	Amino-modified PS-NP	100 nm	↓ ZO-1/occludin (TLR2/MMP9), ↑ iNOS/nNOS, ↓ SIRT1, tau acetylation, apoptosis (p53/Bax); camellia pollen mitigates	[27]
6.	Chickens (in vivo, oral)	PS-MP, virgin	1 mg/L (L), 10 mg/L (M), 100 mg/L (H), 42 days	↓ BBB tight junction proteins, ferroptosis/apoptosis (↓ Nrf2-Keap1/HO-1/NQO1), pathological protein accumulation	[28]
7.	Mice (in vivo, oral); in vitro MN9D	PS-NP, virgin	10 mg/kg (1.5 h, in vivo); 7.5, 15, 30 mg/L (in vitro)	Autophagy inhibition (↑ mTOR, ↓ TFEB, TSC1-TSC2 degradation), pyroptosis activation, dopaminergic neuron death, cognitive deficits	[40]
8.	Microglia + in vitro (Aβ)	PS-NP, virgin	–	Protein corona formation, ↓ Aβ uptake, ABC transporter depletion, ↑ inflammation	[41]
9.	Wild-type + APP/PS1 mice (in vivo, oral)	PS-NP, virgin	PS or FITC-PS, lateral ventricle injection 5 µg/µL, 3 µL (5, 9, 17 weeks)	Microglia activation, hippocampal neuron death, liver steatosis, dysbiosis	[42]
10.	In vitro (α-syn)	PE-MP, PVC-MP, PS-MP, virgin	20–100 µg/L, 100 nm	Secondary structure changes, amyloidogenic oligomer formation	[43]
11.	In vitro (TDP-43) + in silico	PS-NP, virgin	HeLa cells: 25, 50, 100 µg/mL, 6, 12, 24 h	↑ Oxidative stress, cytoplasmic TDP-43 aggregation, HSP70 chaperoning loss, NP condensation	[44]
12.	*C. elegans* (in vivo) + human cells	PS-NP, virgin	10–1000 µg/mL	“Leaky gut”, mitochondrial fragmentation, α-syn aggregation	[101]
13.	In vitro (α-syn, SH-SY5Y), in vivo (mice)	PS-NP (virgin, aminated, carboxylated, mechanically abraded; 20–100 nm) pHrodo/FITC/BODIPY-labeled	In vitro: 0.1–10^11^ particles/mL (~0.01–1 µg/mL) In vivo: 10^10^–10^12^ particles/mouse (~0.1–10 µg), In vitro: 1–48 h In vivo: 3 days/2 months	Accelerated α-syn fibrillization (NAC domain), clathrin endocytosis, slowed lysosomal degradation	[102]
14.	In vitro (NACore surrogate) + microglia + *Danio rerio*	PS-NP, virgin	20 µg/mL and 100 µg/mL	NACore oligomerization modulation, ↑ microglial toxicity, hydrophobic interactions	[103]
15.	*Danio rerio* (in vivo, aqueous)	PS-NP, virgin	1.0, 5.0, 10.0 mg/L (6 days)	Neuroexcitation, ↑ ROS, ↓ atoh1a, altered neuroproteins	[104]
16.	In silico + in vitro (PRKN/PDK1)	PS-MP + BBP (impurity)	–	BBP interaction with PRKN/PDK1, enhanced neurodegeneration	[105]
17.	*Danio rerio* (in vivo, chronic)	PS-MP + Cu	2 mg/L ~1.09 × 10^8^ particles/L MP + 25 µg/L Cu (30 days)	↑ th, slc6a3; ↓ locomotion, dopaminergic dysregulation	[106]
18.	Primary cortical neurons (in vitro)	PS-NP, virgin	1.90 × 10^5^–3.03 × 10^11^ particles/mL	Dose-dependent ↑ DRD1/DRD2, dopaminergic system dysregulation	[107]
19.	Patients (CSF, n = 32)	PP, PVC, PE, PS (in CSF)	0.1–10 particles/mL (correlation with bottled water)	↑ PE/PVC in Aβ^+^; inverse correlation with Aβ42/MMSE; PE mediates cognitive decline	[108]
20.	APOE4 mice (in vivo, oral)	PS-MP/NP, mixed	0.125 mg/mL for 3 weeks	Sex-dependent locomotion/memory changes, astrocyte/microglia modification, ↑ CYP1A1 in liver	[109]
21.	APP/PS1 mice + BV2 (in vivo/in vitro)	PS-NP, virgin	10 or 20 mg/kg (mice, up to 2 months); 0, 25, 50, 100 µg/mL (cells)	↑ Aβ plaques, microglial pyroptosis, ↓ phagocytosis; melatonin restores microglial function	[110]
22.	In vitro (Aβ40/Aβ42)	PS-NP, virgin	25 g/L	Accelerated Aβ aggregation into toxic oligomers, hydrophobic interactions	[111]
23.	Mice (in vivo, prenatal)	PS-NP, virgin	7.57 × 10^11^, 3.76 × 10^12^, 1.88 × 10^13^ particles/day	↓ Offspring brain weight, neuron loss, Nissl degeneration, ↑ p-tau (ser396/199), ↑ Aβ	[112]
24.	Rats (in vivo, oral)	PE-MP, virgin	0.0375, 0.075, 0.15, 0.3, 0.6 mg	↓ SOD, Aβ42; ↑ MDA, 8-OHdG in hippocampus, oxidative stress, amyloid aggregates	[113]
25.	Dementia patients (postmortem brain)	Various polymers (in brain)	Higher in dementia vs. control (in vessels/immune cells)	MPs/NPs brain accumulation, dementia association	[114]
26.	Human iPSC-motoneurons (in vitro)	PS-NP, virgin	100 µg/mL and 1000 µg/mL (50 nm, 500 nm, 5000 nm)	Size-dependent penetration, TDP-43/CK1/GSK3β interaction, TDP-43 hyperphosphorylation, ↓ respiration, accelerated death	[115]

**Table 4:** Experimental studies on the role of MNPs in ischemic stroke: models, particle types, concentrations, and key effects (n = 5 primary sources). Arrows ↑ and ↓ denote increase and decrease, respectively. Abbreviations: MNPs—micro- and nanoplastics; MP—microplastics; NP—nanoplastics; PE-MP—polyethylene microplastic; PVC-MP—polyvinyl chloride microplastic; PS-MP—polystyrene microplastic; PS-NP—polystyrene nanoplastic; virgin—pristine/untreated plastic particles (no additives or aging); tMCAO—transient middle cerebral artery occlusion; ZO-1—zonula occludens-1 (tight junction protein); TEER—transepithelial/transendothelial electrical resistance; GSH—glutathione; Fe^2+^—ferrous iron; Fer-1—ferrostatin-1 (ferroptosis inhibitor); BBB—blood–brain barrier; ASC/NLRP3/GSDMD—pyroptosis pathway components (apoptosis-associated speck-like protein containing a CARD/NOD-like receptor family pyrin domain containing 3/gasdermin D); AMPK—AMP-activated protein kinase; p-tau—hyperphosphorylated tau protein; bEnd.3—brain endothelial cell line (mouse).

№	Experimental Model	Type and Source of MP/NP	Concentrations	Results/Observations	Reference
1.	C57BL/6J mice (in vivo)	PE-MP, PVC-MP, virgin	2 mg/mL (100 µL, ~6–7 mg/kg single dose)	Aggravation of neurological impairments: ↓ Garcia scores, ↑ deficit, ↓ motor function (rotarod test),↑ infarct volume (on day 3); PS-MP had no effect	[3]
2.	Chickens (in vivo, oral administration)	PS-MP, virgin	1–100 mg/kg feed (6 weeks)	Intracerebral hemorrhage, microthrombi, loss of Purkinje cells, inflammation infiltration, pyroptosis (ASC/NLRP3/GSDMD pathway), mitochondrial dysfunction (AMPK pathway)	[23]
3.	C57BL/6J mice (in vivo, tMCAO)	PS, PE and PVC, virgin	PS, 150 mg/kg/day; PE, 150 mg/kg/day; and PVC, 150 mg/kg/day, for 35 days	Enhanced neurological impairments: ↓ Garcia scores, ↑ deficit, ↓ motor function, ↑ infarct volume; PS-MP had no effect	[116]
4.	Mice (in vivo, global ischemia)	Mixed MP, virgin	50 mg/kg	↑ Neuroinflammation, microglial activation, myelin/microtubule damage, ↑ cytokines, ↑ p-tau, neuronal death	[117]
5.	Mice (in vivo, oral administration) + bEnd.3 cells (in vitro)	PS-NP, virgin	25 mg/kg/day (mice, 28 days); 12.5, 25, and 50 µg/mL (in vitro)	↓ ZO-1,↓ TEER, ferroptosis (↑ Fe^2+^, lipid peroxides, ↓ GSH); Fer-1 restores ZO-1 and BBB integrity	[118]

**Table 6:** Experimental studies on the effects of MNPs on the enteric nervous system and the gut–brain axis: models, particle types, concentrations, and key effects (n = 5 primary sources). Arrows ↑ and ↓ denote increase and decrease, respectively. Abbreviations: MNPs—micro- and nanoplastics; MP—microplastics; NP—nanoplastics; PLA-MP—polylactic acid microplastic; APLA-MP—aged polylactic acid microplastic; PET-MP—polyethylene terephthalate microplastic; PE-MP—polyethylene microplastic; PS-MP—polystyrene microplastic; PS-NP—polystyrene nanoplastic; virgin—pristine/untreated plastic particles (no additives or aging); CART—cocaine- and amphetamine-regulated transcript; GAL—galanin; nNOS—neuronal nitric oxide synthase; VAChT—vesicular acetylcholine transporter; VIP—vasoactive intestinal peptide; SP—substance P; IL-1β—interleukin 1 beta; IL-6—interleukin 6; IL-8—interleukin 8; IL-10—interleukin 10; TNF-α—tumor necrosis factor alpha; ABC—ATP-binding cassette; PI3K/AKT—phosphoinositide 3-kinase/protein kinase B.

№	Experimental Model	Type and Source of MP/NP	Concentrations	Results/Observations	Reference
1.	Zebrafish (in vivo, water exposure)	PLA-MP, APLA-MP (polylactic acid, aged), biodegradable	0.1–1 mg/L; acute: 96 h (larvae); chronic: 30 days (adults)	Thinning of intestinal wall, shortened villi, dysbiosis, ↓ neurotransmitters; neurotoxicity via gut–brain axis; bile acid mitigates effects	[54]
2.	Sows (in vivo, oral administration, ileum)	PET-MP (polyethylene terephthalate), virgin	PET-MP: 7.6–416.9 µm, 0.1–1 g/day, 28 days	↓ CART/GAL/nNOS/VAChT/VIP-positive neurons, ↑ GAL/SP-positive (submucosal/myenteric plexuses); thinning of mucosa/muscular layers; no changes in IL-1β/IL-6/IL-8/IL-10/TNF-α	[150]
3.	Mice (in vivo, oral administration)	PS-MP/NP, virgin	100 nm/1.0 µm, 0.5 mg/day, oral, 60 days	Anxiety-like behavior (open field, elevated plus maze); dysbiosis (↓ beneficial, ↑ pathogenic bacteria), ↓ mucus, ↑ intestinal permeability; metabolomic changes (ABC transporters, aminoacyl-tRNA, amino acids, bile); neurotransmitter dysregulation	[151]
4.	Adolescent mice (in vivo, oral administration)	PS-NP, virgin	5 µm/0.5 µm, 0.5 mg/day, oral, 4 weeks	Cognitive impairments, microbiota changes, hippocampal metabolome alterations, PI3K/AKT dysregulation	[152]
5.	Mice (in vivo, via food chain)	PE-MP, virgin	Accumulation through trophic chain (tadpoles → fish → mice), 7 days (mice)	Accumulation in gastrointestinal tract, anxiety-like behavior, ↓ locomotion	[153]

## Data Availability

No new data were created or analyzed in this study. Data sharing is not applicable to this article.

## Supplementary Materials

The following supporting information can be downloaded at: https://www.mdpi.com/article/10.3390/ijms262211194/s1.
