# Supplementary material for: Molecular and Cellular Effects of Microplastics and Nanoplastics in the Pathogenesis of Cardiovascular, Nervous, Urinary, Digestive, and Reproductive System Diseases: A Global Systematic Review
Source: Int J Mol Sci. 2025 Nov 19;26(22):11194. doi: 10.3390/ijms262211194 (PMC12653346; doi:10.3390/ijms262211194)
Supplement: Supplementary file 1 [file ijms-26-11194-s001.zip › ijms-3982701-supplementary.pdf]

Table S1. Expanded Search Strategy for Each Database. Note: * — wildcard search for word root; "" — exact keyword; () — keyword grouping; "AND" — both terms required; "OR" — either term required.

Database	Search Strategy
PubMed	((microplastic OR nanoplastic OR "polymer particles" OR polyethylene OR polypropylene OR polystyrene OR "polyvinyl chloride" OR "plastic additives" OR bisphenol OR "bisphenol A" OR "bisphenol S" OR phthalates OR "diethylhexyl phthalate" OR "polyethylene terephthalate" OR polyurethane OR "polylactic acid" OR "microplastic pollution" OR "nanoplastic toxicity" OR "plastic degradation" OR "polymer contaminants" OR "plastic nanoparticles") AND (cardiovascular diseases OR "endothelial dysfunction" OR "lipid metabolism" OR hemostasis OR cardiomyocytes OR "cardiac fibrosis" OR "atherosclerosis" OR "myocardial injury" OR "vascular inflammation" OR "hypertension" OR "neurodegenerative diseases" OR "Parkinson's disease" OR "Alzheimer's disease" OR "amyotrophic lateral sclerosis" OR stroke OR neuroinflammation OR "psychiatric disorders" OR depression OR "anxiety disorders" OR "schizophrenia" OR "bipolar disorder" OR "neurotoxicity" OR "cognitive impairment" OR "reproductive system" OR fertility OR ovaries OR spermatogenesis OR "hormonal imbalance" OR "reproductive toxicity" OR "testicular dysfunction" OR "ovarian dysfunction" OR "urinary system" OR kidneys OR "renal fibrosis" OR "chronic kidney disease" OR "nephrotoxicity" OR "glomerular dysfunction" OR "gastrointestinal tract" OR dysbiosis OR "gut microbiota" OR "inflammatory bowel disease" OR "metabolic disorders" OR "gut barrier" OR "oxidative stress" OR inflammation OR apoptosis OR autophagy OR ferroptosis OR pyroptosis OR "mitochondrial dysfunction" OR "barrier function" OR "cellular toxicity" OR "endoplasmic reticulum stress" OR "DNA damage"))
Scopus	TITLE-ABS-KEY ("microplastic" OR "nanoplastic" OR "polymer particles" OR "polyethylene" OR "polypropylene" OR "polystyrene" OR "polyvinyl chloride" OR "plastic additives" OR "bisphenol A" OR "bisphenol S" OR "phthalates" OR "diethylhexyl phthalate" OR "polyethylene terephthalate" OR "polyurethane" OR "polylactic acid" OR "microplastic toxicity" OR "nanoplastic exposure" OR "plastic degradation products" OR "polymer contaminants" OR "plastic nanoparticles" OR "microparticle pollution" OR "nanoparticle toxicity") AND TITLE-ABS-KEY ("cardiovascular system" OR "cardiac dysfunction" OR "endothelial cells" OR "lipid metabolism disorders" OR "hemostatic disorders" OR "cardiomyocyte toxicity" OR "atherosclerosis" OR "myocardial fibrosis" OR "vascular inflammation" OR "hypertension" OR "coronary artery disease" OR "nervous system" OR "neurodegenerative disorders" OR "neuroinflammation" OR "psychiatric disorders" OR "depressive disorders" OR "anxiety disorders" OR "schizophrenia" OR "bipolar disorder" OR "neurotoxicity" OR "cognitive dysfunction" OR "brain inflammation" OR "reproductive system" OR "fertility impairment" OR "ovarian dysfunction" OR "spermatogenesis impairment" OR "hormonal disruption" OR "endocrine toxicity" OR "testicular toxicity" OR "urinary system" OR "renal dysfunction" OR "kidney fibrosis" OR "nephrotoxicity" OR "glomerular damage" OR "chronic kidney disease" OR "gastrointestinal tract" OR "gut microbiota" OR "dysbiosis" OR "inflammatory bowel disease" OR "metabolic syndrome" OR "gut barrier dysfunction" OR "oxidative stress" OR "chronic inflammation" OR "apoptosis" OR "autophagy" OR "ferroptosis" OR "pyroptosis" OR "mitochondrial dysfunction" OR "barrier dysfunction" OR "cellular stress" OR "DNA damage" OR "protein misfolding")

Web of Science	TOPIC: (*microplastic* OR *nanoplastic* OR *polymer particles* OR *polyethylene* OR *polypropylene* OR *polystyrene* OR *polyvinyl chloride* OR *plastic additives* OR *bisphenol A* OR *bisphenol S* OR *phthalates* OR *diethylhexyl phthalate* OR *polyethylene terephthalate* OR *polyurethane* OR *polylactic acid* OR *microplastic pollution* OR *nanoplastic toxicity* OR *plastic degradation* OR *polymer contaminants* OR *plastic nanoparticles* OR *microparticle exposure* OR *nanoparticle pollution*) AND TOPIC: (*cardiovascular diseases* OR *endothelial dysfunction* OR *lipid metabolism disorders* OR *hemostatic dysfunction* OR *cardiomyocyte damage* OR *atherosclerosis* OR *myocardial fibrosis* OR *vascular inflammation* OR *hypertension* OR *coronary artery disease* OR *neurodegeneration* OR *neuroinflammatory response* OR *psychiatric disorders* OR *depressive disorders* OR *anxiety disorders* OR *schizophrenia* OR *bipolar disorder* OR *neurotoxicity* OR *cognitive impairment* OR *brain inflammation* OR *reproductive toxicity* OR *fertility impairment* OR *ovarian dysfunction* OR *spermatogenesis disruption* OR *hormonal imbalance* OR *endocrine disruption* OR *testicular damage* OR *urinary system* OR *renal fibrosis* OR *kidney dysfunction* OR *nephrotoxicity* OR *glomerular dysfunction* OR *chronic kidney disease* OR *gastrointestinal tract* OR *gut dysbiosis* OR *metabolic disorders* OR *inflammatory bowel disease* OR *gut barrier impairment* OR *oxidative stress* OR *inflammation* OR *apoptosis* OR *autophagy* OR *ferroptosis* OR *pyroptosis* OR *mitochondrial dysfunction* OR *barrier function impairment* OR *cellular toxicity* OR *endoplasmic reticulum stress* OR *DNA damage*)

Table S2. Characteristics and distribution of included studies by thematic categories, n=277.

Section	Category	Number	List
Introduction	Articles	20	[1–2], [9–12], [62–75]
Materials and Methods	Articles	1	[76]
Cardiovascular diseases	Experimental	21	[13–14], [22], [26], [34–35], [39], [78–79], [81–83], [85], [87], [89–91], [97–100]
	Reviews	17	[6], [15], [24], [33], [36–38], [77], [80], [84], [86], [88], [92–96]
Neurodegenerative diseases	Experimental	26	[4–5], [16–17], [27–28], [40–44], [101–115]
	Reviews	2	[18–19]
Stroke	Experimental	5	[3], [23], [116–118]
Neurological disorders	Experimental	34	[20–21], [29–30], [32], [119–132], [133–136], [138–142], [144–149]
	Reviews	2	[137], [143]
Enteric nervous system and gut–brain axis	Experimental	5	[54], [150–153]
Mental disorders	Experimental	20	[45–50], [154–155], [157–168]
	Reviews	1	[156]
Reproductive system	Experimental	32	[51–52], [171–174], [178–180], [182–183], [185–186], [188–190], [191–202], [204], [207–209]
	Reviews	11	[169–177], [181], [184], [187], [203], [205–206]

Urinary system	Experimental	27	[25], [31], [210], [213], [215], [217–238]
	Reviews	8	[7–8], [55], [211–212], [214], [216], [239]
Gastrointestinal tract	Experimental	36	[53], [56–61], [243], [247–251], [253–266], [268–275], [277]
	Reviews	9	[240–246], [252], [267], [276]

Table S3. Summary of experimental data on cardiovascular toxicity of microplastics and nanoplastics: key models and effects (n=21 sources). Abbreviations: MP – microplastics; NP – nanoplastics; PS – polystyrene; S-NP – spherical NP; F-NP – fragmented NP; NH_2 – amino-modified; PVC – polyvinyl chloride; PE – polyethylene; PET – polyethylene terephthalate; PMMA – polymethyl methacrylate; PAN – polyacrylonitrile; PA6 – polyamide 6; BPA – bisphenol A; PC – polycarbonate; virgin – pristine/untreated plastic particles (no additives or aging); aPS – anionic PS; cPS – cationic PS; nPS – neutral PS; COOH – carboxylated; fPS-MP – fluorescent PS-MP; ↑ – increase/increased; ↓ – decrease/decreased; NLRP3 – NOD-like receptor family pyrin domain containing 3; IL-1β – interleukin 1 beta; IL-18 – interleukin 18; TNF-α – tumor necrosis factor alpha; IL-6 – interleukin 6; BP – blood pressure; LV – left ventricle; TCA – tricarboxylic acid (cycle); HUVEC – human umbilical vein endothelial cells; PCAEC – porcine coronary artery endothelial cells; HR – hazard ratio; MI – myocardial infarction; DCM – dilated cardiomyopathy; ERβ – estrogen receptor beta; NF-κB – nuclear factor kappa B; GSDMD – gasdermin D; AMPK – AMP-activated protein kinase; PGC-1α – peroxisome proliferator-activated receptor gamma coactivator 1 alpha; m6A – N^6-methyladenosine; METTL3 – methyltransferase-like 3; Na^+-K^+-ATPase – sodium-potassium ATPase.

Model	Type of MP/NP	Dose / Duration	Key effects	Sources
**Macrophages, monocytes, dendritic cells (in vitro, mouse/human)**	PS (S-NP, F-NP, NH_2, 50–310 nm), PVC, PE, PET, PMMA, PAN, PA6	30–300 particles/cell; 50–100 µg/mL; 24–48 h	NLRP3 inflammasome activation ↑ IL-1β, IL-18, TNF-α, IL-6; foam cell differentiation; contribution to atherogenesis	[13,22,79]
**Animals (in vivo, oral/inhalation)**	PS-MP/NP (virgin, 0.2–5 µm), BPA	0.5–100 mg/kg/day; 1–12 weeks; 0.5–200 µg/kg/day (BPA)	Lipid metabolism disruption, oxidative stress, systemic inflammation, ↑ BP, LV remodeling, obesity, sexual dimorphism	[14,26,34,78,90]
**Cardiomyocytes, cardiac organoids (in vitro/in vivo)**	PS-MP/NP (virgin, 0.2–5 µm)	0.025–2.5 µg/mL; 4–72 h; 25–50 µg/injection	↑ TNF-α, IL-6, IL-1β; mitochondrial dysfunction, ↓ TCA cycle, apoptosis, fibrosis, impaired	[34,35,78]

			contractility, ↑ collagen I/III	
**Endothelium (HUVEC, PCAEC, in vitro)**	PS-NP (virgin, 100–500 nm; 0.5–5 µm)	10–100 µg/mL; 24–72 h	↓ Viability (small sizes only), angiogenesis suppression, ↑ autophagy/necrosis, premature senescence, inflammation	[81,97,99]
**Hemostasis system (in vitro: blood, erythrocytes, plasma; in vivo: rats, mice)**	PS (aPS, cPS, nPS, NH_2, COOH; 50–500 nm), PS-MP	0.01–250 µg/mL; 0.1–4 mg/kg; 3–24 h	Accelerated clotting, ↑ clot strength, thrombosis, platelet/erythrocyte activation, coagulopathy	[39,87,100]
**Clinical data: plaques, thrombi, feces, serum**	PE, PVC, PET, BPA	20–530 particles/plaque; ↑ in feces with calcification; ↑ BPA in DCM	HR 4.2 (MI, stroke, death); correlation with thrombogenesis and vascular calcification	[82,83,85,89]
**Bisphenol A (BPA) — model endocrine disruptor from plastics**	BPA (from PC, epoxy resins)	10^{-12}–10^{-6} M (acute); 0.5–5 µg/kg/day (chronic)	Arrhythmias, Ca^{2+} homeostasis disruption (via ERβ), ↑ body weight, LV remodeling, sexual dimorphism	[90,91]
**Multi-level models (in silico + in vitro + in vivo)**	fPS-MP (1 µm), PS	Dose-dependent; oral/i.v.	Microglial binding, blood/brain accumulation, ↑ insulin resistance, systemic inflammation in obesity	[98]
**Cardiotoxic signaling pathways**	PS-MP/NP, BPA	Various	↑ NF-κB–NLRP3–GSDMD, ↓ AMPK–PGC-1α, ↑ m6A/METTL3, ↓ Na^+-K^+-ATPase, epigenetic changes, mitochondrial damage	[26,34,78,90]

Table S4. Summary of experimental data on neurotoxicity of micro- and nanoplastics: key models and mechanisms (n=26 sources). Abbreviations: MP – microplastics; NP – nanoplastics; PS-NP – polystyrene nanoplastic; PS-MP – polystyrene microplastic; PE-MP – polyethylene microplastic; PVC-MP – polyvinyl chloride microplastic; PP – polypropylene; virgin – pristine/untreated plastic particles (no additives or aging); ↑ – increase/increased; ↓ – decrease/decreased; NLRP3 – NOD-like receptor family pyrin domain containing 3; GSDMD – gasdermin D; IL-1β – interleukin 1 beta; IL-18 – interleukin 18; Aβ – amyloid-beta; ROS – reactive oxygen species; DRD1/DRD2 – dopamine receptors 1/2; th – tyrosine hydroxylase; slc6a3 – dopamine transporter; HSP70 – heat shock protein 70; ZO-1 – zonula occludens-1; BBB – blood-brain barrier; Nrf2 – nuclear factor erythroid 2-related factor 2; HO-1 – heme oxygenase 1; ATP – adenosine triphosphate; SIRT1 – sirtuin 1; AMPK – AMP-activated protein kinase; ULK1 – Unc-51-like autophagy activating kinase 1; mTOR – mechanistic target of rapamycin; TFEB – transcription factor EB; TSC1-TSC2 – tuberous sclerosis complex 1/2; p-tau – hyperphosphorylated tau; SOD – superoxide dismutase; MDA – malondialdehyde; CSF – cerebrospinal fluid; MMSE – Mini-Mental State Examination; BBP – benzyl butyl phthalate; CYP1A1 – cytochrome P450 1A1; PRKN – parkin; PDK1 – pyruvate dehydrogenase kinase 1; Keap1 – Kelch-like ECH-associated protein 1; TLR2 – Toll-like receptor 2; MMP9 – matrix metalloproteinase 9; iNOS – inducible nitric oxide synthase; nNOS – neuronal nitric oxide synthase; m6A – N^6-methyladenosine; APP/PS1 – amyloid precursor protein/presenilin 1 transgenic model; A53T – α-synuclein A53T mutant model; SH-SY5Y – human neuroblastoma cell line; MN9D – mouse dopaminergic cell line; HT22 – mouse hippocampal cell line; hCMEC/D3 – human cerebral microvascular endothelial cell line (D3).

Model	Type of MP/NP	Dose / Duration	Key effects	Sources
**Microglia (in vitro/in vivo: BV2, RAW264.7, APP/PS1)**	PS-NP (virgin, 50–100 nm)	0–100 μg/mL; 10–20 mg/kg; 6 h – 2 months	Pyroptosis (↑ NLRP3/GSDMD, IL-1β/IL-18), ↓ Aβ phagocytosis, ↑ inflammation; melatonin/GSDMD inhibition mitigates	[4,41,42,110]
**Dopaminergic neurons, locomotion (in vivo: A53T mice, *C. elegans*; in vitro: SH-SY5Y, MN9D)**	PS-NP/MP (virgin, 20–5000 nm), PS+Cu	0.05–250 mg/kg/day; 0.5–500 μg/mL; 7 days – 8 months	Dopaminergic neuron degradation, ↓ locomotion, ↑ ROS, DRD1/DRD2 dysregulation, ↑ th/slc6a3	[5,16,17,40,106,107]
**Pathological protein aggregation (in vitro/in silico: α-synuclein, Aβ, TDP-43)**	PS-NP (virgin, aminated, carboxylated), PE/PVC-MP	0.01–1000 μg/mL; 10^5–10^{11} particles/mL; 1–48 h	Accelerated fibrillization/oligomerization, protein corona, hydrophobic interactions, HSP70 loss	[43,44,102,103,111]
**Blood-brain barrier and gut–brain axis (in vivo:**	PS-NP/MP (virgin, 100 nm – 5 μm)	1–100 mg/L; 10 mg/kg; 1.5	↓ ZO-1/occludin, leaky gut, dysbiosis, ↑ BBB permeability, ferroptosis (↓ Nrf2/HO-1)	[5,27,28,101]

**mice, chickens; in vitro: hCMEC/D3)**		h – 42 days		
**Mitochondrial dysfunction (in vitro/in vivo: SH-SY5Y, HT22, neurons)**	PS-NP (virgin, 50–500 nm)	0.5–500 µg/mL; 7.5–30 mg/L; 4–72 h	↓ Complex I, ↓ ATP, mitochondrial fragmentation, ↑ p53/Bax, ↓ SIRT1	[5,17,27,44,101,115]
**Autophagy and mitophagy (in vitro/in vivo: SH-SY5Y, MN9D, mice)**	PS-NP (virgin)	0.5–500 µg/mL; 250 mg/kg/day; 4–72 h; 28 days	Excessive/blocked autophagy (↑ AMPK/ULK1; ↑ mTOR, ↓ TFEB, ↓ TSC1-TSC2); melatonin restores	[17,40]
**Prenatal/chronic exposure (in vivo: mice, rats, *Danio rerio*)**	PS-NP/MP (virgin, mixed), PE-MP	0.0375–0.6 mg; 7.57×10^{11}–1.88×10^{13} particles/day; 6–30 days	↓ brain weight, neuron loss, ↑ p-tau, Aβ, oxidative stress (↓ SOD, ↑ MDA), hyperexcitability	[104,112,113]
**Clinical data (CSF, postmortem brain, n=32+)**	PP, PVC, PE, PS	0.1–10 particles/mL (CSF); ↑ in dementia	Accumulation in brain/CSF, correlation with $A\beta^{+}$, ↓ Aβ42/MMSE, dementia association	[108,114]
**Modifiers: genetics, impurities, additives (in vivo/in vitro: APOE4, BBP, camellia pollen)**	PS-NP/MP, BBP (impurity)	0.125 mg/mL; 3 weeks; docking	Sex-dependent cognitive deficits, ↑ CYP1A1, BBP → PRKN/PDK1, camellia pollen mitigation	[27,105,109]
**Common signaling pathways and cellular entry**	PS-NP/MP, PE, PVC	Various	↑ ROS, ↓ Nrf2-Keap1, ↑ TLR2/MMP9, ↑ iNOS/nNOS, clathrin-endocytosis, lysosomal dysfunction, ↑ m6A (epigenetics)	[5,17,27,40,44,102,105,109]

Table S5. Experimental studies on the role of MNPs in ischemic stroke: models, particle types, concentrations, and key effects (n=5 primary sources). Abbreviations: MNPs – micro- and nanoplastics; MP – microplastics; NP – nanoplastics; PS-MP – polystyrene microplastic; PS-NP – polystyrene nanoplastic; PE-MP – polyethylene microplastic; PVC-MP – polyvinyl chloride microplastic; virgin – pristine/untreated plastic particles (no additives or aging); ↑ – increase/increased; ↓ – decrease/decreased; tMCAO – transient middle cerebral artery occlusion; ASC – apoptosis-associated speck-like protein containing a CARD; NLRP3 – NOD-like receptor family pyrin domain containing 3; GSDMD – gasdermin D; AMPK – AMP-activated protein kinase; ZO-1 – zonula occludens-1; TEER – trans-endothelial electrical resistance; GSH – glutathione; Fer-1 – ferrostatin-1; BBB – blood-brain barrier.

Experimental model	Type and source of MP/NP	Concentrations	Results / observations	Reference
**C57BL/6J mice (in vivo)**	PE-MP, PVC-MP (polyethylene, polyvinyl chloride), virgin	2 mg/mL (100 µL, ~6–7 mg/kg single dose)	Aggravated neurological deficits: ↓ Garcia scores, ↑ deficit, ↓ motor function (rotarod), ↑ infarct volume (day 3); PS-MP no effect	[3]
**Chickens (in vivo, oral)**	PS-MP, virgin	1–100 mg/kg feed (6 weeks)	Intracerebral hemorrhage, microthrombi, Purkinje cell loss, inflammatory infiltration, pyroptosis (ASC/NLRP3/GSDMD), mitochondrial dysfunction (AMPK)	[23]
**C57BL/6J mice (in vivo, tMCAO)**	PS (polystyrene), PE (polyethylene), PVC (polyvinyl chloride), virgin	PS: 150 mg/kg/day; PE: 150 mg/kg/day; PVC: 150 mg/kg/day; 35 days	Enhanced neurological deficits: ↓ Garcia, ↑ deficit, ↓ motor function, ↑ infarct volume; PS-MP no effect	[116]
**Mice (in vivo, global ischemia)**	Mixed MP, virgin	50 mg/kg	↑ neuroinflammation, microglial activation, myelin/microtubule damage, ↑ cytokines, ↑ p-tau, neuronal death	[117]
**Mice (in vivo, oral) + bEnd.3 (in vitro)**	PS-NP, virgin	25 mg/kg/day (mice, 28 days); 12.5, 25, 50 µg/mL (cells)	↓ ZO-1, ↓ TEER, ferroptosis (↑ Fe^{2+}, lipid peroxides, ↓ GSH); Fer-1 restores ZO-1 and BBB	[118]

Table S6. Summary of experimental data on neurotoxicity and neurodevelopmental toxicity of micro- and nanoplastics: key models and mechanisms (n=34 sources). Abbreviations: MP – microplastics; NP – nanoplastics; PS-NP – polystyrene nanoplastic; PS-MP – polystyrene microplastic; PE-MP – polyethylene microplastic; PP – polypropylene; PLA-NP – polylactic acid nanoplastic; virgin – pristine/untreated plastic particles (no additives or aging); ↑ – increase/increased; ↓ – decrease/decreased; hNSC – human neural stem cells; Wnt – Wingless/Integrated; KYN – kynurenine; 3-HK – 3-hydroxykynurenine; hpf – hours post-fertilization; GPX4 – glutathione peroxidase 4; GSH – glutathione; ROS – reactive oxygen species; ZO-1 – zonula occludens-1; TEER – trans-endothelial electrical resistance; MDA – malondialdehyde; BDNF – brain-derived neurotrophic factor; TLR4 – Toll-like receptor 4; NF-κB – nuclear factor kappa B; NLRP3 – NOD-like receptor family pyrin domain containing 3; DEGs – differentially expressed genes; ECM – extracellular matrix; mtROS – mitochondrial ROS; PI3K/AKT – phosphoinositide 3-kinase/protein kinase B; GSK-3β – glycogen synthase kinase 3 beta; PERK – protein kinase R-like endoplasmic reticulum kinase; CHOP – C/EBP homologous protein; LPO – lipid peroxidation; 8-OHdG – 8-hydroxy-2'-deoxyguanosine; SOD – superoxide dismutase; CAT – catalase; MICU3 – mitochondrial calcium uptake 3; SIRT3 – sirtuin 3; DA – dopamine; 5-HT – serotonin; ACh – acetylcholine; AChE – acetylcholinesterase; NH_3 – ammonia; Glu – glutamate; GABA – gamma-aminobutyric acid; mTOR – mechanistic target of rapamycin; MeHg – methylmercury; Cd – cadmium; DEHP – di(2-ethylhexyl) phthalate; AMI – amitriptyline; FOXO3a – forkhead box O3a; HDAC6 – histone deacetylase 6; DOCK3 – dedicator of cytokinesis 3; GRP78 – glucose-regulated protein 78; bEnd.3 – mouse brain endothelial cell line.

Model / Condition	Type of MP/NP	Dose / Duration	Key effects	Sources
**Cerebral organoids, hNSC (in vitro)**	PS-NP (virgin, 50–200 nm)	0.1–100 μg/mL; 21–24 days	↓ viability/proliferation/differentiation, mitochondrial dysfunction, ↓ Wnt/N-cadherin, ↑ KYN/3-HK, apoptosis	[20,30,123,145]
**Prenatal/postnatal exposure (in vivo: mice, rats, chickens, *Danio rerio*)**	PS-NP/MP (virgin, 50 nm – 5 μm), PLA-NP	2.5 mg/kg – 25 mg/L; 1–144 hpf – 6 weeks	Neural tube defects, ↓ axon length, ↓ swimming, ferroptosis (NCOA4/p53), ↓ GPX4/GSH, ↑ ROS	[29,119,124,138]
**Blood-brain barrier (in vivo/in vitro: mice, bEnd.3)**	PS-NP/MP (virgin, 42 nm – 2 μm), PE-MP	0.5–200 μg/mL; 0.5–50 mg/kg; 7 days – 6 weeks	↓ ZO-1/TEER, ↑ permeability (15.6–27.3×), endothelial necroptosis, ↑ MDA, ↓ BDNF	[129,130,134]
**Neuroinflammation, microglia, astrogliosis**	PS-NP/MP (virgin), PE/PP/PS	0.01–1000 μg/mL; 30 mg/kg;	↑ TLR4-NF-κB-NLRP3, ↑ cytokines, astrogliosis, 1274/531 DEGs (inflammation/ECM), ↑ miR-103a-3p	[21,128,141,142]

**(in vivo/in vitro)**		7 days – 8 weeks		
**Apoptosis, necrosis, ferroptosis (in vivo/in vitro: hippocampus, organoids, fish)**	PS-NP/MP (virgin), PE-MP	0.5–150 µM; 0.5–5 mg/kg; 96 h – 8 weeks	↑ mtROS/Ca^{2+}, ↑ Fe^{2+}/lipid peroxides, ↓ GSH, ↑ PI3K/AKT/GSK-3β, ↑ PERK/CHOP	[20,32,119,128,136]
**Mitochondrial dysfunction, oxidative stress**	PS-NP/MP (virgin), PLA-NP	1–100 µg/mL; 0.1–25 mg/kg; 4–72 h – 28 days	↑ ROS/LPO/8-OHdG, ↓ SOD/CAT/GSH, ↑ Ca^{2+} (MICU3), fragmentation, ↓ SIRT3	[20,32,121,135,136,138]
**Neurotransmitters, behavior (*Danio rerio*, *C. elegans*, fish)**	PS-NP/MP (virgin, 25 nm – 5 µm)	0.1–1000 µg/mL; 10^4–10^6 particles/L; 96 h – 3 weeks	↓ DA/5-HT/ACh, ↑ AChE, ↓ swimming/locomotion, ↑ anxiety, sel-12/hop-1 dysregulation	[121,122,125,126,127,139]
**Cognitive impairment, memory (in vivo: mice, rats)**	PS-MP (virgin, 5 µm), PE-MP	0.01–50 mg/kg/day; 15–60 days	↓ memory/recognition, limbic dystrophy, ↑ NH_3/Glu, ↓ GABA/mTOR	[124,132,139,141]
**Combined toxicity with pollutants (MeHg, Cu, Cd, DEHP, amitriptyline, ketamine)**	PS-MP/NP + MeHg/Cu/Cd/DEHP/AMI	0.14–2 mg/L MP + 0.85 mg/L Cu – 25 µM DEHP; 96 h – 60 days	Additive/synergistic neurotoxicity, ↑ ROS/apoptosis, Cd–MP antagonism, ↑ BDNF/miR132/FOXO3a	[120,131,135,136,140,146,147]
**Protein corona, proteomics (in vitro: neurons/glia)**	PE/PP/PS-MP (virgin)	1–100 µg/mL; 24–72 h	Heterogeneous corona, altered proteins (synthesis/RNA/lipids/transport), ↑ inflammation	[148]
**Clinical/post mortem data**	16 polymers (PP 43.8%, particles/fibers)	5.5–26.4 µm	Translocation via olfactory pathway, brain	[133]

**(olfactory bulbs, n=15)**		(particles); 8/15 positive	accumulation, neurodegeneration risk	
**Protective agents (melatonin, curcumin, astaxanthin, lycopene, E4A, miR-103a-3p, chlorella)**	PS-NP/MP (virgin)	1 µM – 500 mg/kg; 7–56 days	BBB/cognitive restoration, ↓ ROS/inflammation/ferroptosis, ↑ nrf2/isl2a/SIRT1	[21,119,120,128,132,141,149]
**Intranasal/olfactory accumulation (in vivo: mice)**	PS-NP (virgin, 100 nm)	Intranasal	Brain accumulation, neurotoxicity; HDAC6 inhibition ↑ exocytosis	[144]
**Common signaling pathways**	PS-NP/MP, PE, PLA	Various	↑ TLR4-NF-κB-NLRP3, ↓ DOCK3/SIRT3/BDNF, ↑ PERK/CHOP/GRP78, ↑ p53/Bax, clathrin-endocytosis	[20,21,128,136,141,142]

Table S7. Experimental studies on the effects of MNPs on the enteric nervous system and the gut–brain axis: models, particle types, concentrations, and key effects (n=5 primary sources). Abbreviations: MNPs – micro- and nanoplastics; MP – microplastics; NP – nanoplastics; PLA-MP – polylactic acid microplastic; APLA-MP – aged polylactic acid microplastic; PET-MP – polyethylene terephthalate microplastic; PE-MP – polyethylene microplastic; PS-MP – polystyrene microplastic; PS-NP – polystyrene nanoplastic; virgin – pristine/untreated plastic particles (no additives or aging); ↓ – decrease/decreased; ↑ – increase/increased; CART – cocaine- and amphetamine-regulated transcript; GAL – galanin; nNOS – neuronal nitric oxide synthase; VAChT – vesicular acetylcholine transporter; VIP – vasoactive intestinal peptide; SP – substance P; IL-1β – interleukin 1 beta; IL-6 – interleukin 6; IL-8 – interleukin 8; IL-10 – interleukin 10; TNF-α – tumor necrosis factor alpha; ABC – ATP-binding cassette; PI3K/AKT – phosphoinositide 3-kinase/protein kinase B.

Experimental model	Type and source of MP/NP	Concentrations	Results / observations	Reference
**Zebrafish (*Danio rerio*) (in vivo, water exposure)**	PLA-MP, APLA-MP (polylactic acid, aged), biodegradable	0.1–1 mg/L; acute: 96 h (larvae); chronic: 30 days (adults)	Thinning of intestinal wall, shortened villi, dysbiosis, ↓ neurotransmitters; neurotoxicity via gut–brain axis; bile acid mitigates effects	[54]
**Sows (in vivo, oral, ileum)**	PET-MP (polyethylene	PET-MP: 7.6–416.9 µm, 0.1–	↓ CART/GAL/nNOS/VAChT/VIP-positive neurons, ↑ GAL/SP-	[150]

	terephthalate), virgin	1 g/day, 28 days	positive (submucosal/myenteric plexuses); thinning of mucosa/muscular layers; no changes in IL-1β/IL-6/IL-8/IL-10/TNF-α	
**Mice (in vivo, oral)**	PS-MP/NP, virgin	100 nm / 1.0 µm, 0.5 mg/day, oral, 60 days	Anxiety-like behavior (open field, elevated plus maze); dysbiosis (↓ beneficial, ↑ pathogenic bacteria), ↓ mucus, ↑ intestinal permeability; metabolomic changes (ABC transporters, aminoacyl-tRNA, amino acids, bile); neurotransmitter dysregulation	[151]
**Adolescent mice (in vivo, oral)**	PS-NP, virgin	5 µm / 0.5 µm, 0.5 mg/day, oral, 4 weeks	Cognitive impairments, microbiota changes, hippocampal metabolome alterations, PI3K/AKT dysregulation	[152]
**Mice (in vivo, via food chain)**	PE-MP, virgin	Accumulation through trophic chain (tadpoles → fish → mice), 7 days (mice)	Accumulation in gastrointestinal tract, anxiety-like behavior, ↓ locomotion	[153]

Table S8. Summary of experimental data on the role of micro- and nanoplastics in mental disorders: key models and mechanisms (n=20 sources). Abbreviations: MP – microplastics; NP – nanoplastics; PS-NP – polystyrene nanoplastic; PS-MP – polystyrene microplastic; PP-MP – polypropylene microplastic; PLA-MP – polylactic acid microplastic; virgin – pristine/untreated plastic particles (no additives or aging); ↑ – increase/increased; ↓ – decrease/decreased; EAAT2 – excitatory amino acid transporter 2; IL-6 – interleukin 6; IL-1β – interleukin 1 beta; p38 MAPK – p38 mitogen-activated protein kinase; NAC – N-acetylcysteine; ROS – reactive oxygen species; DA – dopamine; 5-HT – serotonin; GABA – gamma-aminobutyric acid; GD – gestational day; PND – postnatal day; ADHD – attention-deficit/hyperactivity disorder; PERK – protein kinase R-like endoplasmic reticulum kinase; NF-κB – nuclear factor kappa B; HRAS – Harvey rat sarcoma viral oncogene homolog; TNFα – tumor necrosis factor alpha; ACh – acetylcholine; CREB – cAMP response element-binding protein; BDNF – brain-derived neurotrophic factor; SIRT1 – sirtuin 1; NLRP3 – NOD-like receptor family pyrin domain containing 3.

Model / Condition	Type of MP/NP	Dose / Duration	Key effects	Sources
**Clinical data (children/students, n=1,420–5,670)**	Various MP (urine/air)	Quartiles/particles·mL^{-1}·day^{-1}	↑ depression risk (+38%), hyperactivity, inattention, ↓ working memory, emotional problems	[45,50,166]

**Depression-like behavior (in vivo: mice)**	PS-NP/MP (virgin, 60–100 nm)	0.1–50 mg/kg/day; 7 days – 8 weeks	↓ EAAT2, ↑ IL-6/IL-1β, ↑ p38 MAPK, pyroptosis, ↓ social behavior; NAC/vitamin E mitigate	[47,154,155,167]
**Anxiety, locomotion (in vivo: mice, zebrafish, *C. elegans*)**	PS-MP/NP (virgin, 0.1–5 µm), PLA-MP	0.1–250 µg/L; 10 mg/kg; 3–40 days	↑ anxiety, ↓ exploratory activity, ↑ ROS, DA/5-HT/adrenaline dysregulation; curcumin/NAC mitigate	[46,49,158,159,161,163]
**Prenatal/early exposure (in vivo: mice, zebrafish)**	PS-NP (virgin, 100 nm – 2 µm)	1 mg/day (GD1–PND21); 0.1–100 µg/mL; 17–21 days	Anxiety/depression in offspring, ↓ GABA, autism-like defects, ↑ local field potentials; NAC restores	[48,164,168]
**ADHD-like behavior (zebrafish, *C. elegans*)**	PS-MP/NP (virgin, 0.1–5 µm)	0.1–100 µg/mL; 1–7 days	↑ locomotion, +30% dopaminergic neurons, cholinergic/GABAergic neuron damage	[158,159]
**Neuroinflammation, microglia (in vivo/in vitro: mice, BV2)**	PS-MP/NP (virgin, 2 µm)	2–100 µg/mL; 2–10 mg/kg; 7 days	↑ PERK-NF-κB (HRAS), ↑ TNFα/IL-1β, autophagy/energy impairment, synaptic pruning defects	[161,165]
**Neurotransmitters, circadian rhythms (zebrafish, mice)**	PS-MP/NP (virgin), PP-MP	0.5–100 µg/mL; 0.01–1 mg/day; 7 days – 7 weeks	DA/5-HT/GABA/Glu dysregulation, ↑ aggressiveness, ↑ wakefulness; per1b/per2/cry1a dysregulation	[46,160,162]
**Cognitive impairment, memory (in vivo: mice)**	PS-MP (virgin, 2 µm)	0.01–1 mg/day; 4 weeks	↓ learning/memory (Morris water maze), ↓ ACh/CREB/BDNF, ↑ ROS; vitamin E mitigates	[167]
**Combined exposure (ozone, ketamine, cold stress)**	PS-NP (virgin, 60 nm), PP-MP	12.5 mg/kg + ozone; 0.1–100 µg/mL + ketamine; 10 mg/kg + cold	↑ neuroinflammation/pyroptosis, ketamine buffer, ↑ methionine effects; NAC mitigates	[49,155,157]
**Common signaling pathways**	PS-MP/NP, PP, PLA	Various	↑ ROS, ↓ EAAT2/SIRT1/BDNF, ↑ NLRP3/p38 MAPK/PERK,	[47,154,155,161,165]

			microRNA/lncRNA dysregulation, clathrin-endocytosis	

Table S9. Summary of experimental data on reproductive toxicity of micro- and nanoplastics: key models and mechanisms (n=32 sources). Abbreviations: MP – microplastics; NP – nanoplastics; PS-NP – polystyrene nanoplastic; PS-MP – polystyrene microplastic; PE-MP – polyethylene microplastic; PP-MP – polypropylene microplastic; PVC-MP – polyvinyl chloride microplastic; PET-MP – polyethylene terephthalate microplastic; PC – polycarbonate; virgin – pristine/untreated plastic particles (no additives or aging); ↑ – increase/increased; ↓ – decrease/decreased; LH – luteinizing hormone; FSH – follicle-stimulating hormone; ATP – adenosine triphosphate; IL-17A – interleukin 17A; Prm3 – protamine 3; Tnp1 – transition protein 1; Aurkc – aurora kinase C; Mettl14 – methyltransferase-like 14; Pmfbp1 – polyamine modulated factor 1 binding protein 1; Ggn – gametogenetin; Fsip2 – fibrous sheath interacting protein 2; E2 – estradiol; P4 – progesterone; HMGB1 – high mobility group box 1; TLR4 – Toll-like receptor 4; NOX2 – NADPH oxidase 2; ROS – reactive oxygen species; TGF-β – transforming growth factor beta; Juno – Juno izumo sperm-egg fusion protein; IL-6 – interleukin 6; PKA – protein kinase A; sEH – soluble epoxide hydrolase; UPRT – uracil phosphoribosyltransferase; CYP1A1 – cytochrome P450 1A1; GD – gestational day; Bcl-2 – B-cell lymphoma 2; Nrf2 – nuclear factor erythroid 2-related factor 2; Hippo – Hippo signaling pathway; MST1 – mammalian sterile 20-like kinase 1; LATS1 – large tumor suppressor kinase 1; YAP1 – Yes-associated protein 1; CTGF – connective tissue growth factor; Cyr61 – cysteine-rich angiogenic inducer 61; ER – endoplasmic reticulum; DEHP – di(2-ethylhexyl) phthalate; CNR1 – cannabinoid receptor 1; CRBN – cereblon; YY1 – Yin Yang 1; CYP2E1 – cytochrome P450 2E1; LHR – luteinizing hormone receptor; cAMP – cyclic adenosine monophosphate; StAR – steroidogenic acute regulatory protein; Wnt – Wingless/Integrated; PERK – protein kinase R-like endoplasmic reticulum kinase; eIF2α – eukaryotic initiation factor 2 alpha; ATF4 – activating transcription factor 4; CHOP – C/EBP homologous protein; PLZF – promyelocytic leukemia zinc finger; DAZL – deleted in azoospermia-like; GnRH – gonadotropin-releasing hormone.

**Model / Condition**	**Type of MP/NP**	**Dose / Duration**	**Key effects**	**Sources**
**Male fertility (in vivo: rats, mice)**	PS-NP/MP (virgin, 38–1000 nm)	0.015–40 mg/kg/day; 28–90 days	↓ testosterone/LH/FSH, oligospermia, asthenospermia, ↓ motility/ATP/DNA, ↑ IL-17A, dysbiosis	[51,52,199,201,202,204,207]
**Spermatogenesis, blood-testis barrier**	PS-NP (virgin, 25–100 nm)	0.01% (~1 mg/day); 28 days	Penetration into Leydig/Sertoli cells, ↓ Prm3/Tnp1/Aurkc/Mettl14, ↓ Pmfbp1/Ggn/Fsip2, inflammation	[204,207]
**Female fertility (in vivo: rats, mice)**	PS-NP/MP (virgin, 0.5–10 µm)	0.5–10 mg/kg/day; 28–90 days	Ovarian atrophy/fibrosis, ↓ reserve, ↑ atretic follicles, ↓ E2/P4, granulosa apoptosis	[171,173,174,185,186,189]

**Endometrium, uterus (in vivo/in vitro)**	PS-MP (virgin)	Various doses; 48 h – 6 weeks	Endometrial thinning, ↑ collagen, ↑ HMGB1/TLR4/NOX2/ROS/Notch/TGF-β; inhibitors ↓ fibrosis	[172,188,194]
**Oocytes, fertilization (in vivo: mice)**	PS-NP (virgin, 40 nm)	40 mg/kg/day; 30 days	DNA damage, spindle disruption, ↓ Juno, ↓ fertilization	[191]
**Placenta (in vitro/ex vivo: human, BeWo)**	PS-NP (virgin, 25–500 nm)	0.05–100 µg/mL; 24–72 h	↑ IL-6, Fe disruption, metabolism/cycle impairment, PKA/sEH/UPRT/CYP1A1 inhibition	[178,195,198]
**Transgenerational toxicity (in vivo: mice)**	PS-NP (virgin, 50–100 nm)	50–250 µg/200 µL; GD5.5–7.5	↑ miscarriage risk, ↓ embryos/implantation, immunosuppression, ↑ Bcl-2/caspase-3	[182,183]
**Accumulation in reproductive tissues**	Various MP (PE/PP/PS/PVC)	2.64×10^{14} particles (inhalation)	In placenta/testes/semen (0.72–7.02 µm), 2 MP/sample, ↓ sperm motility	[179,208,209]
**Oxidative stress, apoptosis (in vivo/in vitro)**	PS-NP (virgin, 20–100 nm)	1–200 µg/mL; 1–10 mg/kg; 48 h – 5 weeks	↑ ROS, ↓ Nrf2, ↑ Hippo (MST1/LATS1/YAP1), ↓ CTGF/Cyr61, granulosa apoptosis	[173,190,193]
**ER stress, inflammation (in vivo: ovaries)**	PS-MP (virgin, 876 nm)	2.5–10 mg/kg/day; 45 days	↑ ROS, ER stress, apoptosis, ovarian atrophy	[185]
**Combined toxicity (DEHP)**	PS-NP + DEHP	100 µg/L + 200 mg/kg	↑ ROS, DNA damage, cell cycle arrest (CNR1/CRBN/YY1/CYP2E1)	[192]
**Metabolomic changes (in vivo: pregnancy)**	PS-NP (virgin)	10^2–10^6 ng/L; entire pregnancy	Biotin/lysine/glycolysis changes	[197]
**Clinical data (placenta, semen)**	PS/PE/PVC	Detected in samples	↓ placental weight, immune disruptions, presence in semen → ↓ motility	[180,209]
**In silico (placenta)**	PS-MP (1–100 nm)	—	sEH/UPRT/B3GAT1/SULT/NAT2/CYP1A1 inhibition; PC > PET > PS	[196]

**Marine organisms (*Tegillarca granosa*)**	PS-MP (0.5–5 μm)	0.069–0.69 mg/L	↓ sperm motility/ATP/viability/DNA integrity	[200]
**Common signaling pathways**	PS-NP/MP, PE/PP/PVC	Various	↓ LHR/cAMP/PKA/StAR, ↑ Wnt/β-catenin, ↑ PERK/eIF2α/ATF4/CHOP, ↓ PLZF/DAZL, ↑ GnRH	[51,52,171,189,191]

Table S10. Summary of experimental data on nephrotoxicity of micro- and nanoplastics: key models and mechanisms (n=27 sources). Abbreviations: MNPs – micro- and nanoplastics; MP – microplastics; NP – nanoplastics; PS-MP – polystyrene microplastic; PS-NP – polystyrene nanoplastic; PE-MP – polyethylene microplastic; PP-MP – polypropylene microplastic; PET-NP – polyethylene terephthalate nanoplastic; PVC-MP – polyvinyl chloride microplastic; virgin – pristine/untreated plastic particles (no additives or aging); DEHP – di(2-ethylhexyl) phthalate; BaP – benzo[a]pyrene; HFD – high-fat diet; ↑ – increase/increased; ↓ – decrease/decreased; ROS – reactive oxygen species; MDA – malondialdehyde; H_2O_2 – hydrogen peroxide; SOD – superoxide dismutase; CAT – catalase; GSH – glutathione; GAPDH – glyceraldehyde-3-phosphate dehydrogenase; ER – endoplasmic reticulum; AMPK – AMP-activated protein kinase; ULK1 – Unc-51-like autophagy activating kinase 1; MAPK – mitogen-activated protein kinase; AKT – protein kinase B; mTOR – mechanistic target of rapamycin; p-EIF2α – phosphorylated eukaryotic initiation factor 2 alpha; p-IRE1α – phosphorylated inositol-requiring enzyme 1 alpha; ATF6 – activating transcription factor 6; HO-1 – heme oxygenase 1; NLRP3 – NOD-like receptor family pyrin domain containing 3; ZO-2 – zonula occludens-2; TGF-β1 – transforming growth factor beta 1; CD63 – cluster of differentiation 63; CD81 – cluster of differentiation 81; PAI-1 – plasminogen activator inhibitor 1; CTGF – connective tissue growth factor; BUN – blood urea nitrogen; KIM-1 – kidney injury molecule 1; GPx – glutathione peroxidase; T-AOC – total antioxidant capacity; NF-κB – nuclear factor kappa B; TNF-α – tumor necrosis factor alpha; iNOS – inducible nitric oxide synthase; IL-1β – interleukin 1 beta; IL-6 – interleukin 6; RIP1 – receptor-interacting protein kinase 1; RIP3 – receptor-interacting protein kinase 3; MLKL – mixed lineage kinase domain-like protein; Nrf2 – nuclear factor erythroid 2-related factor 2; SLC7A11 – solute carrier family 7 member 11; GPX4 – glutathione peroxidase 4; LC3 – microtubule-associated protein 1 light chain 3; NCOA4 – nuclear receptor coactivator 4; mtROS – mitochondrial ROS; Klotho – anti-aging protein Klotho; Wnt – Wingless/Integrated; PI3K-Akt – phosphoinositide 3-kinase/protein kinase B; IL-17 – interleukin 17; PF4 – platelet factor 4; NAC – N-acetylcysteine; MitoTEMPO – mitochondria-targeted antioxidant; NPC – nephron progenitor cells; Notch – Notch signaling pathway; TCA – tricarboxylic acid (cycle); TG – triglycerides; PL – phospholipids; BP – blood pressure; OS – oxidative stress; Bax – Bcl-2-associated X protein; EMT – epithelial–mesenchymal transition; ECM – extracellular matrix; C5a – complement component 5a; C5aR – C5a receptor; AKI – acute kidney injury; PERK – protein kinase R-like endoplasmic reticulum kinase; CHOP – C/EBP homologous protein.

Model / Condition	Type of MNP	Dose / Duration	Key effects	Sources
**In vitro (HEK293, HK-2, Caco-2,**	PS-MP/NP (virgin, 30–5000 nm) + DEHP/BaP	3 ng/mL – 800 μg/mL	↑ Uptake/adhesion, ↑ ROS/MDA/H_2O_2, ↓ SOD/CAT/GSH/GAPDH, ER stress/autophagy	[25,213,216,217,218,223,226,236]

**HepG2, NRK-49F)**		; 24–72 h	(AMPK/ULK1, MAPK/AKT/mTOR, p-EIF2α/IRE1α/ATF6), apoptosis, ↓ HO-1/NLRP3/ZO-2, ferroptosis (TGF-β1), ↑ exosomes ($CD63^+$/$CD81^+$), fibrosis (collagen1/PAI-1/CTGF)	
**In vivo: mice (oral)**	PS-NP/MP (virgin, 50–4000 nm) + Cd/As/gentamicin/HFD	0.1–25 mg/kg/day; 4–18 weeks	↓ Body/renal index, ↑ BUN/creatinine/KIM-1/cystatin C/MDA, ↓ SOD/GPx/GSH, tubular atrophy/glomerular collapse, necrosis/epithelial detachment/loss of brush border, inflammation (NF-κB/TNF-α/IL-1β/IL-6), ferroptosis/mitophagy (↓ Nrf2/SLC7A11/GPX4, ↑ LC3/NCOA4/mtROS), fibrosis/EMT (Klotho/Wnt/β-catenin/TGF-β1), ↑ PI3K-Akt/MAPK/IL-17, $PF4^+$ macrophages, synergy with toxicants; NAC/MitoTEMPO/betaine mitigate	[216,219,220,221,222,224,225,229,230,233,235]
**In vivo: chickens/rats (oral)**	PS-MP (virgin, 30 nm – 10 μm) + butyrate	1–100 mg/L feed; 10 mg/L; 6–12 weeks	Mitochondrial damage, ↓ SOD/CAT/GSH/T-AOC, ↑ MDA/NF-κB/TNFα/iNOS/IL-1β/IL-6, necroptosis (RIP1/RIP3/MLKL), hypertension, dysfunction; butyrate ↓ OS, modulates microbiota	[31,227]
**Human kidney organoids (in vitro)**	PS-MP (virgin, 1 μm)	Not specified; exposure	Adhesion to NPC/glomerular accumulation, ↓ size, ROS-apoptosis, ↓ Notch/glycolysis, ↑ TCA cycle, metabolic reprogramming, tubular disruption	[231,234]
**In vivo: mice (oral, other polymers)**	PP-MP (<5 μm), PET-NP (virgin)	100–1000 μg/L; 200	↑ ROS/MDA, ↓ GSH, lipidome/TG/PL changes, podocyte effacement, glomerular hypotrophy,	[232,233]

		mg/kg; 7–42 days	tubular degeneration; betaine mitigates	
**Clinical/field: humans (kidneys, urine, transplants, hemodialysis)**	PE/PS/PVC-MP/NP	25.7–98.9 µg/g; 0.0021–3768 particles/week	Presence/deposition in kidneys/urine/transplants, BP correlation (non-significant), ↑ exposure risk via dialysate, potential blood/kidney accumulation	[210,228,238]
**Combined/ synergistic toxicity**	PS-NP + Cd/As/gentamicin/BaP/HFD	0.5–1.5 mg/kg + toxicant; 15–60 days	↑ OS/inflammation/ferroptosis/fibrosis/dysfunction, synergy (NF-κB/TNF-α/Bax/caspases, lipid disruption, profibrotic/protumorigenic environment)	[220,221,225,226,235]
**Oxidative stress, apoptosis, autophagy**	PS-MP/NP (virgin)	Various; 24 h – 8 weeks	↑ ROS/MDA, ↓ antioxidants, mitochondrial damage, ER stress, apoptosis/autophagy/necroptosis, inflammation	[25,31,213,216,217,218, 224,229]
**Fibrosis, EMT, ECM remodeling**	PS-MP/NP (virgin)	60 µg/mL – 10 mg/L; 28 days – 18 weeks	↑ TGF-β1/collagen1/PAI-1/CTGF, fibroblast activation, interstitial fibrosis, EMT (Klotho/Wnt/β-catenin), ECM changes	[223,224,230,235,236]
**Barrier disruption, complement**	PS-MP (virgin, 0.5–5 µm)	0.5–2 mg/kg/day; 8 weeks	Intestinal barrier disruption, ↑ C5a in urine/C5aR in kidneys, acute kidney injury (AKI)	[237]
**Common signaling pathways**	PS-MP/NP, PE/PP/PET	Various	↑ ROS/AMPK/ULK1/NF-κB/MAPK/PI3K-Akt/IL-17, ↓ Nrf2/GPX4/SOD, ↑ PERK/eIF2α/ATF4/CHOP, ferroptosis (SLC7A11/LC3), ER stress (p-IRE1α/ATF6)	[25,31,216,219,220,221, 224,235]

Table S11. Summary of experimental data on gastrointestinal toxicity of micro- and nanoplastics: key models and mechanisms (n=35 sources). Abbreviations: MNPs – micro- and nanoplastics; MP – microplastics; NP – nanoplastics; PS-MP – polystyrene microplastic; PS-NP – polystyrene nanoplastic; PE-MP – polyethylene microplastic; PP-MP – polypropylene microplastic; PET-MP – polyethylene terephthalate microplastic; PC – polycarbonate; PA – polyamide; virgin – pristine/untreated plastic particles (no additives or aging); ↑ – increase/increased; ↓ – decrease/decreased; ROS – reactive oxygen species; LD – lipid droplets; NF-κB – nuclear factor kappa B; NLRP3 – NOD-like receptor family pyrin domain containing 3; MLCK – myosin light-chain kinase; TRPV1 – transient receptor potential vanilloid 1; iNOS – inducible nitric oxide synthase; IL-1β – interleukin 1 beta; IL-8 – interleukin 8; Ras – rat sarcoma; ERK – extracellular signal-regulated kinase; CDK4 – cyclin-dependent kinase 4; AMPK – AMP-activated protein kinase; ULK1 – Unc-51-like autophagy activating kinase 1; ZO-1 – zonula occludens-1; TLR4 – Toll-like receptor 4; P53 – tumor protein p53; NAC – N-acetylcysteine; MCC950 – NLRP3 inhibitor; ML-7 – MLCK inhibitor; DSS – dextran sulfate sodium; HFD – high-fat diet; CTX – cyclophosphamide; FMT – fecal microbiota transplantation; EpH4 – mouse mammary epithelial cell line; IBD – inflammatory bowel disease; MDA – malondialdehyde; MAPK – mitogen-activated protein kinase; AP-1 – activator protein 1; IRF5 – interferon regulatory factor 5; RhoA – Ras homolog family member A; FoxO – forkhead box O; Cr – chromium; Pb – lead; GI – gastrointestinal.

Model / Condition	Type of MNP	Dose / Duration	Key effects	Sources
**In vitro (Caco-2, GES-1, HepG2, HT-29)**	PS-NP/MP (virgin, 50–150 nm, 0.2–60 µm); PE/PP/PET/PC	0.1–1000 µg/mL; 4–48 h	↑ Uptake (NP > MP), ↓ viability, ↑ ROS/superoxide/LD/DNA damage, ↑ NF-κB/NLRP3/MLCK/TRPV1/iNOS/IL-1β/IL-8/Ras/ERK/CDK4/Cyclin D1, autophagy/lipophagy block (↓ AMPK/ULK1), reversible barrier loss, ↑ *H. pylori* adhesion	[53,60,249,259,268,269,270,271]
**In vivo: mice (oral, healthy/DSS-colitis/HFD)**	PS-MP/NP (virgin, 50 nm – 10 µm); PE/PP-MP (4–600 µm)	18 µg/kg – 30 g/kg feed (~0.21 g/day); 4–90 days	↑ Permeability/inflammation/cytokines (NF-κB/TLR4/Notch/P53), ↓ ZO-1/occludin/mucin/*Lactobacillus*, dysbiosis, ↑ ROS/apoptosis/colitis, insulin resistance, gut–liver axis; NAC/MCC950/ML-7/melatonin restore	[53,56,59,61,251,257,260,261,262,263,264,265,266,273,275]

**In vivo: mice (lactation/ FMT) + EpH4**	PS-MP (virgin, <5 µm)	3–30 mg/L (in vivo); 0.15–0.6 mg/mL (in vitro); 30–48 h	↑ Intestinal/blood-milk barrier permeability, colon/mammary inflammation, ↑ lipid metabolism/ferroptosis, mammary accumulation, gut–mammary axis via microbiota (FMT transmission)	[57]
**Combined toxicity (CTX/ethanol/HFD)**	PS-MP (virgin) + xenobiotics	0.1–80 mg/kg; 5–13 weeks	↑ Hepatotoxicity/steatosis/permeability, ↓ antioxidants, dysbiosis ↔ enhanced liver/GI damage	[56,254,275]
**In vivo: rats/piglets (oral)**	PE/PP/PET-MP (virgin, 4–70 µm)	0.1–15 mg/kg/day; 0.1–1 g/day; 4–5 weeks	↑ TLR4/NF-κB/ROS/apoptosis, ↓ TJ proteins, ↑ 86 genes/diabetes-like response; melatonin restores	[258,262,263]
**Clinical: humans (feces/IBD, stomach, liver, tumors)**	9–15 types MP (PP/PET/PE/PA/PS; 4–1299 µm)	20–41.8 particles/10 g feces; 9.4 particles/person; accumulation in cirrhosis/tumors	100% positive samples, ↑ MP in IBD (41.8 vs 28 particles/g), disease severity, accumulation in cirrhotic liver/tumors > healthy tissue; exposure biomarker	[58,243,248,253,277]
**Dysbiosis and microbiota-dependent effects**	PS/PE-MP (virgin)	100 µg/L – 30 g/kg; 2–13 weeks	Dysbiosis (females > males), ↓ *Lactobacillus*/*Parabacteroides*/*Alistipes*, ↑ *Staphylococcus*, FMT transmission, gut–liver/mammary axes	[56,57,265,266,274]
**GI barrier disruption**	PS/PE/PP-MP (virgin, 0.2–70 µm)	1 mg/kg – 600 µg/day; 4–15 weeks	↓ ZO-1/occludin/claudin-1/mucin, ↑ MLCK, villus deformation/pyknosis, ↑ permeability	[53,57,262,275]
**Oxidative stress and inflammation**	PS/PE/PP-MP/NP (virgin)	Various; 4 h – 13 weeks	↑ ROS/MDA/superoxide, ↓ antioxidants, ↑ NF-	[53,60,255,259,263,268,271]

			κB/NLRP3/TLR4/MAPK/cytokines/IL-1β/IL-8, apoptosis	
**Metabolic disruptions**	PS-MP/NP (virgin)	10^6 particles/day – 80 mg/L; 1–15 weeks	↑ Glucose/insulin, insulin resistance, lipophagy block → lipid accumulation, ↓ lipid digestion (heteroaggregates with lipases)	[60,61,258,273]
**Other polymers and forms**	PET/PE/PP-MP (virgin)	3×10^4 particles/3 days; 0.01–1 mg/kg; 4–12 weeks	Transcriptome/microbiota changes (no histopathology), Cr/Pb release	[250,256]
**In vivo: fish (diet)**	MP (various)	1–20 particles/individual (~50–60% individuals)	↑ Cytokines, antioxidant enzymes	[272]
**Common signaling pathways**	PS/PE/PP/PET-MP/NP	Various	↑ ROS → NF-κB/NLRP3/IL-1β/MLCK/TLR4/AP-1/IRF5/Notch/P53/RhoA/F-actin/TRPV1, ↓ AMPK/ULK1, ↑ MAPK/ERK/CDK4/Cyclin D1	[53,60,249,261,263,268]
**Aged mice + GI corona**	PS-MP (with corona)	10^3–10^{12} particles/L; 10 days	↑ AMPK/FoxO, ↑ ROS/liver inflammation	[255]
**Simulated GI tract (SimuGIT)**	PE/PP-MP	0.625 mg/mL; 4 h	23% Cr/Pb release	[250]
**Pancreatitis + MP**	PS-MP (virgin)	100–1000 µg/L; 28 days	Enhanced pancreatic inflammation	[257]
**General mechanisms**	All MNP types	Various	Dysbiosis → metabolic/inflammatory axes, ↑ permeability → systemic effects, xenobiotic combination ↑ risk	[56,57,61,275]
